# Synthesis, Docking Studies and Biological Activity of New Benzimidazole- Triazolothiadiazine Derivatives as Aromatase Inhibitor

**DOI:** 10.3390/molecules25071642

**Published:** 2020-04-02

**Authors:** Ulviye Acar Çevik, Betül Kaya Çavuşoğlu, Begüm Nurpelin Sağlık, Derya Osmaniye, Serkan Levent, Sinem Ilgın, Yusuf Özkay, Zafer Asım Kaplancıklı

**Affiliations:** 1 Department of Pharmaceutical Chemistry, Faculty of Pharmacy, Anadolu University, Eskisehir 26470, Turkey; uacar@anadolu.edu.tr (U.A.Ç.); bnsaglik@anadolu.edu.tr (B.N.S.); dosmaniye@anadolu.edu.tr (D.O.); serkanlevent@anadolu.edu.tr (S.L.); zakaplan@anadolu.edu.tr (Z.A.K.); 2 Doping and Narcotic Compounds Analysis Laboratory, Faculty of Pharmacy, Anadolu University, Eskisehir 26470, Turkey; 3 Department of Pharmaceutical Chemistry, Faculty of Pharmacy, Bülent Ecevit University, Zonguldak 67100, Turkey; betulkaya@anadolu.edu.tr; 4 Department of Pharmaceutical Toxicology, Faculty of Pharmacy, Anadolu University, Eskisehir 26470, Turkey; silgin@anadolu.edu.tr

**Keywords:** aromatase, MCF-7, NIH3T3, benzimidazole, triazolothiadiazine, docking, ADME

## Abstract

In the last step of estrogen biosynthesis, aromatase enzyme catalyzes the conversion of androgens to estrogens. Aromatase inhibition is an important way to control estrogen-related diseases and estrogen levels. In this study, sixteen of benzimidazole-triazolothiadiazine derivatives have been synthesized and studied as potent aromatase inhibitors. First, these compounds were tested for their anti-cancer properties against human breast cancer cell line (MCF-7). The most active compounds **5c**, **5e**, **5k**, and **5m** on MCF-7 cell line were subject to further in vitro aromatase enzyme inhibition assays to determine the possible mechanisms of action underlying their activity. Compound **5e** showed slight less potent aromatase inhibitory activity than that of letrozole with IC_50_ = 0.032 ± 0.042 µM, compared to IC_50_ = 0.024 ± 0.001 µM for letrozole. Furthermore, compound **5e** and reference drug letrozole were docked into human placental aromatase enzyme to predict their possible binding modes with the enzyme. Finally, ADME parameters (absorption, distribution, metabolism, and excretion) of synthesized compounds (**5a**–**5p**) were calculated by QikProp 4.8 software.

## 1. Introduction

Aromatase is a member of the cytochrome P450 enzyme that catalyzes the biosynthesis of estrogens from androgens [1,2]. Estrogen levels have been shown to be higher in breast cancer cells. Inhibition of aromatase is one of the effective current therapeutic strategies for controlling estrogen-dependent breast cancer. Therefore, one of the commonly used classes of drugs for the management of estrogen-dependent cancer is aromatase inhibitors (AIs) [1,3,4].

Aromatase inhibitors that have been used clinically can be categorized as first-, second-, and third generations based on their evolution time or steroidal or steroidal or non-steroidal aromatase inhibitors (NSAIs) based on their structural similarity with steroids [5,6].

Aminoglutethimide is the prototype nonsteroidal inhibitor of aromatase. Problems with the side effects and selectivity of aminoglutethimide led to the development of the second-generation of nonsteroidal aromatase inhibitor (Fadrazole bearing imidazole structure) [7]. However, this compound still has some nonselective inhibitory activity with respect to progesterone, corticosterone, and aldosterone biosynthesis. Competitive nonsteroidal inhibitors can also be constructed with a triazole ring, which is found in the third generation of aromatase inhibitors [8,9,10]. Most of the nonsteroidal aromatase inhibitors of therapeutic importance act covalently bind to the substrate-binding site of aromatase by coordinate the heme iron [11,12,13]. Especially, the heterocyclic nitrogen atom of triazole and imidazole plays an important role by coordinating with the heme iron of the aromatase enzyme [12,13]. Some studies have shown that imidazole and triazole derivatives have promising aromatase inhibition [14,15,16,17].

Recent clinical studies have shown that aromatase inhibitors, especially the third-generation, are more effective than fulvestrant and tamoxifen because of their lower side effects and higher clinical efficacy. Nevertheless, the development of acquired resistance after prolonged AIs therapy, undesirable side effects (bone loss, cardiac events, increased rash, insomnia, headaches, and arthralgia) limits their use in clinical practice. Thus, the search for new potent molecules that impair cancer growth, strongly inhibit aromatase enzyme, and present fewer side effects is of major importance [18,19,20,21].

In the previous study, 3-[4-(5-methyl-1*H*-benzo[*d*]imidazol-2-yl)phenyl]-6- (substituted phenyl) -7H-[1,2,4] triazolo [3,4-b] [1,3,4]thiadiazines derivatives have been synthesized and promising compounds have been obtained that need further development as a new class of aromatase inhibitors [22]. In this study, new benzimidazole-triazolothiadiazine derivatives were synthesized and structure of these compounds was characterized by spectroscopic data. Their antiproliferative activities against MCF-7 were evaluated. To identify the possible modes of action, aromatase inhibition experiments were performed for the most active compounds against the MCF-7 cell line. Finally, in silico prediction of pharmacokinetic profiles (ADME) were calculated for physicochemical properties of these drug candidates.

## 2. Results and Discussion

### 2.1. Chemistry

**Scheme 1:**
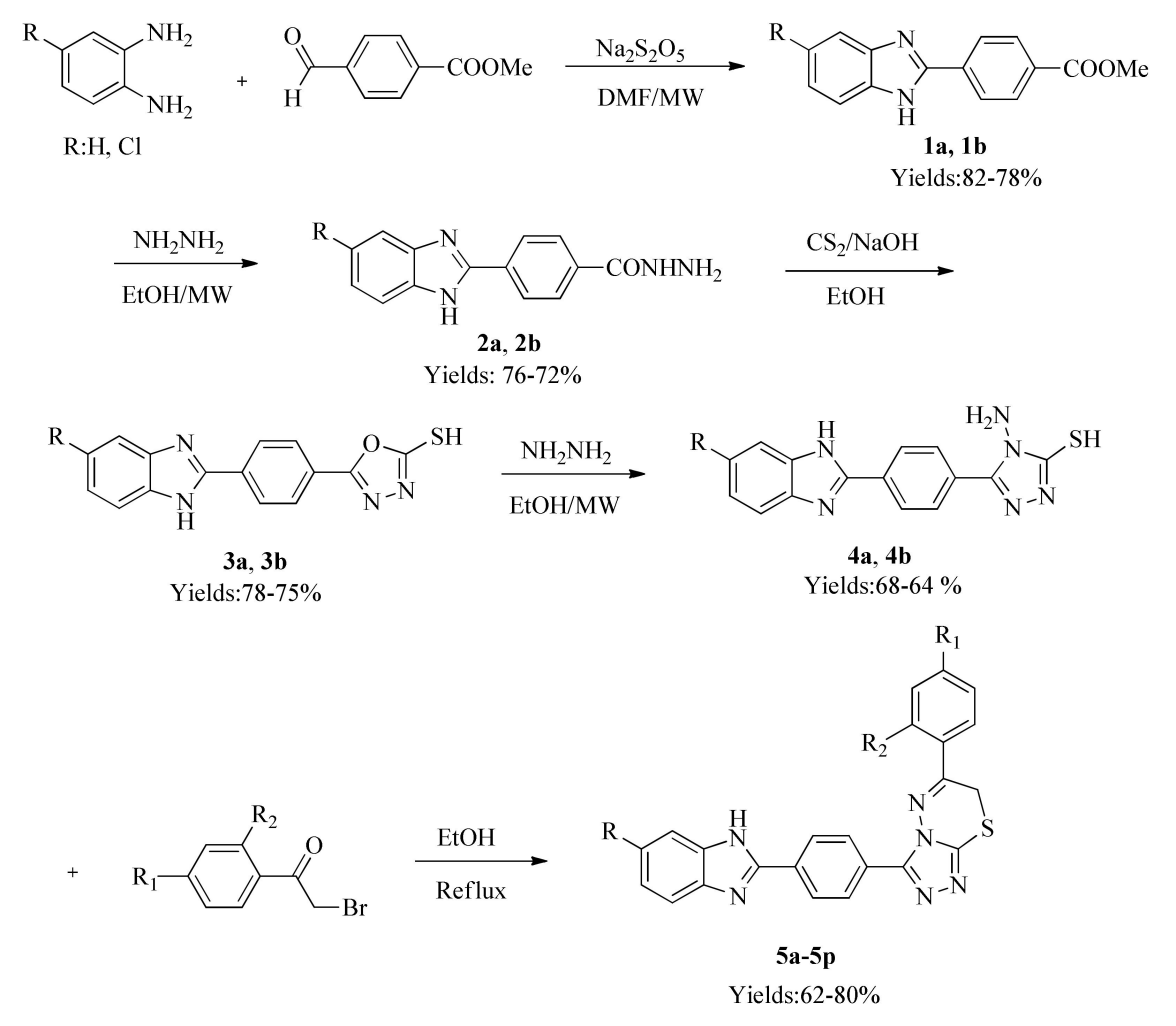
The reaction sequence for the synthesis of the compounds (**5a**–**5p**).

Synthesis of the target compounds was accomplished according to the steps illustrated in Scheme 1. Substituents of the synthesized compounds (**5a**–**5p**) were shown in Table 1. The starting intermediated compound (**1a**,**1b**) was prepared by the reaction of 1,2-phenylenediamine and sodium metabisulfite with 4-formylbenzoic acid methyl ester as described in a previous study [23]. The ester derivative (**1a**,**1b**) reacted with hydrazine hydrate producing compounds **2a**, **2b** in a microwave synthesis reactor. Then, the compounds-(5-substitüe-1*H*-benzimidazol-2-yl)benzoic acid hydrazide derivatives (**2a**,**2b**) were treated with carbon disulfide in NaOH solution affording, after acidic treatment, compounds **3a**, **3b**. The reaction of compound **3a, 3b** with hydrazine hydrate in the presence of ethanol produced compounds **4a**, **4b**. Then cyclization of compounds **4a**, **4b** with appropriate phenacyl bromide in the presence of anhydrous ethanol provided the desired final products (**5a**–**5p**). The characterization of these new derivatives was done by their spectroscopic (^1^H NMR, ^13^C NMR and Mass) data. ^1^H NMR and ^13^C NMR spectra of the synthesized compounds were observed at the expected region. Mass spectra (HRMS) of all the synthesized compounds showed that the molecular ion [M + H]^+^ peak is in agreement with their molecular formula (see Supplementary Materials).

### 2.2. Cytotoxicity Assay

The newly synthesized compounds were evaluated for their in vitro anticancer potential against MCF-7 breast cancer cell lines by MTT assay [24] using cisplatin as a reference standard. The 50% inhibitory concentration (IC_50_) values were determined for these compounds. In addition, the cytotoxic activities of compounds **5c**, **5e**, **5k**, and **5m** were assessed against healthy NIH3T3 [25] cells, in order to express the selectivity toward carcinogenic cells. The IC_50_ values of test compounds were determined as the mean IC_50_ of 4 independent experiments. Results are presented in Table 2, as IC_50_ values are in μM and revealed that some of the tested compounds were remarkably more cytotoxic than the cisplatin against MCF-7 cell lines. Concerning MCF-7 cell line, the most potent compounds were the 4-cyano derivatives **5e** and **5m** with IC_50_ = 0.016 ± 0.001 and 0.018 ± 0.001 μM, respectively compared to IC_50_ = 0.020 ± 0.009 μM for the reference drug cisplatin. Furthermore, the most promising activity was observed for the compounds **5c** and **5k** bearing 4-fluorophenyl derivatives with IC_50_= 0.119 ± 0.005 and 0.110 ± 0.005 μM, respectively compared to IC_50_ = 0.020 ± 0.009 μM for the reference drug cisplatin.

Benzimidazole-triazolothiadiazine derivatives led to a promising increase in the anti-proliferative activity. The synthesized compounds can be divided into two groups as nonsubstituted benzimidazole and 5-chlorobenzimidazole. Test compounds showed variable activities against MCF-7. In particular, compounds **5e** and **5m** displayed the highest cytotoxic activity against MCF-7 cell line. The most active compounds **5e** and **5m** carried a 4-cyanophenyl substituent. The chlorine substituent in the fifth position (**5m**) of the benzimidazole ring did not significantly affect the activity. Compounds **5c** and **5k** carrying fluorine substituents in the fourth position of the phenyl ring showed lower activity than compounds **5e** and **5m** bearing cyano group in the fourth position of the phenyl ring.

### 2.3. Aromatase Inhibition Assay

The in vitro anti-aromatase activity of the most active compounds **5c**, **5e**, **5k,** and **5m** was valued using commercial fluorometric assay kit (Aromatase-CYP19A Inhibitor Screening kit, Bio Vision) with letrozole as the reference drug. Results are presented in Table 3. The IC_50_ values of compounds were in the sub-micromolar range (2.276 ± 0.106–0.03 2 ± 0.001 µM). The best value was shown by compound **5e** with IC_50_ value (0.032 ± 0.001 µM). 

### 2.4. Molecular Docking

**Figure 1:**
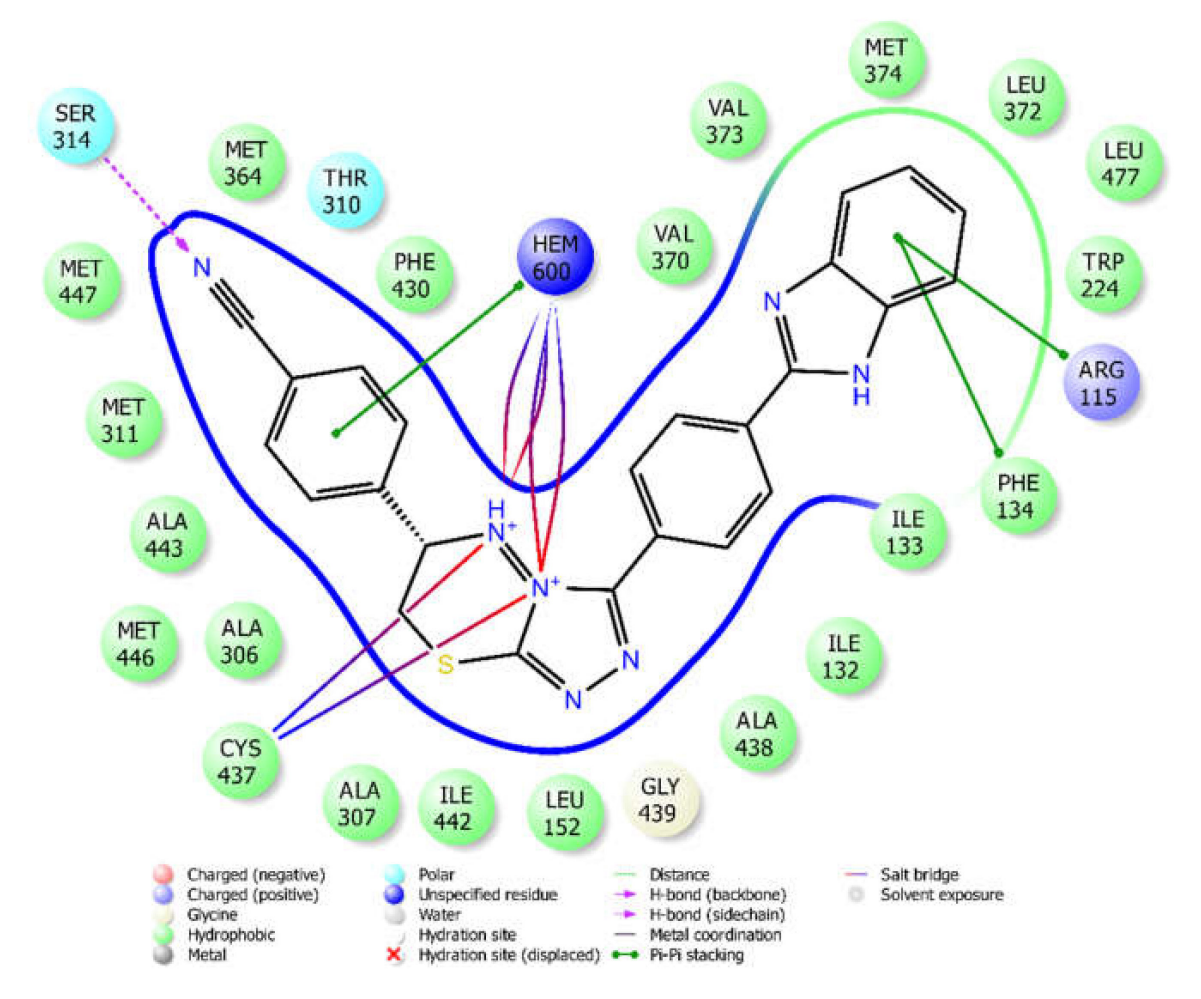
Two-dimensional interaction mode of compound **5e** in the enzyme active site (Human aromatase PDB Code: 3EQM).

**Figure 2:**
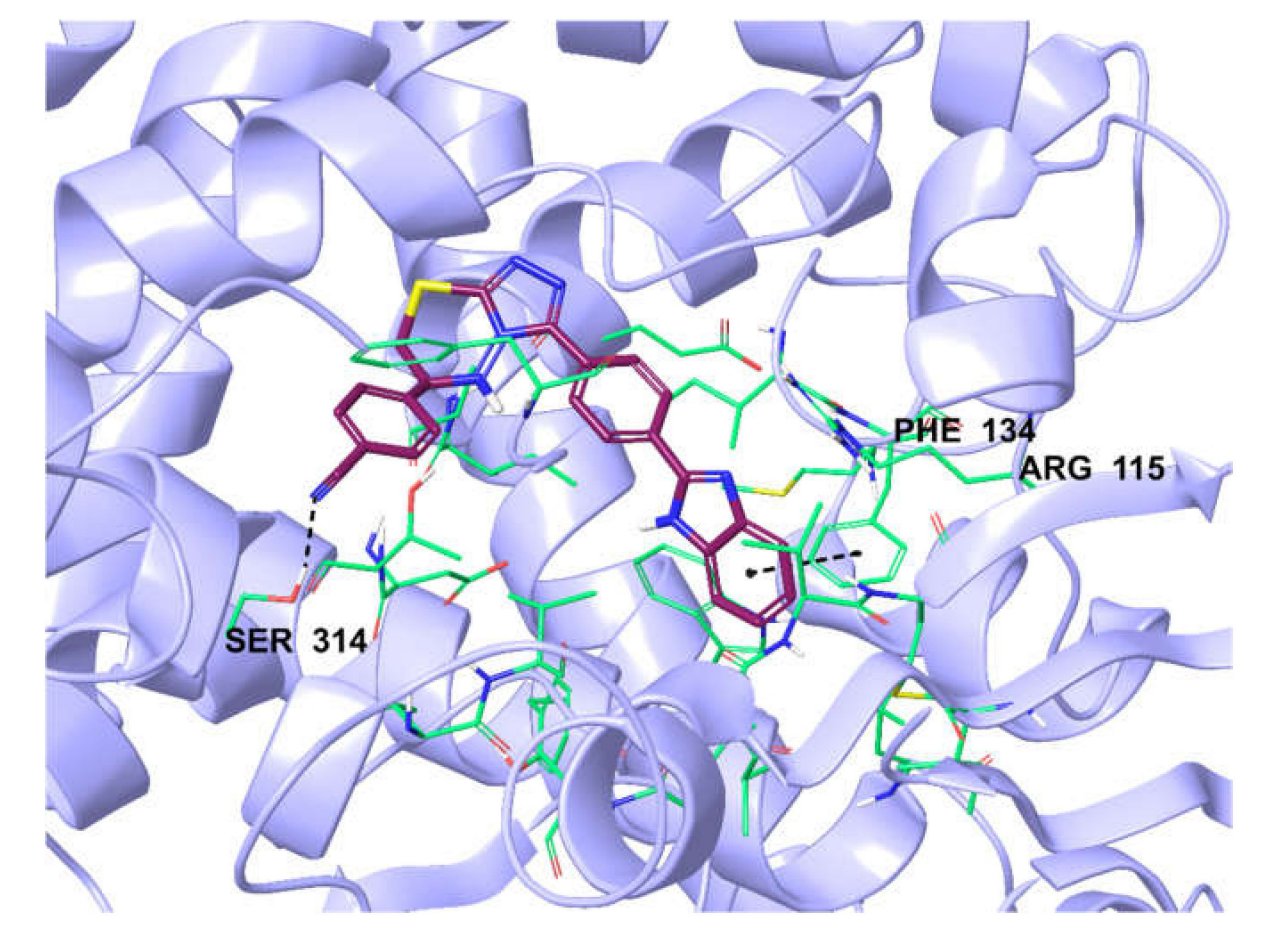
The interacting mode of compound **5e** in the active region of human aromatase. The inhibitor, colored with maroon, and the important residues, colored with purple, in the active site of the enzyme are presented by tube model.

**Figure 3:**
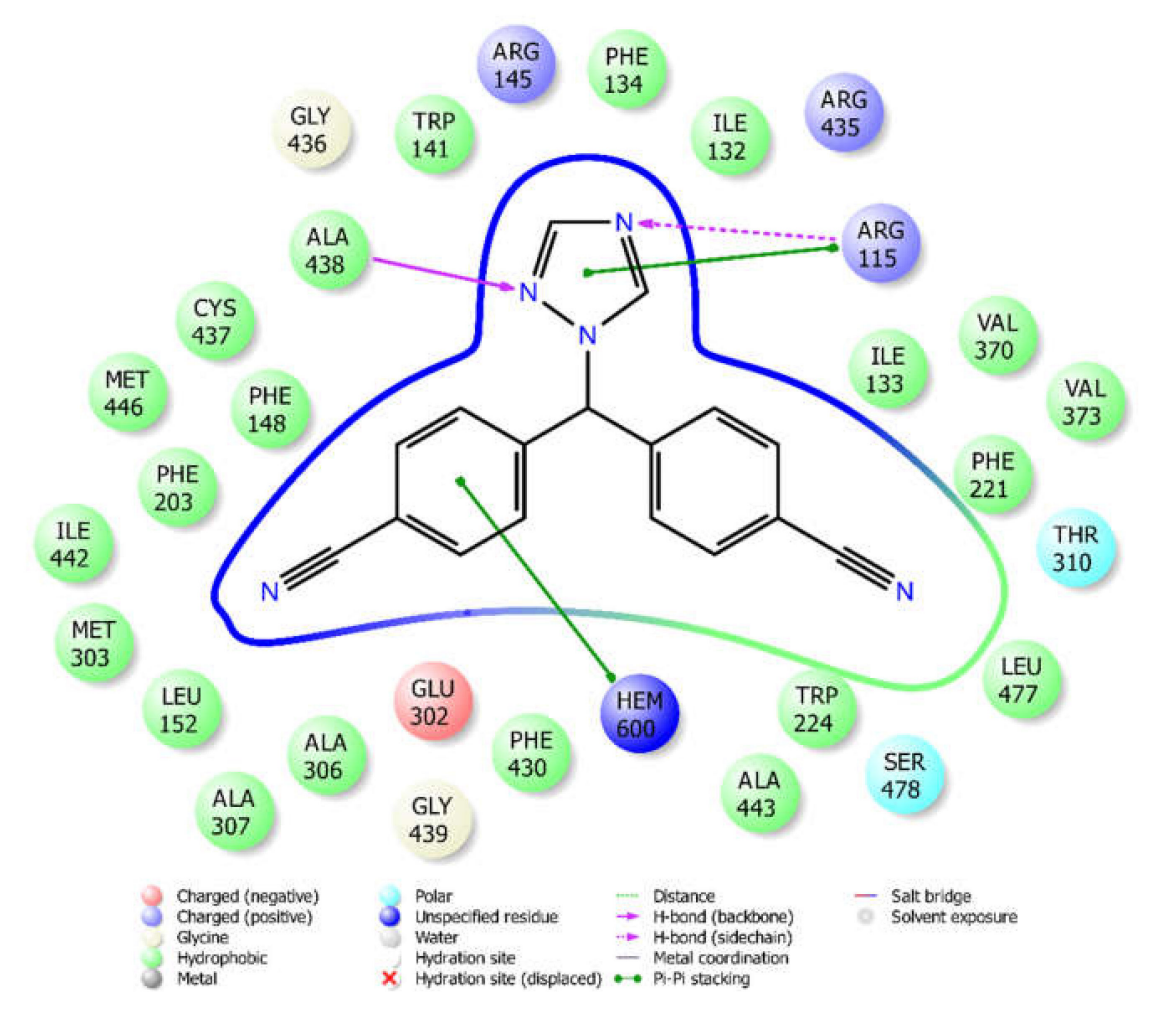
Two-dimensional interaction mode of letrozole in the enzyme active site (Human aromatase PDB Code: 3EQM).

**Figure 4:**
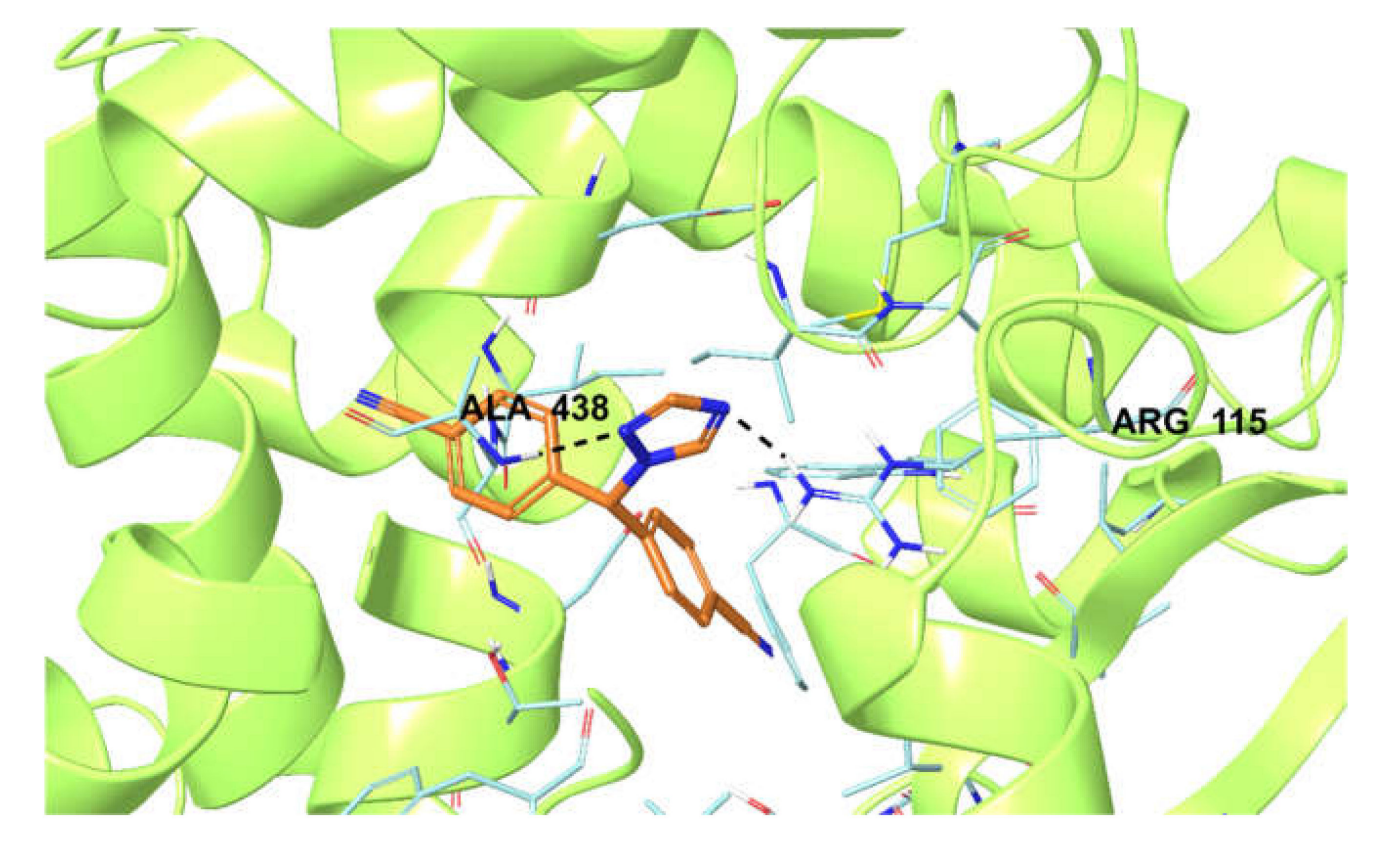
The interacting mode of letrozole in the active region of human aromatase. The inhibitor, colored with orange, and the important residues, colored with turquoise, in the active site of the enzyme are presented by tube model.

After the most active derivative was selected as compound **5e** according to in vitro aromatase enzyme inhibition assay and letrozole, molecular docking studies were performed to explain its binding modes with human aromatase enzyme active site. For this purpose, the crystal structure of human aromatase enzyme (PDB ID: 3EQM) [26] was retrieved from the Protein Data Bank server (www.pdb.org). The 2D and 3D docking poses of compound 5e are presented in Figure 1 and Figure 2. The docking poses of letrozole are presented in Figure 3 and Figure 4.

According to docking poses, it is understood that compound 5e displays compatible settlement with the enzyme active region. The benzimidazole ring in the structure forms two π–π interactions with Arg115 and Phe134. Also, it can be seen from 2D docking pose, this compound is in interaction with Hem molecules and Cys437 via its nitrogen atoms of triazolothiadiazine ring by salt bridge. There is another π–π interaction between 4-cyanophenyl ring and HEM molecule. The nitrogen atom of cyano group at C-4 position of phenyl ring interacts with hydroxyl of Ser314 doing hydrogen bonding. It is thought that this interaction is important for compound **5e** in terms of explaining its inhibitory activity. It is seen that the presence of an electron withdrawing group such as cyano at this position is a positive contribution to the activity.

As seen in the docking poses, letrozole is settled down in the enzyme active site properly. For letrozole, it is seen that benzonitrile ring creates π–π interaction with HEM molecule. Another π–π interaction is observed between 1,2,4-triazole ring and Arg115. Also, this 1,2,4-triazole ring forms two hydrogen bonds via its second and fourth nitrogen atoms with amino groups of Ala438 and Arg115, respectively. 

### 2.5. Theoretical Determination of ADME Properties

QikProp allows to provide acceptable ranges for comparing the predicted properties of compounds with those of 95% of known drugs and estimate drug-likeness properties. The drug-likeness of a compound was assessed according to Jorgensen’s rule of three [27], which regards PCaco (>22 nm/s), logS (>−5.7), primary metabolites (PM) (<7), and Lipinski’s rule of five [28], which considers number of hydrogen bond acceptors (≤10) and donors (≤5), molecular weight (<500 Da), and octanol/water partition coefficient (≤5).

Table 4. presents the predicted ADME properties of all compounds. According to Lipinski’s rule of five and Jorgensen’s rule of three, all compounds (**5a**–**5p**) are in accordance with the rule by causing no more than one violation. Consequently, according to predictions of ADME properties, it can be suggested that the active compounds may have a good pharmacokinetic profile.

## 3. Materials and Methods 

### 3.1. Chemistry

Whole chemicals employed in the synthetic procedure were purchased from Sigma-Aldrich Chemicals (Sigma-Aldrich Corp., St. Louis, MO, USA) or Merck Chemicals (Merck KGaA, Darmstadt, Germany). Melting points of the obtained compounds were determined by MP90 digital melting point apparatus (Mettler Toledo, OH, USA) and were uncorrected. Microwave syntheses were realized by using a Monowave 300 high-performance microwave reactor (Anton-Paar, Austria). ^1^H NMR and ^13^C NMR spectra of the synthesized compounds were registered by a Bruker 300 MHz and 75 MHz digital FT-NMR spectrometer (Bruker Bioscience, Billerica, MA, USA) in DMSO-*d_6_*, respectively. Splitting patterns were designated as follows: s: singlet; d: doublet; t: triplet; m: multiplet in the NMR spectra. Coupling constants (*J*) were reported as Hertz. M+1 peaks were determined by Shimadzu LC/MS ITTOF system (Shimadzu, Tokyo, Japan). All reactions were monitored by thin-layer chromatography (TLC) using Silica Gel 60 F254 TLC plates (Merck KGaA, Darmstadt, Germany).

#### 3.1.1. Synthesis of 4-(5-substitüe-1H-benz[d]imidazol-2-yl)benzoic Acid Methyl Ester (**1a**,**1b**)

Yields: 82–78%. 4-(5-substitüe-1*H*-benz[*d*]imidazol-2-yl)benzoic acid hydrazide derivatives (**2a**,**2b**) (yields: 76–72%) and 5-[4-(5(6)-substituted-1*H*-benz[*d*]imidazol-2-yl)phenyl]-1,3,4-oxadiazole-2-thiol derivatives (**3a**,**3b**) (yields: 78–7 %) were prepared following the reported procedures [23].

#### 3.1.2. 4-Amino-5-(4-(5-substitüe-1H-benzo[d]imidazol-2-yl)phenyl)-4H-1,2,4-triazole-3-thiol (**4a**,**4b**)

Compound **3a** or **3b** (0.02 mol) was dissolved in ethanol. Hydrazine hydrate (5 mL) was added to the mixture. The reaction mixture was heated at 240 °C and 10 bar for 10 min under microwave synthesis reactor (Anton-Paar Monowave 300). After the reaction ended, the product was washed with water, dried, and crystallized using ethanol (96%) [22]. Yields: 68–64%.

#### 3.1.3. 3-(4-(5-substitüe-1H-benzo[d]imidazol-2-yl)phenyl)-6-(substitüephenyl)-7H-[1,2,4]triazole [3,4-b][1,3,4]thiadiazine (**5a**–**5p**)

Compound **4a** or **4b** (0.003 mol) and appropriate phenacyl bromide (0.003 mol) in ethanol was heated under reflux for 8 h. After the reaction ended, the product was filtered and dried [22].

3-(4-(1H-benzo[d]imidazol-2-yl)phenyl)-6-phenyl-7H-[1,2,4]triazole[3,4-b][1,3,4] thiadiazine (5a): Yields: 77%. Mp 230.7–232.9 °C. ^1^H-NMR (300 MHz, DMSO-*d_6_*): δ = 4.52 (2H, s, -CH_2_-), 7.52-7.55 (2H, m, aromatic CH), 7.59–7.67 (3H, m, Aromatic CH), 7.83–7.86 (2H, m, aromatic CH), 8.06–8.09 (2H, m, aromatic CH), 8.34 (2H, d, *J*=8.67 Hz, 1,4-disubstitutedbenzene), 8.43 (2H, d, *J*=8.64 Hz, 1,4-disubstitutedbenzene). ^13^C-NMR (75 MHz, DMSO-d6): δ = 23.31, 114.89, 125.84, 126.55, 128.13, 128.50, 129.13, 129.65, 132.64, 133.78, 134.17, 143.96, 149.21, 151.22, 156.82, 163.07. HRMS (*m*/*z*): [M + H]^+^ calcd for C_23_H_16_N_6_S: 409.1224; found: 409.1230.

3-(4-(1H-benzo[d]imidazol-2-yl)phenyl)-6-(4-chlorophenyl)-7H-[1,2,4]triazole[3,4-b][1,3,4] thiadiazine (**5b**): Yields: 72%. Mp 190.9–192.6 °C. ^1^H-NMR (300 MHz, DMSO-*d_6_*): δ = 4.50 (2H, s, -CH_2_-), 7.56–7.59 (2H, m, aromatic CH), 7.65–7.70 (3H, m, aromatic CH), 7.86–7.89 (2H, m, aromatic CH), 8.06–8.09 (2H, m, aromatic CH), 8.31 (2H, d, *J*=8.61 Hz, 1,4-disubstitutedbenzene), 8.57 (2H, d, *J*=8.61 Hz, 1,4- Disubstitutedbenzene). ^13^C-NMR (75 MHz, DMSO-*d_6_*): δ=23.27, 114.67, 125.49, 126.35, 128.87, 129.11, 129.32, 129.45, 129.73, 129.92, 130.11, 130.71, 132.62, 133.01, 137.50, 144.01, 148.52, 151.17, 156.87. HRMS (*m*/*z*): [M + H]^+^ calcd for C_23_H_15_N_6_SCl: 443.0843; found: 443.0840.

3-(4-(1*H*-benzo[*d*]imidazol-2-yl)phenyl)-6-(4-fluorophenyl)-7H-[1,2,4]triazole[3,4-b][1,3,4] thiadiazine (**5c**): Yields: 69%. Mp 274.2–277.1 °C. ^1^H-NMR (300 MHz, DMSO-*d_6_*): δ = 4.50 (2H, s, -CH_2_-), 7.12 (1H, s, aromatic CH), 7.29 (1H, s, aromatic CH), 7.46–7.52 (4H, m, aromatic CH), 7.81–7.84 (2H, m, aromatic CH), 8.33 (2H, d, *J* = 8.64 Hz, 1,4-disubstitutedbenzene), 8.51 (2H, d, J=8.76 Hz, 1,4-disubstitutedbenzene). ^13^C-NMR (75 MHz, DMSO-*d_6_*): δ = 23.27, 114.32, 114.90, 116.76 (d, *J =* 21.98 Hz), 126.40, 127.68, 127.96, 128.43, 128.56, 128.69, 128.87, 128.95, 130.32 (d, *J* = 2.77 Hz), 130.78 (d, *J =* 8.43 Hz), 134.60, 143.66, 151.30, 155.71, 166.36 (d, *J* = 249.53 Hz). HRMS (*m*/*z*): [M + H]^+^ calcd for C_23_H_15_N_6_FS: 427.1137; found: 427.1136.

3-(4-(1*H*-benzo[*d*]imidazol-2-yl)phenyl)-6-(4-methylphenyl)-7H-[1,2,4]triazole[3,4-b][1,3,4] thiadiazine (**5d**): Yields: 70%. Mp 207.5–209.1 °C. ^1^H-NMR (300 MHz, DMSO-*d_6_*): δ = 2.41 (3H, s, CH_3_), 4.49 (2H, s, -CH_2_-), 7.41 (2H, d, *J* = 8.13 Hz, 1,4-disubstitutedbenzene), 7.52–7.55 (2H, m, benzimidazole CH), 7.83–7.86 (2H, m, benzimidazole CH), 7.97 (2H, d, *J* = 8.25 Hz, 1,4-disubstitutedbenzene), 8.33 (2H, d, *J* = 8.58 Hz, 1,4-disubstitutedbenzene), 8.43 (2H, d, *J* = 8.61 Hz, 1,4-disubstitutedbenzene). ^13^C-NMR (75 MHz, DMSO-*d_6_*): δ=21.55, 23.18, 114.86, 125.84, 126.42, 128.08, 128.24, 128.47, 129.02, 129.22, 129.71, 130.20, 130.90, 134.11, 142.96, 144.00, 147.35, 149.13, 151.07, 156.69. HRMS (*m*/*z*): [M + H]^+^ calcd for C_24_H_18_N_6_S: 423.1381; found: 423.1386.

3-(4-(1*H*-benzo[*d*]imidazol-2-yl)phenyl)-6-(4-cyanophenyl)-7H-[1,2,4]triazole[3,4-b][1,3,4] thiadiazine (**5e**): Yields: 68%. Mp 259.3–263.1 °C. ^1^H-NMR (300 MHz, DMSO-*d_6_*): δ = 4.53 (2H, s, -CH_2_-), 7.56–7.58 (2H, m, benzimidazole CH), 7.85–7.88 (2H, m, benzimidazole CH), 8.06 (2H, d, *J* = 8.37 Hz, 1,4-disubstitutedbenzene), 8.21 (2H, d, *J* = 8.46 Hz, 1,4-disubstitutedbenzene), 8.30 (2H, d, *J* = 8.49 Hz, 1,4-disubstitutedbenzene), 8.42 (2H, d, *J* = 8.49 Hz, 1,4-disubstitutedbenzene). ^13^C-NMR (75 MHz, DMSO-*d_6_*): δ = 23.35, 114.58, 114.71, 118.68, 125.51, 126.39, 127.20, 128.48, 128.74, 128.87, 129.19, 129.43, 129.84, 133.03, 133.44, 137.97, 143.97, 148.72, 151.25, 155.45. HRMS (*m*/*z*): [M + H]^+^ calcd for C_24_H_15_N_7_S: 434.1186; found: 434.1182.

3-(4-(1*H*-benzo[*d*]imidazol-2-yl)phenyl)-6-(4-bromophenyl)-7H-[1,2,4]triazole[3,4-b][1,3,4] thiadiazine (**5f**): Yields: 74%. Mp 284.8–286.2 °C. ^1^H-NMR (300 MHz, DMSO-*d_6_*): δ = 4.50 (2H, s, -CH_2_-), 7.53–7.56 (3H, m, aromatic CH), 7.81–7.85 (3H, m, aromatic CH), 7.99–8.02 (2H, m, aromatic CH), 8.32 (2H, d, *J* = 8.64 Hz, 1,4-disubstitutedbenzene), 8.42 (2H, d, *J* = 8.70 Hz, 1,4-disubstitutedbenzene). ^13^C-NMR (75 MHz, DMSO-*d_6_*): δ = 23.20, 114.91, 125.04, 125.96, 126.50, 127.03, 128.56, 129.19, 130.09, 130.90, 132.37, 132.67, 133.00, 142.94, 143.96, 147.34, 149.17, 151.22, 155.95. HRMS (*m*/*z*): [M + H]^+^ calcd for C_23_H_15_N_6_SBr: 487.0311; found: 487.0335.

3-(4-(1H-benzo[d]imidazol-2-yl)phenyl)-6-(2,4-difluorohenyl)-7H-[1,2,4]triazole [3,4-b][1,3,4] thiadiazine (**5g**): Yields: 72%. Mp 208.5–210.7 °C. ^1^H-NMR (300 MHz, DMSO-*d_6_*): δ = 4.42 (2H, s, -CH_2_-), 7.46–7.49 (4H, m, aromatic CH), 7.79–7.82 (3H, m, aromatic CH), 7.29–7.35 (2H, m, aromatic CH), 8.45–8.51 (2H, m, aromatic CH). ^13^C-NMR (75 MHz, DMSO-*d_6_*): δ = 25.37, 113.01 (dd, *J_1_* = 3.08 Hz, *J_2_* = 21.45 Hz), 106.08 (d, *J* = 26.01 Hz), 114.05, 114.45, 119.72 (dd, *J_1_* = 3.02 Hz, *J_2_* = 11.51 Hz), 126.56, 127.17, 128.25, 128.75, 129.15, 132.37, 132.67 (dd, *J_1_* = 3.12 Hz, *J_2_* = 10.36 Hz), 134.23, 135.55, 143.74, 148.22, 151.06, 153.70, 164.66 (d, *J* = 251.18 Hz), 164.83 (d, *J* = 250.91 Hz). HRMS (*m*/*z*): [M + H]^+^ calcd for C_23_H_14_N_6_SF_2_: 445.1053; found: 445.1041.

3-(4-(1*H*-benzo[*d*]imidazol-2-yl)phenyl)-6-(2,4-dichlorophenyl)-7H-[1,2,4]triazole[3,4-b] [1,3,4] thiadiazine (**5h**): Yields: 77%. Mp 190.7–193.4 °C. ^1^H-NMR (300 MHz, DMSO-*d_6_*): δ = 4.36 (2H, s, -CH_2_-), 7.34–7.37 (3H, m, aromatic CH), 7.65 (1H, dd, *J_1_* = 2.04 Hz, *J_2_* = 8.34 Hz, aromatic CH), 7.70–7.73 (2H, m, aromatic CH), 7.78–7.81 (1H, m, aromatic CH), 8.20 (2H, d, *J* = 8.64 Hz, 1,4-disubstitutedbenzene), 8.40 (2H, d, *J* = 8.61 Hz, 1,4-disubstitutedbenzene). ^13^C-NMR (75 MHz, DMSO-*d_6_*): δ = 26.23, 115.05, 115.32, 124.22, 124.72, 126.99, 127.78, 128.08, 128.29, 128.57, 128.96, 129.59, 130.38, 132.93, 133.05, 133.74, 136.86, 143.65, 149.92, 151.62, 156.53. HRMS (*m*/*z*): [M + H]^+^ calcd for C_23_H_14_N_6_SCl_2_: 477.0457; found: 477.0450.

3-(4-(5-chloro-1*H*-benzo[*d*]imidazol-2-yl)phenyl)-6-phenyl-7H-[1,2,4]triazole[3,4-b][1,3,4] thiadiazine (**5i**): Yields: 80%. Mp: 247.3–249.6 °C. ^1^H-NMR (300 MHz, DMSO-*d_6_*): δ = 4.50 (2H, s, -CH_2_-), 7.49 (1H, dd, *J_1_* = 1.89 Hz, *J_2_*=8.67 Hz, aromatic CH), 7.59–7.62 (3H, m, aromatic CH), 7.78–7.84 (2H, m, aromatic CH), 8.03–8.06 (2H, m, aromatic CH), 8.27 (2H, d, *J* = 8.64 Hz, 1,4-disubstitutedbenzene), 8.37 (2H, d, *J* = 8.64 Hz, 1,4-disubstitutedbenzene). ^13^C-NMR (75 MHz, DMSO-*d_6_*): δ = 23.32, 114.60, 116.29, 125.95, 126.40, 128.11, 128.47, 128.97, 129.20, 129.63, 129.94, 132.62, 133.27, 133.71, 135.39, 143.97, 150.42, 151.04, 156.76. HRMS (*m*/*z*): [M + H]^+^ calcd for C_23_H_15_N_6_SCl: 443.0826; found: 443.0840.

3-(4-(5-chloro-1*H*-benzo[*d*]imidazol-2-yl)phenyl)-6-(4-chlorophenyl)-7H-[1,2,4]triazole [3,4-b][1,3,4] thiadiazine (**5j**): Yields: 70%. Mp: 226.0–228.9 °C. ^1^H-NMR (300 MHz, DMSO-*d_6_*): δ = 4.46 (2H, s, -CH_2_-), 7.33–7.37 (1H, m, benzimidazole CH), 7.63 (2H, d, *J* = 8.61 Hz, 1,4-disubstitutedbenzene), 7.68–7.73 (2H, m, benzimidazole CH), 8.04 (2H, d, *J* = 8.61 Hz, 1,4-disubstitutedbenzene), 8.19 (2H, d, *J* = 8.46 Hz, 1,4-disubstitutedbenzene), 8.39 (2H, d, *J* = 8.43 Hz, 1,4-disubstitutedbenzene). ^13^C-NMR (75 MHz, DMSO-*d_6_*): δ = 23.13, 115.14, 116.51, 124.18, 127.79, 128.20, 128.33, 128.83, 128.98, 129.19, 129.68, 129.87, 130.51, 131.57, 132.60, 137.42, 143.58, 151.40, 155.59. HRMS (*m*/*z*): [M + H]^+^ calcd for C_23_H_14_N_6_SCl_2_: 477.0427; found: 477.0450.

3-(4-(5-chloro-1*H*-benzo[*d*]imidazol-2-yl)phenyl)-6-(4-fluorophenyl)-7H-[1,2,4]triazole [3,4-b][1,3,4] thiadiazine (**5k**): Yields: 71%. Mp: 249.1–250.9 °C. ^1^H-NMR (300 MHz, DMSO-*d_6_*): δ = 4.48 (2H, s, -CH_2_-), 7.38 (1H, dd, *J_1_* = 1.98 Hz, *J_2_* = 8.58 Hz, aromatic CH), 7.46–7.48 (2H, m, aromatic CH), 7.71–7.74 (1H, m, aromatic CH), 7.77 (1H, s, aromatic CH), 8.12–8.14 (2H, m, aromatic CH), 8.25 (2H, d, *J* = 8.64 Hz, 1,4-disubstitutedbenzene), 8.40 (2H, d, *J* = 8.61 Hz, 1,4-disubstitutedbenzene). ^13^C-NMR (75 MHz, DMSO-*d_6_*): δ = 23.45, 114.95, 116.45, 116.77 (d, *J* = 22.09 Hz), 124.83, 128.07, 128.21, 128.39, 128.84, 128.89, 130.30 (d, *J* = 2.99 Hz), 130.85 (d, *J* = 8.93 Hz), 135.26, 137.58, 143.69, 150.99, 151.27, 155.73, 164.82 (d, *J* = 249.42 Hz). HRMS (*m*/*z*): [M + H]^+^ calcd for C_23_H_14_N_6_FSCl: 461.0741; found: 461.0746.

3-(4-(5-chloro-1*H*-benzo[*d*]imidazol-2-yl)phenyl)-6-(4-methylphenyl)-7H-[1,2,4]triazole [3,4-b][1,3,4] thiadiazine (**5l**): Yields: 65%. Mp: 250.7–252.3 °C. ^1^H-NMR (300 MHz, DMSO-*d_6_*): δ = 4.46 (2H, s, -CH2-), 7.40 (2H, d, *J* = 8.07 Hz, 1,4-disubstitutedbenzene), 7.45 (1H, dd, *J_1_* = 1.95 Hz, *J_2_* = 8.67 Hz, benzimidazole CH), 7.76–7.79 (1H, m, benzimidazole CH), 7.82–7.83 (1H, m, benzimidazole CH), 7.95 (2H, d, *J* = 8.31 Hz, 1,4-disubstitutedbenzene), 8.28 (2H, d, *J* = 8.67 Hz, 1,4-disubstitutedbenzene), 8.37 (2H, d, *J* = 8.67 Hz, 1,4-disubstitutedbenzene). ^13^C-NMR (75 MHz, DMSO-*d_6_*): δ = 21.54, 23.15, 114.88, 116.44, 125.26, 127.74, 128.08, 128.21, 128.95, 129.24, 129.72, 130.21, 130.90, 134.62, 136.89, 142.95, 143.90, 150.99, 151.15, 156.66. HRMS (*m*/*z*): [M + H]^+^ calcd for C_24_H_17_N_6_SCl: 457.0994; found: 457.0997.

3-(4-(5-chloro-1*H*-benzo[*d*]imidazol-2-yl)phenyl)-6-(4-cyanophenyl)-7H-[1,2,4]triazole [3,4-b][1,3,4] thiadiazine (**5m**): Yields: 62%. Mp: 254.0–255.7 °C. ^1^H-NMR (300 MHz, DMSO-*d_6_*): δ = 4.53 (2H, s, -CH_2_-), 7.50 (1H, dd, *J_1_* = 1.95 Hz, *J_2_* = 8.70 Hz, benzimidazole CH), 7.85–7.86 (1H, m, aromatic CH), 8.06–8.08 (3H, m, aromatic CH), 8.21 (2H, d, *J* = 8.61 Hz, 1,4-disubstitutedbenzene), 8.26 (2H, d, *J* = 8.64 Hz, 1,4-disubstitutedbenzene), 8.38 (2H, d, *J* = 8.64 Hz, 1,4-disubstitutedbenzene). ^13^C-NMR (75 MHz, DMSO-*d_6_*): δ = 23.30, 114.57, 114.71, 118.70, 125.83, 126.85, 128.48, 128.86, 129.11, 129.37, 129.81, 133.46, 133.58, 135.73, 137.98, 143.88, 150.56, 151.33, 155.41. HRMS (*m*/*z*): [M + H]^+^ calcd for C_24_H_14_N_7_SCl: 468.0782; found: 468.0793.

3-(4-(5-chloro-1*H*-benzo[*d*]imidazol-2-yl)phenyl)-6-(4-bromophenyl)-7H-[1,2,4]triazole [3,4-b][1,3,4] thiadiazine (**5n**): Yields: 76%. Mp: 243.4–247.3 °C. ^1^H-NMR (300 MHz, DMSO-*d_6_*): δ = 4.48 (2H, s, -CH_2_-), 7.43 (1H, dd, *J_1_* = 1.98 Hz, *J_2_* = 8.67 Hz, benzimidazole CH), 7.75 (1H, s, aromatic CH), 7.80–7.82 (3H, m, aromatic CH), 7.99 (2H, d, *J* = 8.70 Hz, 1,4-disubstitutedbenzene), 8.26 (2H, d, *J* = 8.58 Hz, 1,4-disubstitutedbenzene), 8.37 (2H, d, *J* = 8.64 Hz, 1,4-disubstitutedbenzene). ^13^C-NMR (75 MHz, DMSO-*d_6_*): δ=23.14, 115.01, 116.52, 124.92, 126.45, 128.10, 129.05, 130.07, 131.76, 132.00, 132.15, 132.67, 133.00, 141.45, 143.77, 148.89, 149.69, 151.39, 155.91. HRMS (*m*/*z*): [M + H]^+^ calcd for C_23_H_14_N_6_SClBr: 520.9930; found: 520.9945.

3-(4-(5-chloro-1*H*-benzo[*d*]imidazol-2-yl)phenyl)-6-(2,4-difluorohenyl)-7H-[1,2,4]triazole [3,4-b][1,3,4] thiadiazine (**5o**): Yields: 66%. Mp: 182.2–185.0 °C. ^1^H-NMR (300 MHz, DMSO-*d_6_*): δ = 4.39 (2H, s, -CH_2_-), 7.28–7.34 (1H, m, aromatic CH), 7.42–7.48 (1H, m, aromatic CH), 7.48–7.52 (2H, m, aromatic CH), 7.77–7.83 (2H, m, aromatic CH), 7.93–8.01 (1H, m, aromatic CH), 8.25 (2H, d, *J* = 8.64 Hz, 1,4-disubstitutedbenzene), 8.44 (2H, d, *J* = 8.58 Hz, 1,4-disubstitutedbenzene). ^13^C-NMR (75 MHz, DMSO-*d_6_*): δ = 25.31, 105.76 (d, *J* = 26.01 Hz), 106.28, 113.19 (dd, *J_1_* = 2.63 Hz, *J_2_* = 20.86 Hz), 114.74, 116.39, 119.76 (dd, *J*_1_ = 3.73 Hz, *J_2_* = 7.83 Hz), 125.45, 127.49, 128.32, 128.94, 129.14, 129.40, 132.71 (dd, *J_1_* = 3.21 Hz, *J_2_* = 9.80 Hz), 134.22, 143.80, 151.60, 151.26, 153.78, 161.28 (d, *J* = 252.62 Hz), 164.46 (d, *J* = 256.22 Hz). HRMS (*m*/*z*): [M + H]^+^ calcd for C_23_H_13_N_6_F_2_SCl: 479.0632; found: 479.0652.

3-(4-(5-chloro-1*H*-benzo[*d*]imidazol-2-yl)phenyl)-6-(2,4-dichlorophenyl)-7H-[1,2,4]triazole [3,4-b][1,3,4] thiadiazine (**5p**): Yields: 68%. Mp: 114.4–116.5 °C. ^1^H-NMR (300 MHz, DMSO-*d_6_*): δ = 4.35 (2H, s, -CH_2_-), 7.25 (1H, dd, *J_1_* = 1.74 Hz, *J_2_* = 8.52 Hz, aromatic CH), 7.65 (2H, dd, *J_1_* = 2.07 Hz, *J_2_* = 8.34 Hz, aromatic CH), 7.88 (2H, d, *J* = 8.34 Hz, aromatic CH), 7.90 (1H, m, aromatic CH), 8.16 (2H, d, *J* = 8.61 Hz, 1,4-disubstitutedbenzene), 8.32 (2H, d, *J* = 8.58 Hz, 1,4-disubstitutedbenzene). ^13^C-NMR (75 MHz, DMSO-*d_6_*): δ = 26.33, 106.76, 125.55, 127.29, 127.35, 128.58, 128.90, 129.64, 130.37, 131.63, 132.35, 132.96, 133.06, 133.44, 133.77, 136.56, 136.84, 143.51, 149.25, 151.77, 156.48. HRMS (*m*/*z*): [M + H]^+^ calcd for C_23_H_13_N_6_SCl_3_: 511.0037; found: 511.0061.

### 3.2. Cytotoxicity Assay

The anticancer activity of compounds **5a**–**5p** were screened according to the MTT assays. The MTT assays were performed as previously described [24,25]. Cisplatin was used as the reference drug for the MCF7 cell line in the MTT assays.

### 3.3. Aromatase Inhibition Assay

This method was carried out according to the kit procedure (Bio Vision, Aromatase (CYP19A) Inhibitor Screening Kit (Fluorometric)). The compounds were dissolved in dimethyl sulfoxide (DMSO) and added to the assay in at least seven concentrations ranging from 10^−3^–10^−9^ M. The recombinant human aromatase stock was prepared by reconstituting with 1 mL of aromatase assay buffer. The contents were mixed thoroughly by vortexing to obtain a homogeneous solution and the solution was transferred to a 15-mL conical tube. The volume was brought to 2450 µL with the aromatase assay buffer and 50 µl of NADPH production system (100X) was added for a final total volume of 2.5 mL. Letrozole was used as a positive inhibition control. For solvent control, a small aliquot of aromatase assay buffer containing the organic solvent was used to dissolve the test compounds that were prepared. Reaction wells containing test compounds and the corresponding no inhibitor controls (which may also serve as a solvent control), as well as a background control (containing no fluorogenic Aromatase Substrate) were prepared. The plate was incubated for at least 10 min at 37 °C to allow test ligands to interact with the aromatase. After incubation, 30 µl of the aromatase substrate/NADP+ mixture was added to each well. Immediately (within 1 min), the fluorescence at Ex/Em = 488/527 nm was measured.

### 3.4. Molecular Docking

A structure-based in silico procedure was applied to discover the binding modes of compound 5e to human aromatase enzyme active site. The crystal structures of human aromatase (PDB ID: 3EQM) [26] was retrieved from the Protein Data Bank server (www.pdb.org).

The structures of ligands were built using the Schrödinger Maestro [29] interface and then were submitted to the Protein Preparation Wizard protocol of the Schrödinger Suite 2016 Update 2 [30]. The ligands were prepared by the LigPrep 3.8 [31] to assign the protonation states at pH 7.4 ± 1.0 and the atom types, correctly. Bond orders were assigned, and hydrogen atoms were added to the structures. The grid generation was formed using Glide 7.1 [32]. Flexible docking runs were performed with single precision docking mode (SP).

### 3.5. Theoretical Determination of ADME Properties

Physicochemical parameters of obtained compounds (**5a**–**5p**) were evaluated by using QikProp 4.8 [33].

## 4. Conclusions

Inhibition of aromatase has proved to be an effective approach for the treatment of hormone-dependent breast cancer in the postmenopausal women. Imidazole and triazole groups are important rings for the development new potent aromatase inhibitors with high affinity for the enzyme. A series of benzimidazole-triazolothiadiazine derivatives have been synthesized with different substituents at benzimidazole and phenyl rings. The newly synthesized compounds were tested for their anti-cancer properties against human breast cancer cell line (MCF-7). The synthesized compounds were then tested in in vitro aromatase assay and two compounds (**5e** and **5m**) exhibited activity similar to letrozole.

## Figures and Tables

**Table 1:** The synthesized compounds (**5a**–**5p**).

Comp.	R	R^1^	R^2^
**5a**	-H	-H	-H
**5b**	-H	-Cl	-H
**5c**	-H	-F	-H
**5d**	-H	-CH_3_	-H
**5e**	-H	-CN	-H
**5f**	-H	-Br	-H
**5g**	-H	-F	-F
**5h**	-H	-Cl	-Cl
**5ı**	-Cl	-H	-H
**5j**	-Cl	-Cl	-H
**5k**	-Cl	-F	-H
**5l**	-Cl	-CH_3_	-H
**5m**	-Cl	-CN	-H
**5n**	-Cl	-Br	-H
**5o**	-Cl	-F	-F
**5p**	-Cl	-Cl	-Cl

**Table 2:** IC_50_ (µM) values of compounds (**5a**–**5p**) (*n* = 4).

Comp.	MCF-7	NIH3T3
**5a**	0.142 ± 0.007	-
**5b**	0.0414 ± 0.001	-
**5c**	**0.119 ± 0.005**	**81.8 ± 2.4**
**5d**	0.194 ± 0.006	-
**5e**	**0.016 ± 0.001**	**≥1000**
**5f**	0.318 ± 0.019	-
**5g**	0.245 ± 0.004	-
**5h**	0.342 ± 0.017	-
**5ı**	0.205 ± 0.005	-
**5j**	0.258 ± 0.011	-
**5k**	**0.110 ± 0.005**	**77.6 ± 2.1**
**5l**	0.352 ± 0.019	-
**5m**	**0.018 ± 0.001**	**≥1000**
**5n**	0.264 ± 0.011	-
**5o**	0.373 ± 0.011	-
**5p**	0.147 ± 0.001	-
**Cisplatin**	**0.020 ± 0.009**	**≥1000**

**Table 3:** IC_50_ (µM) values of compounds **5c**, **5e**, **5k**, **5m,** and letrozole.

Comp.	Aromatase Inhibition
**5c**	1.716 ± 0.042
**5e**	**0.032 ± 0.001**
**5k**	2.276 ± 0.106
**5m**	1.562 ± 0.064
**Letrozole**	**0.024 ± 0.001**

**Table 4:** Calculated ADME parameters.

Comp.	MW	RB	DM	MV	DHB	AHB	PSA	logP	logS	PCaco	logBB	PMDCK	PM	CNS	%HOA	VRF	VRT
**5a**	408.48	0	13.539	1230.58	1	4.5	66.81	5.226	−7.577	1145.907	−0.361	1066.282	2	0	100	1	1
**5b**	442.925	0	11.536	1274.691	1	4.5	66.813	5.722	−8.323	1145.871	−0.207	2630.568	2	0	100	1	1
**5c**	426.47	0	11.294	1246.689	1	4.5	66.815	5.463	−7.944	1145.883	−0.255	1928.038	2	0	100	1	1
**5d**	422.506	0	14.175	1289.517	1	4.5	66.81	5.533	−8.145	1145.907	−0.388	1066.282	3	0	100	1	1
**5e**	433.489	1	7.968	1297.278	1	6	92.606	4.468	−8.534	236.976	−1.255	194.119	2	−2	95.607	0	1
**5f**	487.376	0	11.913	1283.596	1	4.5	66.813	5.799	−8.439	1145.858	−0.198	2828.358	2	0	100	1	1
**5g**	444.461	0	12.598	1251.337	1	4.5	65.595	5.544	−8.02	1167.271	−0.2	2431.401	2	0	100	1	1
**5h**	477.37	0	12.709	1287.743	1	4.5	64.6	5.897	−8.422	1199.257	−0.099	3927.141	2	0	100	1	1
**5i**	442.925	0	12.609	1274.528	1	4.5	66.806	5.721	−8.321	1145.207	−0.208	2625.13	2	0	100	1	1
**5j**	477.37	0	10.697	1318.639	1	4.5	66.809	6.217	−9.068	1145.171	−0.054	6476.318	2	0	100	1	1
**5k**	460.915	0	10.455	1290.637	1	4.5	66.811	5.957	−8.688	1145.183	−0.101	4746.726	2	0	100	1	1
**5l**	456.951	0	13.229	1333.465	1	4.5	66.806	6.028	−8.891	1145.207	−0.235	2625.13	3	0	100	1	1
**5m**	467.934	1	7.409	1341.226	1	6	92.602	4.96	−9.274	236.832	−1.117	477.911	2	−2	100	0	1
**5n**	521.821	0	11.046	1327.544	1	4.5	66.809	6.294	−9.184	1145.158	−0.044	6963.267	2	0	92.631	2	1
**5o**	478.906	0	11.99	1295.266	1	4.5	65.591	6.038	−8.764	1166.535	−0.046	5985.589	2	0	100	1	1
**5p**	511.815	0	12.056	1331.676	1	4.5	64.597	6.391	−9.166	1198.487	0.055	9667.877	2	1	93.553	2	1

**MW:** Molecular weight **RB:** Number of rotatable bonds (recommended value: 0-15) **DM:** Computed dipole moment (recommended value: 1-12.5) **MV:** Total solvent-accessible volume (recommended value: 500-2000) **DHB:** Estimated number of hydrogen bond donors (recommended value: 0-6) **AHB:** Estimated number of hydrogen bond acceptors (recommended value: 2-20) **PSA:** Van der Waals surface area of polar nitrogen and oxygen atoms and carbonyl carbon atoms (recommended value: 7-200) **logP:** Predicted octanol/water partition coefficient (recommended value: -2-6.5) **logS:** Predicted aqueous solubility (recommended value: -6.5-0.5) **PCaco:** Predicted apparent Caco-2 cell permeability (recommended value: <25 poor, >500 great) **logBB:** Predicted brain/blood partition coefficient (recommended value: -3-1.2) **PMDCK:** Predicted apparent MDCK cell permeability (recommended value: <25 poor, >500 great) **PM:** Number of likely metabolic reactions (recommended value: 1-8) **CNS:** Predicted central nervous system activity (recommended value: –2 (inactive), +2 (active)) **%HOA:** Predicted human oral absorption percent (recommended value: >80% is high, <25% is poor) **VRF:** Number of violations of Lipinski’s rule of five. The rules are: MW < 500, logP < 5, DHB ≤ 5, AHB≤ 10, Positive PSA value. **VRT:** Number of violations of Jorgensen’s rule of three. The three rules are: logS > -5.7, PCaco > 22 nm/s, PM < 7.

## Supplementary Materials

The following are available online.
