# Supplementary material for: Synthesis, Docking Studies and Biological Activity of New Benzimidazole- Triazolothiadiazine Derivatives as Aromatase Inhibitor
Source: Molecules. 2020 Apr 2;25(7):1642. doi: 10.3390/molecules25071642 (PMC7180718; doi:10.3390/molecules25071642)
Supplement: Supplementary file 1 [file molecules-25-01642-s001.pdf]

Synthesis, docking studies and biological activity of new benzimidazole- triazolothiadiazine derivatives as aromatase inhibitor

Ulviye ACAR ÇEVİK[1,2], Betül KAYA ÇAVUŞOĞLU[3], Begüm Nurpelin SAĞLIK[1,2], Derya OSMANİYE[1,2], Serkan LEVENT[1,2], Sinem ILGIN[4], Yusuf ÖZKAY*[1,2], Zafer Asım KAPLANCIKLI[1]

[1] Department of Pharmaceutical Chemistry, Faculty of Pharmacy, Anadolu Universty, Eskişehir, Turkey

[2] Doping and Narcotic Compounds Analysis Laboratory, Faculty of Pharmacy, Anadolu Universty, Eskişehir, Turkey

[3] Department of Pharmaceutical Chemistry, Faculty of Pharmacy, Bülent Ecevit Universty, Zonguldak, Turkey

[4] Department of Pharmaceutical Toxicology, Faculty of Pharmacy, Anadolu Universty, Eskişehir, Turkey

* Correspondence: yozkay@anadolu.edu.tr; Tel.: +90-222-335-0580/3603

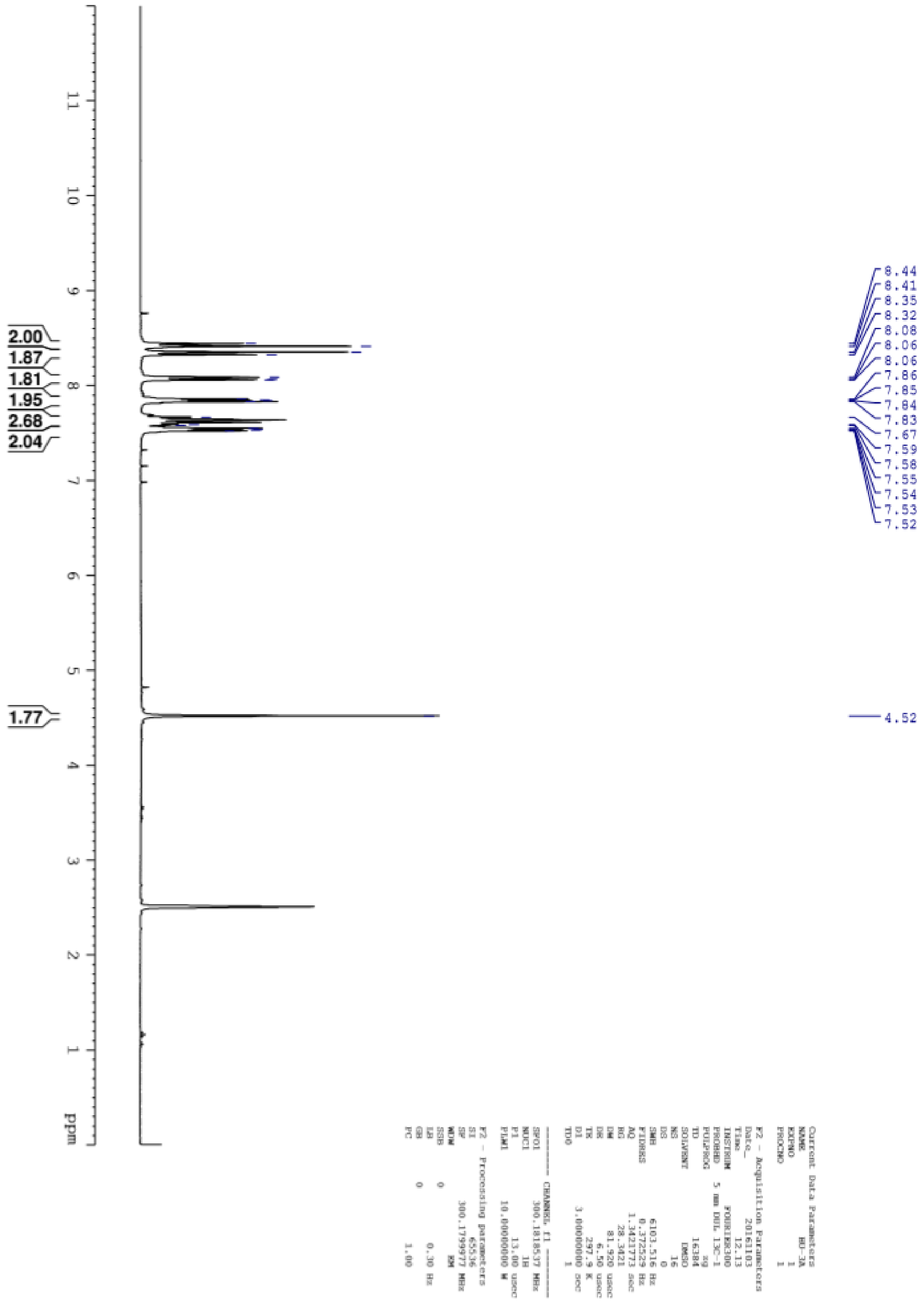

Figure S1. ^{1}H NMR spectrum of compound **5a**

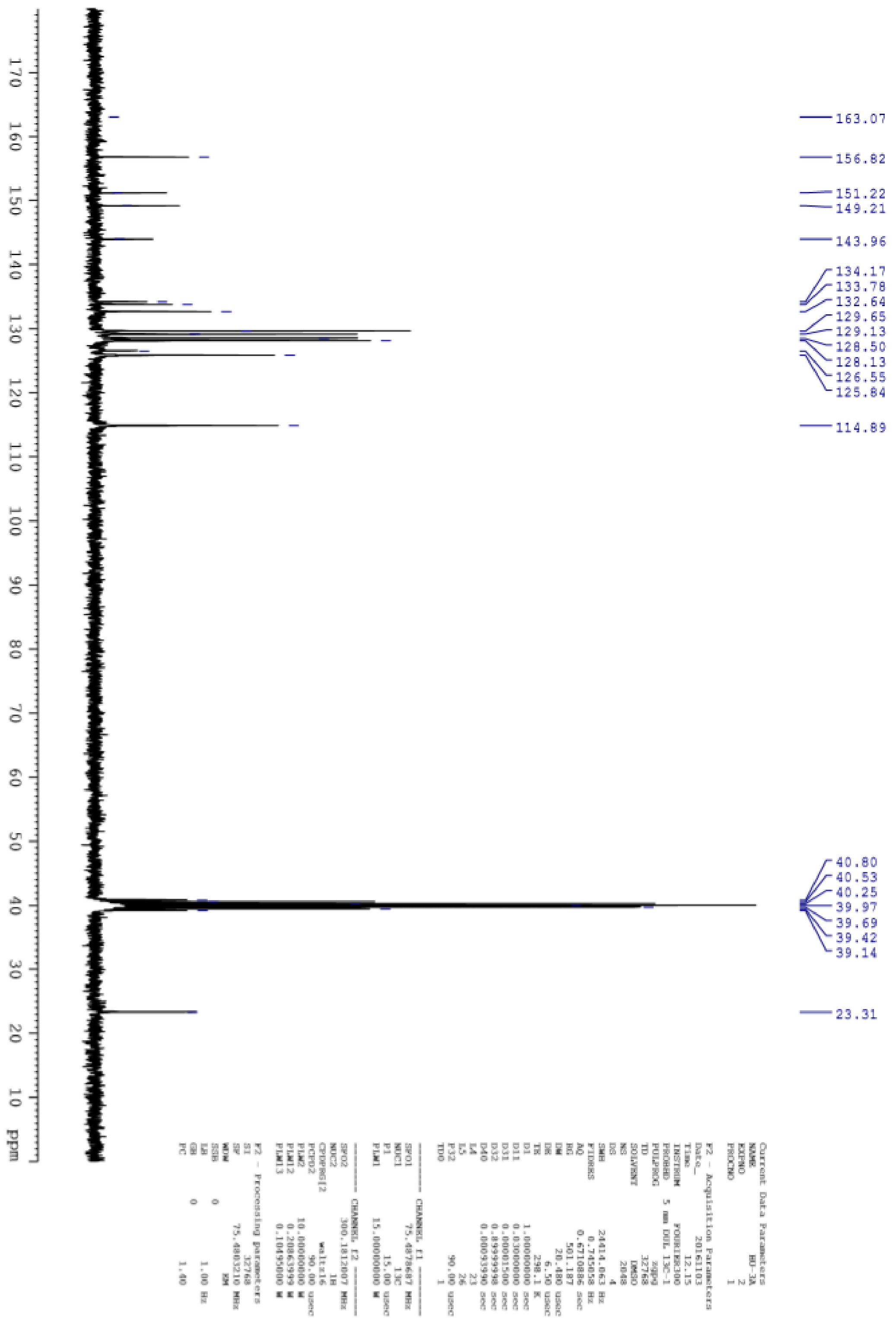

Figure S2. ^{13}C NMR spectrum of compound **5a**

Elmt	Val.	Min	Max	Elmt	Val.	Min	Max	Elmt	Val.	Min	Max	Elmt	Val.	Min	Max	Use Adduct
H	1	6	40	O	2	0	0	S	2	1	1	Ru	2	0	0	H
C	4	7	33	F	1	0	0	Cl	1	0	2	Pd	2	0	0	
N	3	0	7	P	3	0	0	Br	1	0	0	I	3	0	0	

Error Margin (ppm): 5
HC Ratio: unlimited
Max Isotopes: 3
MSn Iso RI (%): 10.00

DBE Range: 10.0 - 20.0
Apply N Rule: yes
Isotope RI (%): 1.00
MSn Logic Mode: AND

Electron Ions: both
Use MSn Info: yes
Isotope Res: 9000
Max Results: 100

Event#: 1 MS(E+) Ret. Time : 1.467 -> 1.600 Scan# : 221 -> 241

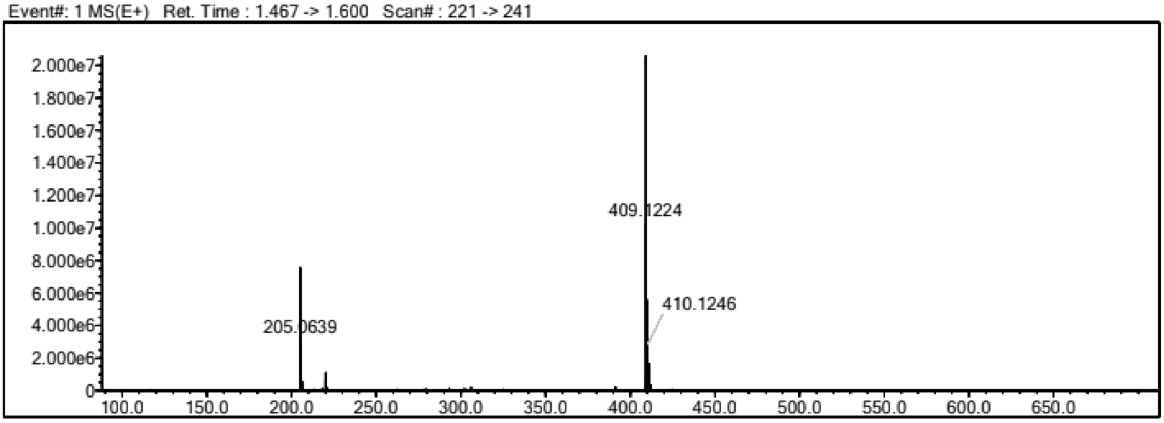

Measured region for 409.1224 m/z

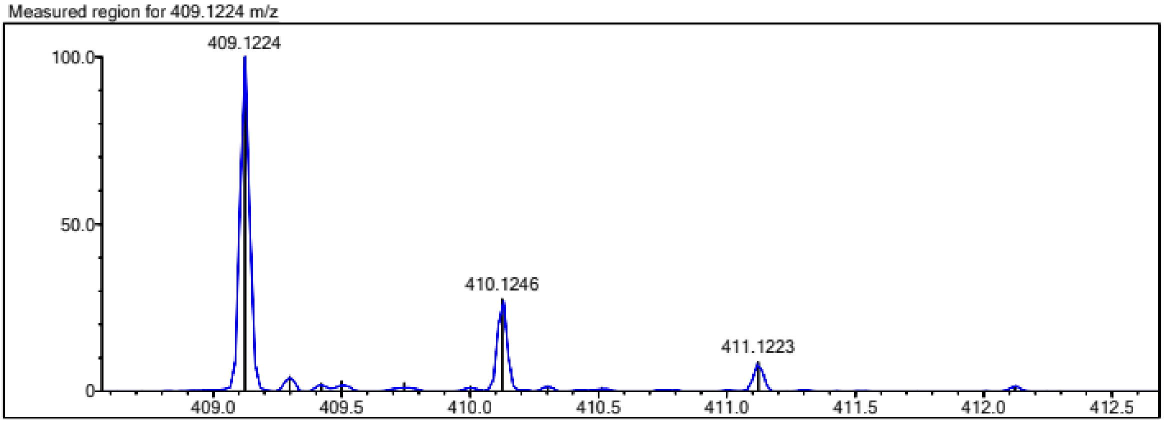

C23 H16 N6 S [M+H]+ : Predicted region for 409.1230 m/z

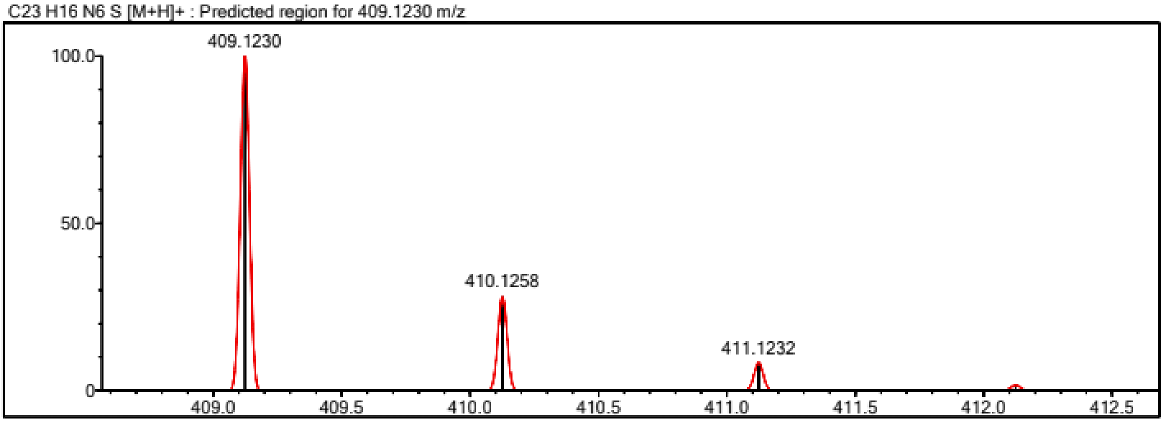

Figure S3. Mass spectrum of compound **5a**

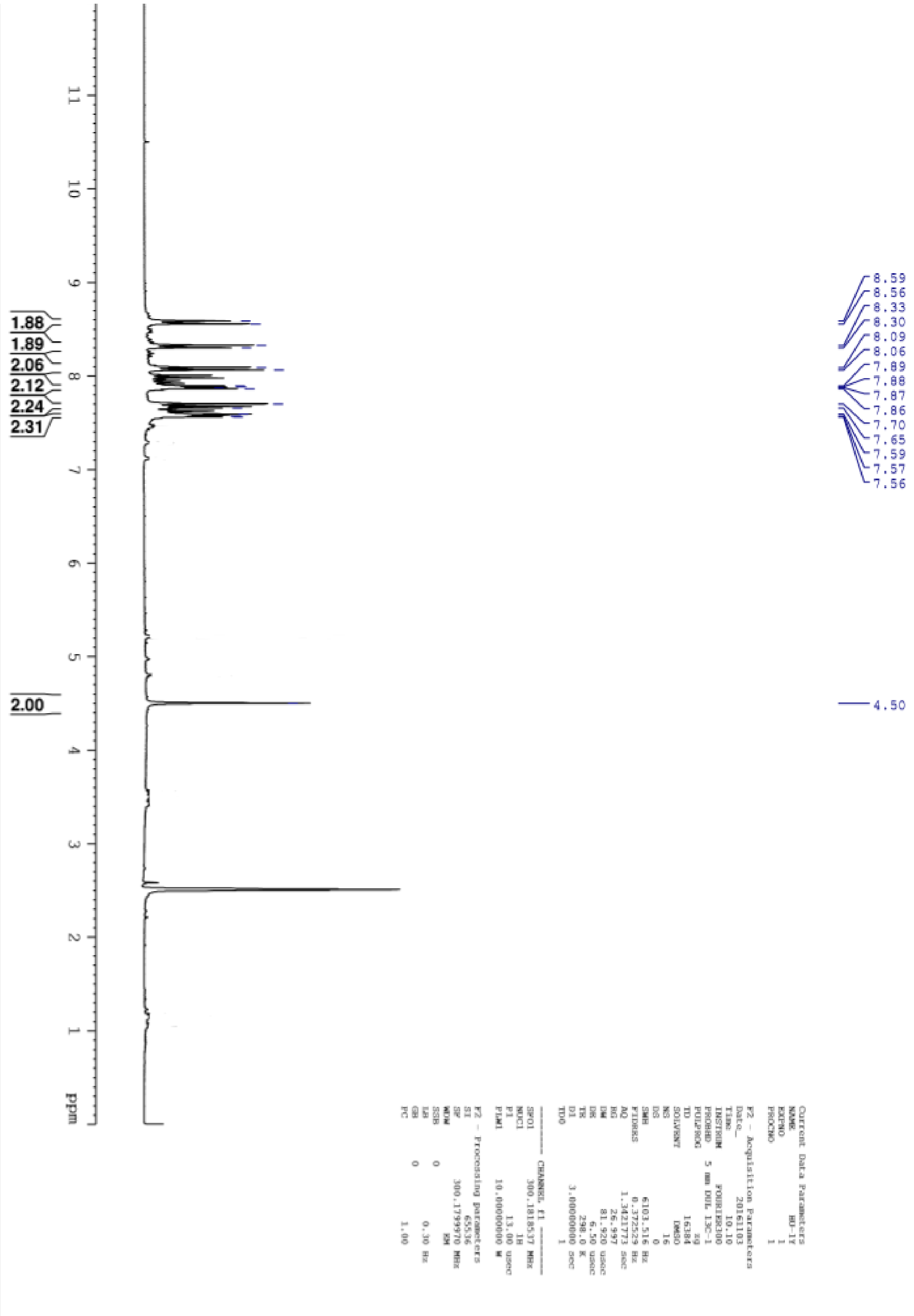

Figure S4. ^{1}H NMR spectrum of compound **5b**

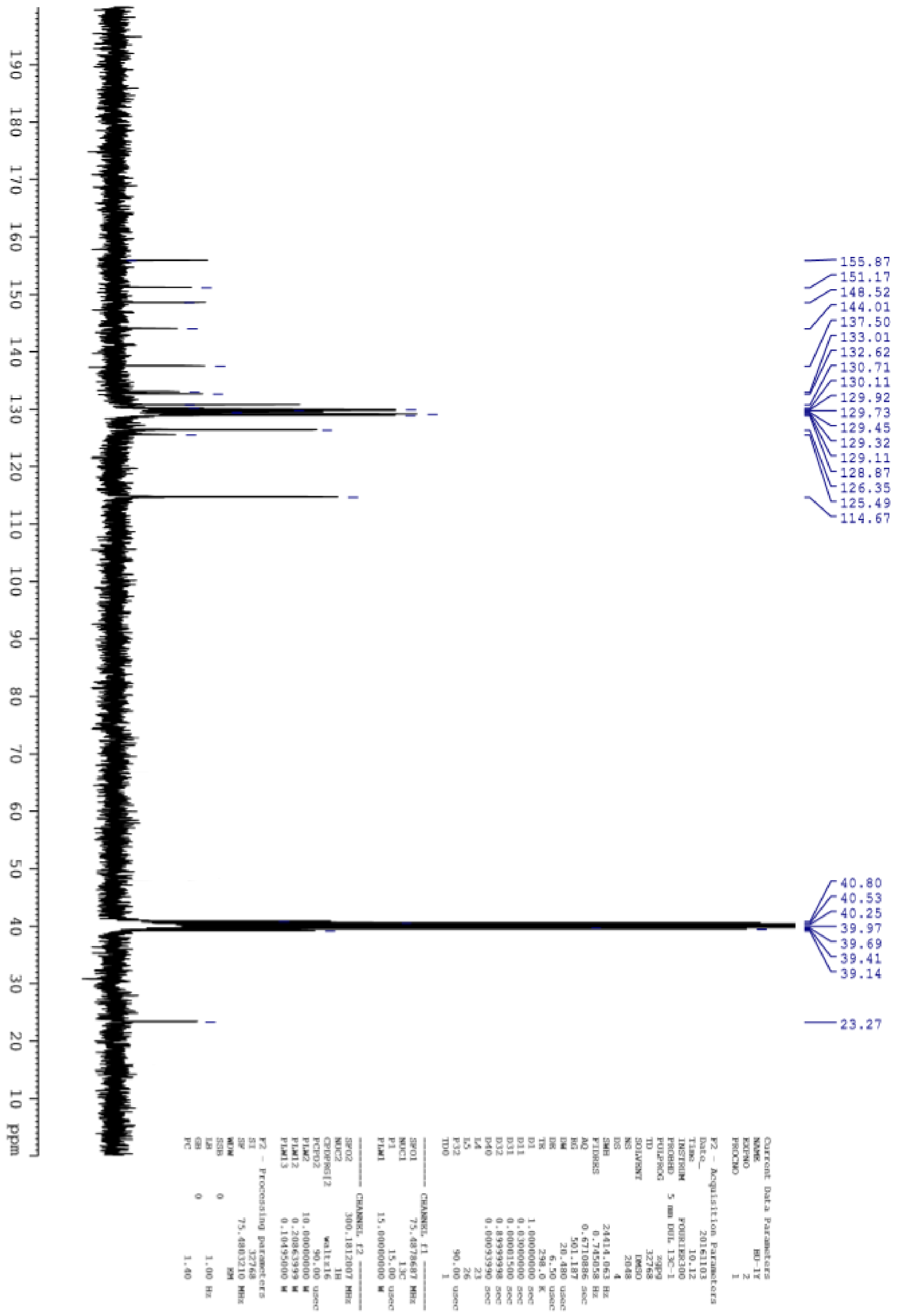

Figure S5. ^{13}C NMR spectrum of compound **5b**

Elmt	Val.	Min	Max	Elmt	Val.	Min	Max	Elmt	Val.	Min	Max	Elmt	Val.	Min	Max	Use Adduct
H	1	15	15	O	2	0	3	Cl	1	0	2	I	3	0	0	H
C	4	23	23	F	1	0	3	Br	1	0	1					
N	3	0	6	S	2	0	2	Ru	2	0	0					

Error Margin (ppm):	5	DBE Range:	10.0 - 30.0	Electron Ions:	both
HC Ratio:	unlimited	Apply N Rule:	yes	Use MSn Info:	no
Max Isotopes:	3	Isotope RI (%):	1.00	Isotope Res:	10000
MSn Iso RI (%):	10.00	MSn Logic Mode:	AND	Max Results:	500

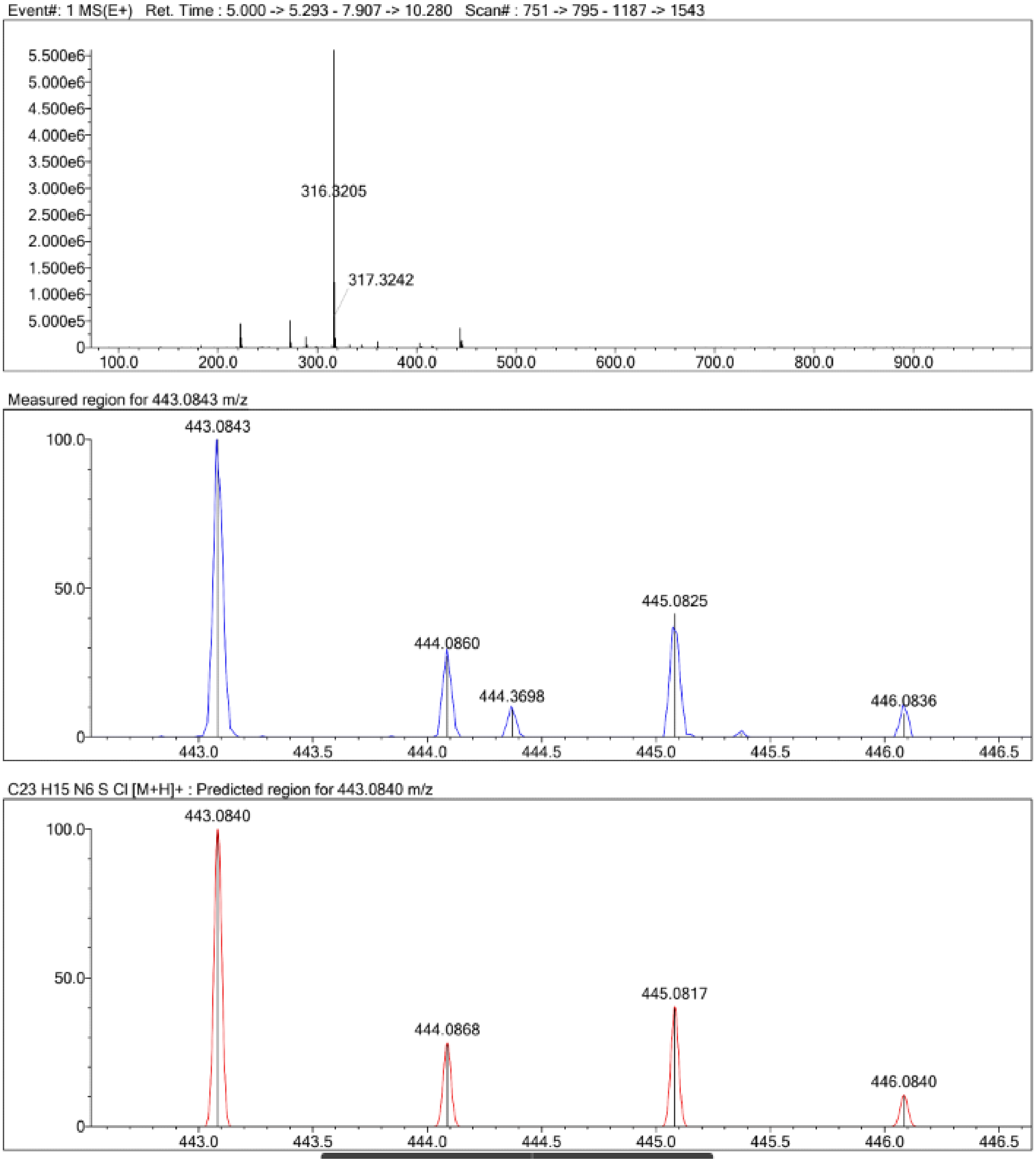

Figure S6. Mass spectrum of compound **5b**

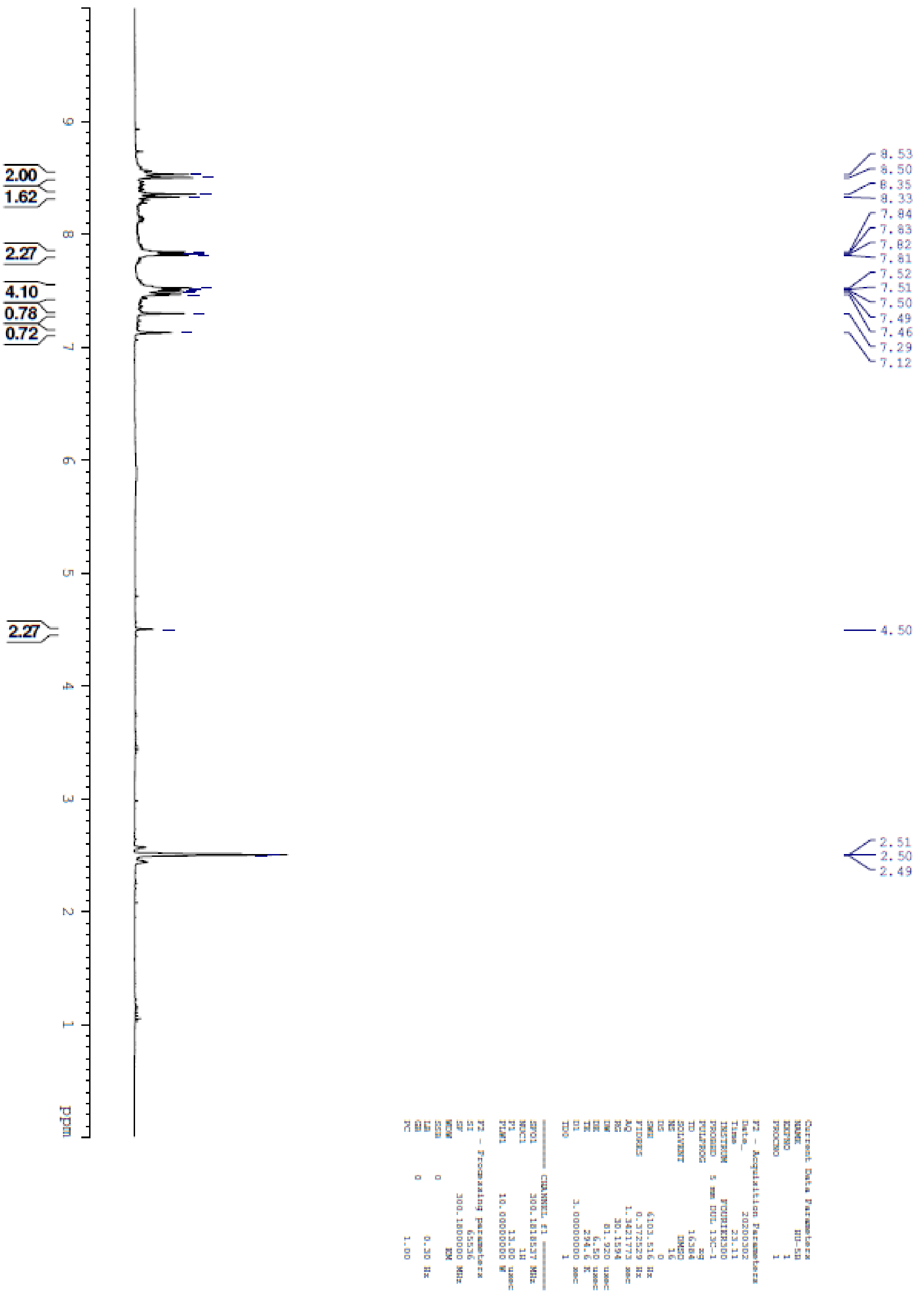

Figure S7. ^{1}H NMR spectrum of compound **5c**

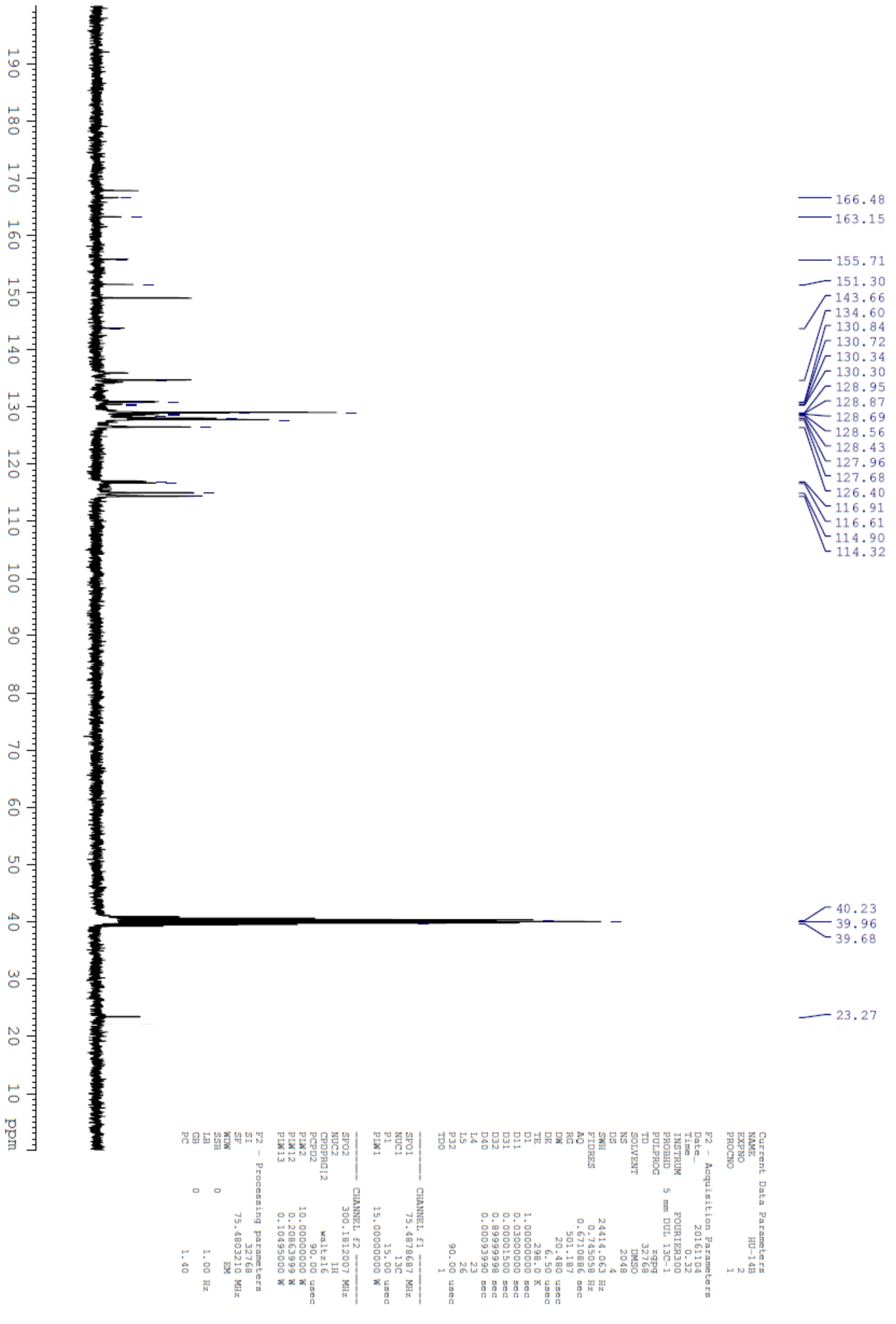

Figure S8. ^{13}C NMR spectrum of compound **5c**

Elmt	Val.	Min	Max	Elmt	Val.	Min	Max	Elmt	Val.	Min	Max	Elmt	Val.	Min	Max	Use Adduct
H	1	13	15	O	2	0	0	Cl	1	0	3	I	3	0	0	H
C	4	23	23	F	1	0	2	Br	1	0	1					
N	3	6	7	S	2	0	1	Ru	2	0	0					

Error Margin (ppm): 5
HC Ratio: unlimited
Max Isotopes: 3
MSn Iso RI (%): 10.00

DBE Range: 10.0 - 30.0
Apply N Rule: yes
Isotope RI (%): 1.00
MSn Logic Mode: AND

Electron Ions: both
Use MSn Info: no
Isotope Res: 10000
Max Results: 500

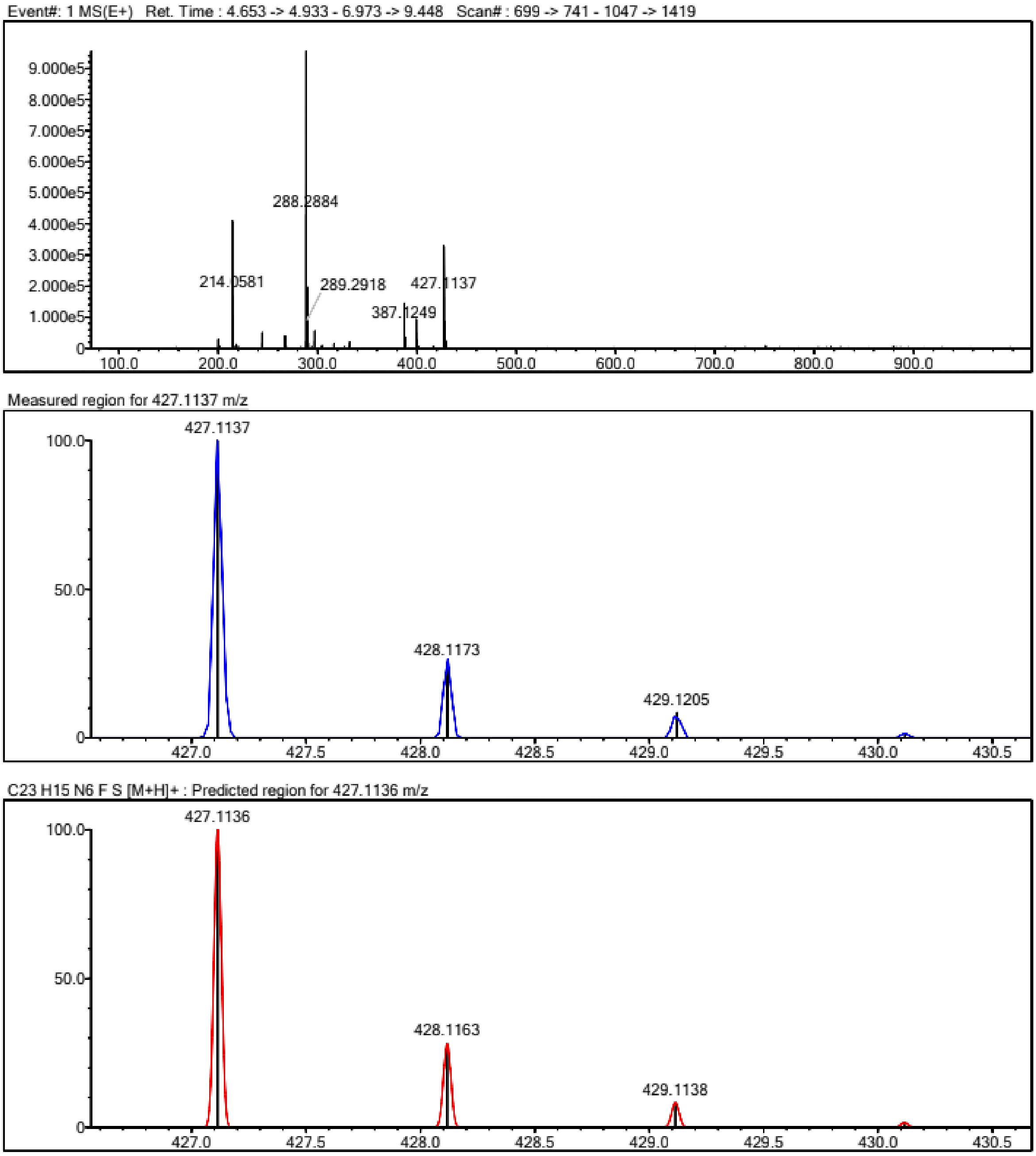

Figure S9. Mass spectrum of compound **5c**

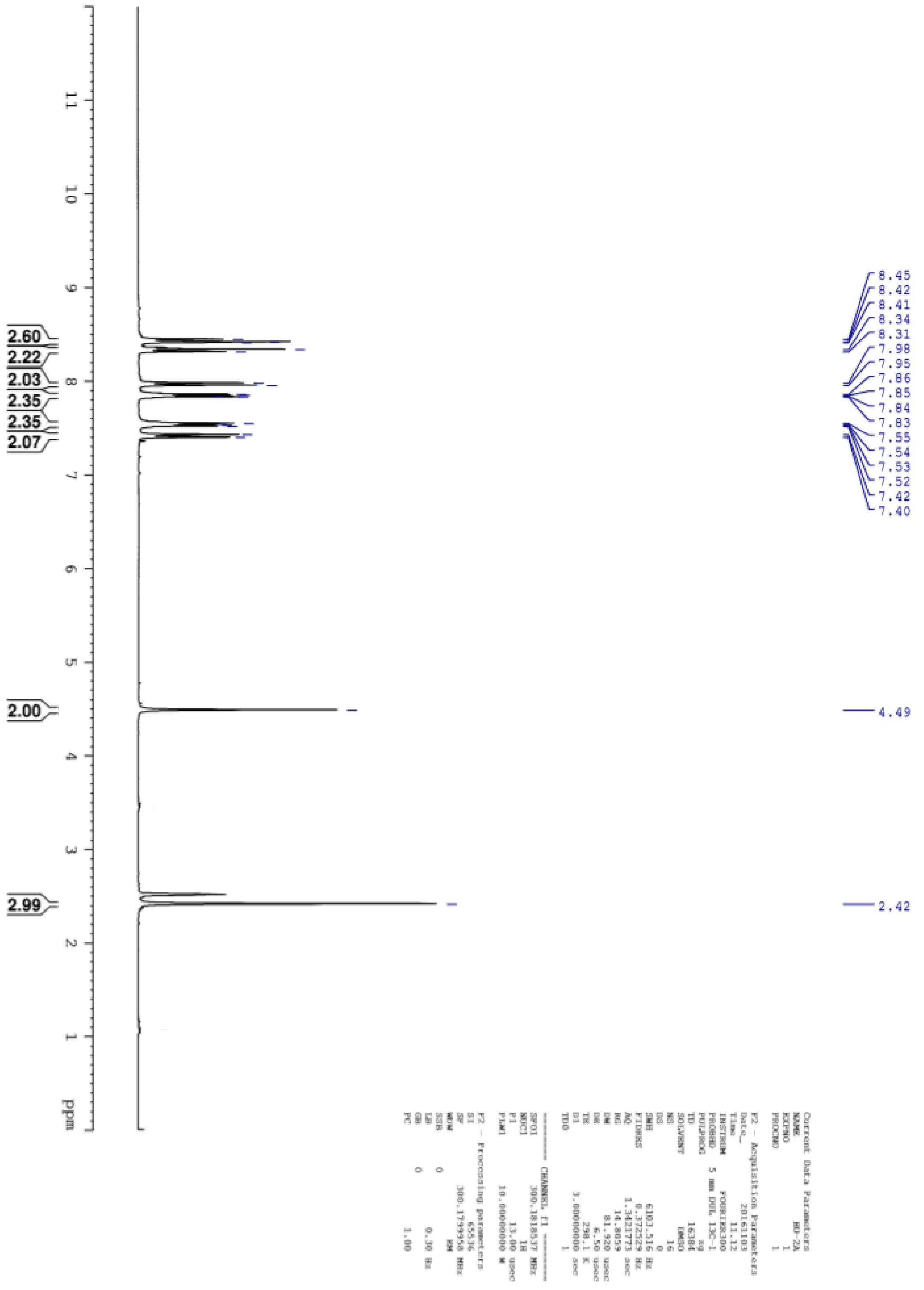

Figure S10. ^{1}H NMR spectrum of compound **5d**

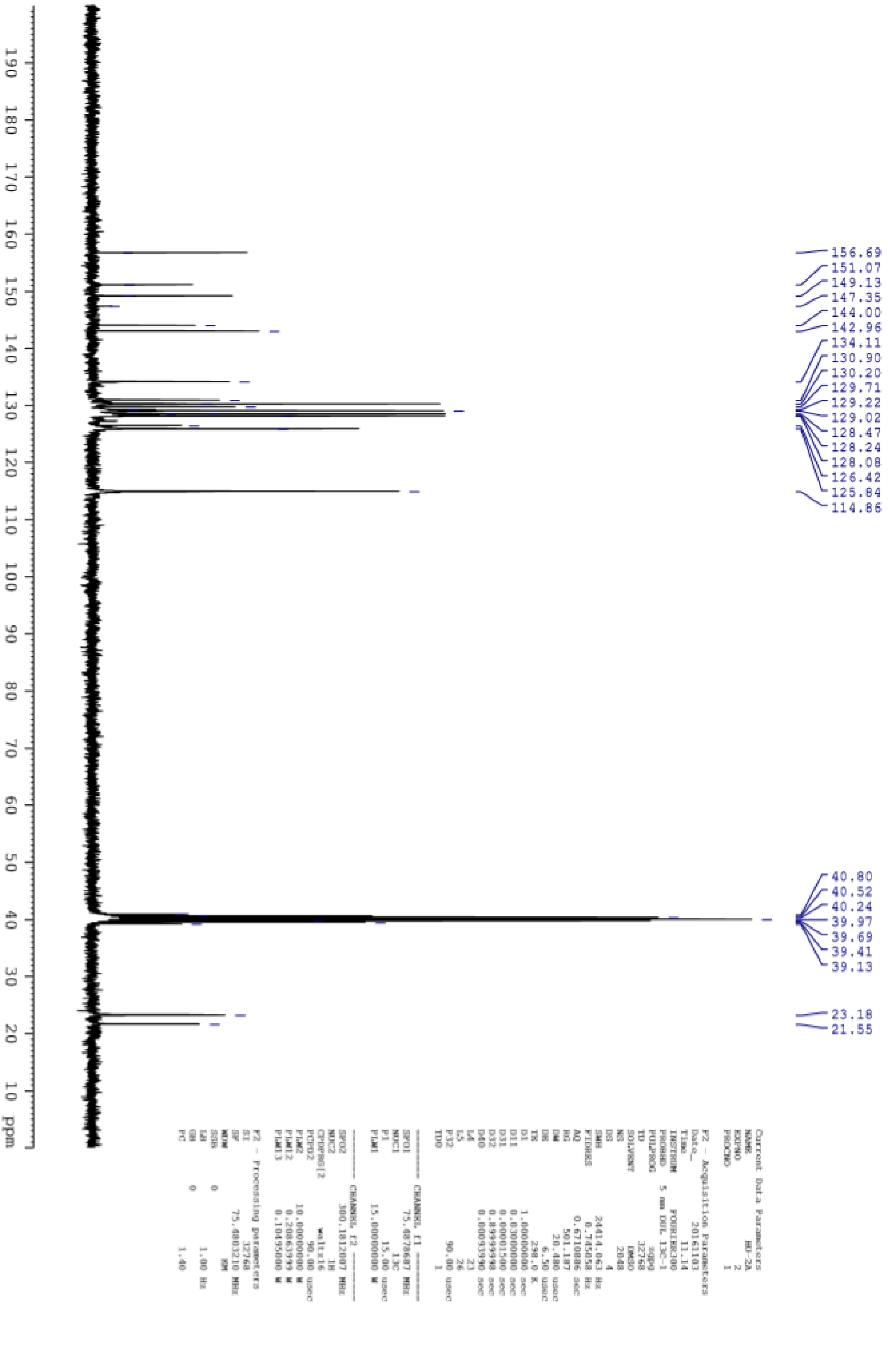

Figure S11. ^{13}C NMR spectrum of compound **5d**

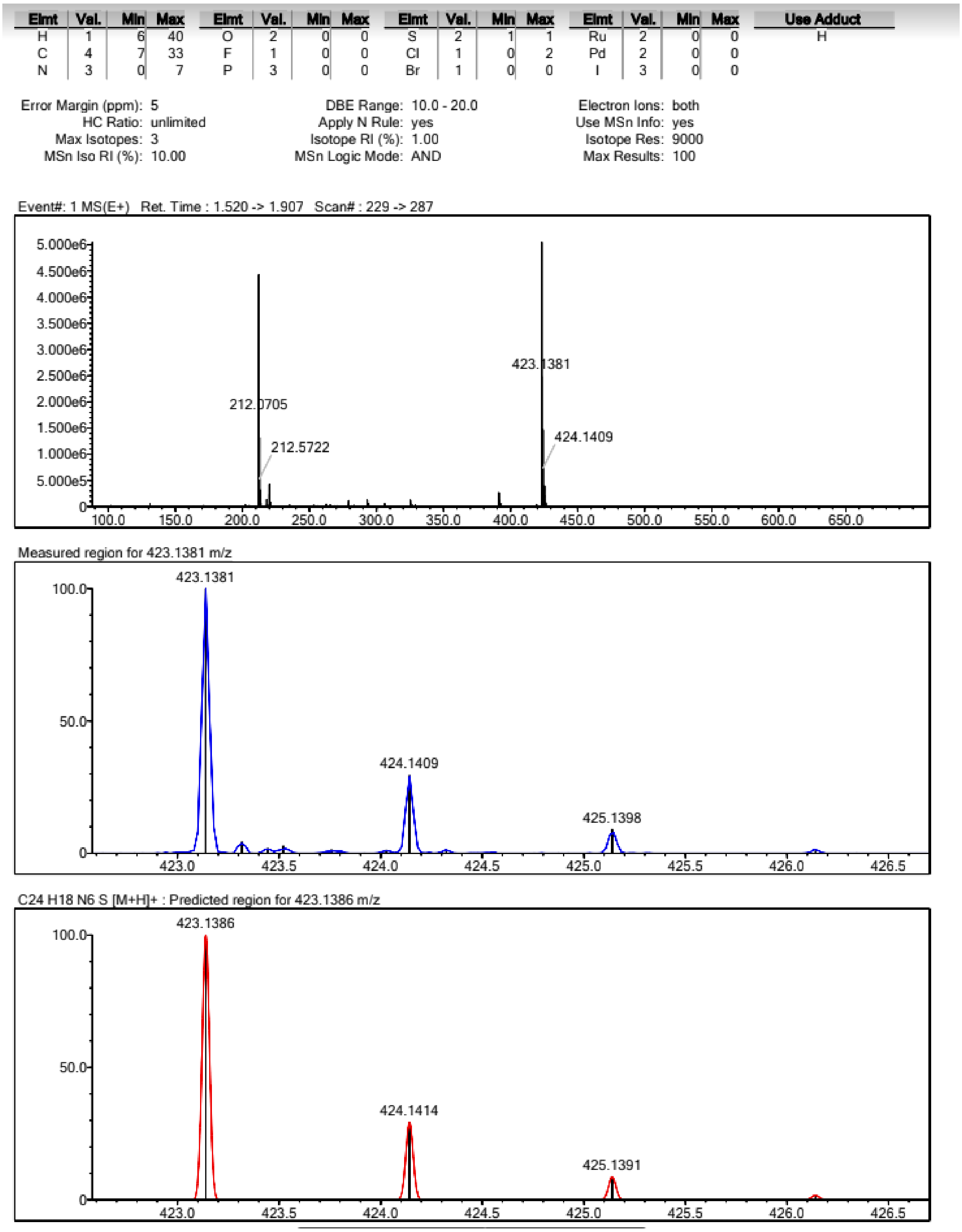

Figure S12. Mass spectrum of compound **5d**

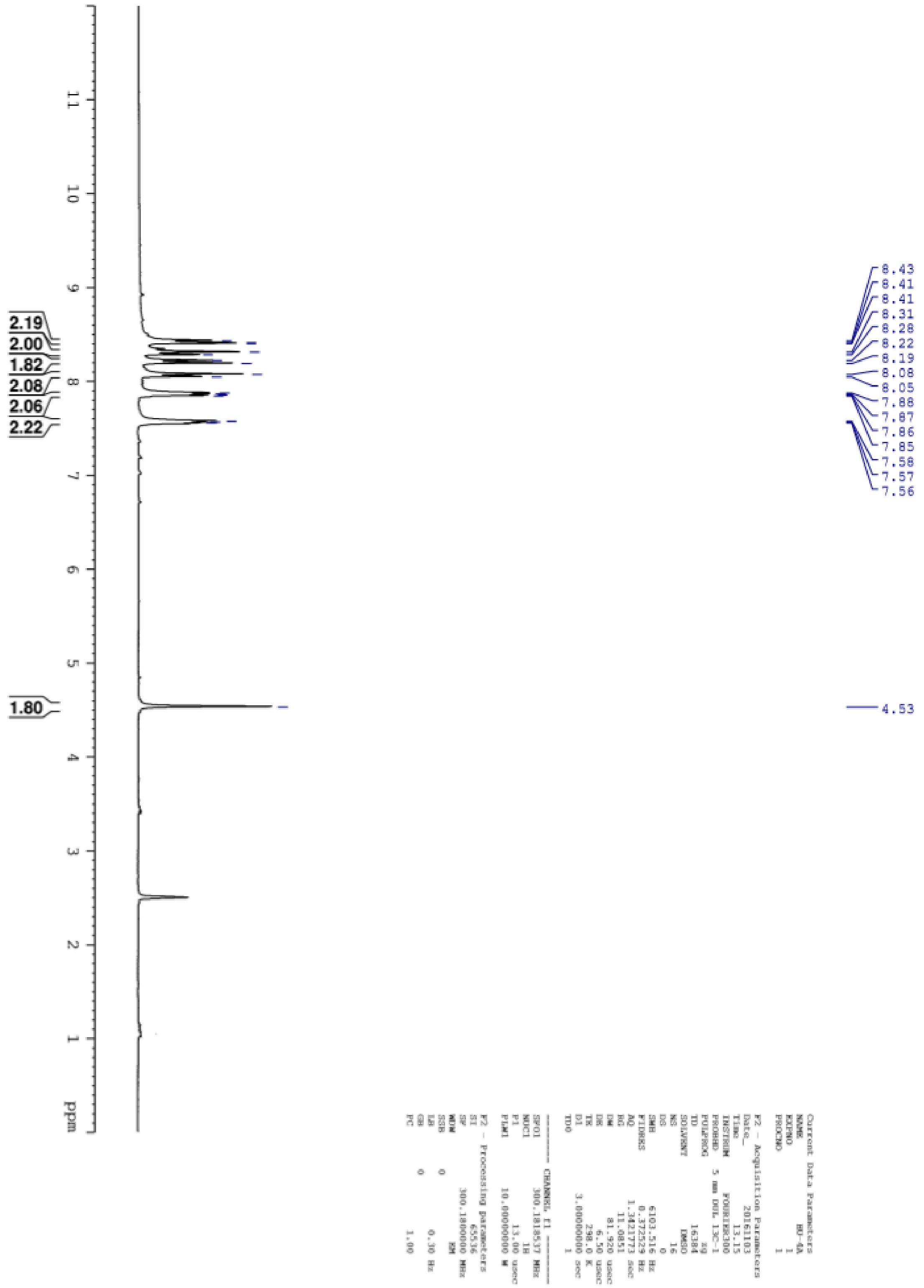

Figure S13. ^{1}H NMR spectrum of compound **5e**

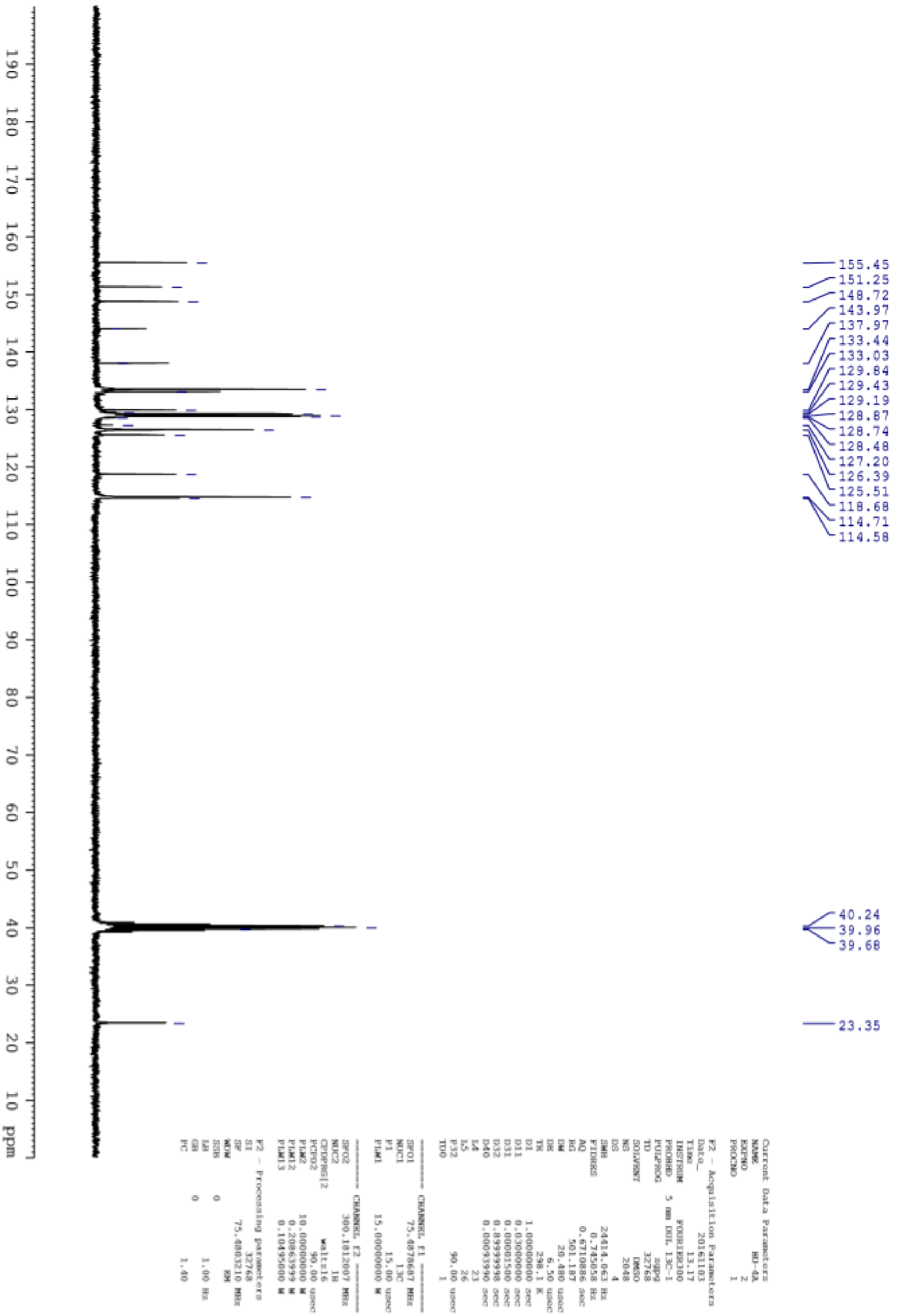

Figure S14. ^{13}C NMR spectrum of compound **5e**

Elmt	Val.	Min	Max	Elmt	Val.	Min	Max	Elmt	Val.	Min	Max	Elmt	Val.	Min	Max	Use Adduct
H	1	6	40	O	2	0	0	S	2	1	1	Ru	2	0	0	H
C	4	7	33	F	1	0	0	Cl	1	0	2	Pd	2	0	0	
N	3	0	7	P	3	0	0	Br	1	0	0	I	3	0	0	

Error Margin (ppm): 5
HC Ratio: unlimited
Max Isotopes: 3
MSn Iso RI (%): 10.00

DBE Range: 17.0 - 23.0
Apply N Rule: yes
Isotope RI (%): 1.00
MSn Logic Mode: AND

Electron Ions: both
Use MSn Info: yes
Isotope Res: 9000
Max Results: 100

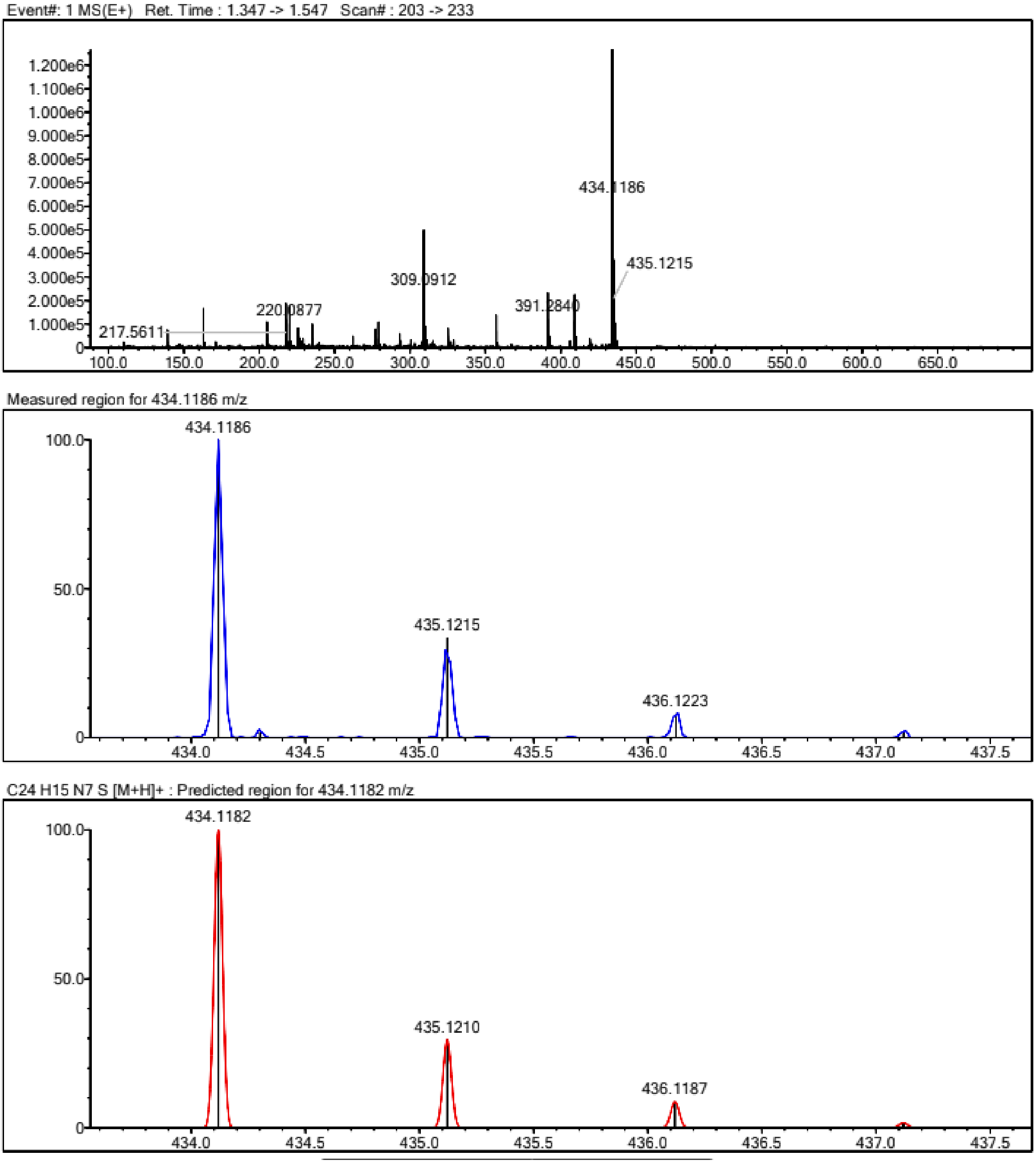

Figure S15. Mass spectrum of compound **5e**

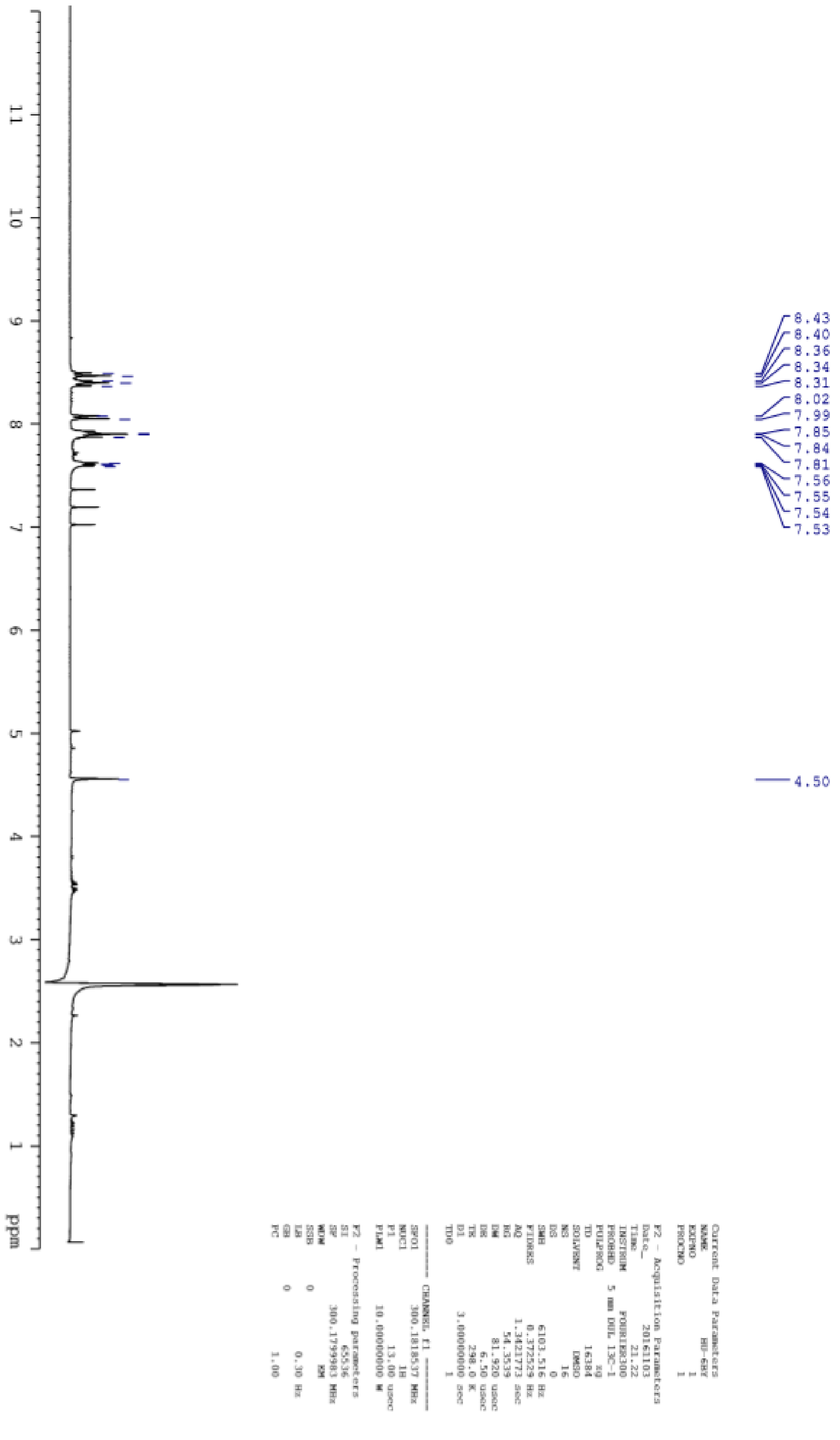

Figure S16. ^{1}H NMR spectrum of compound **5f**

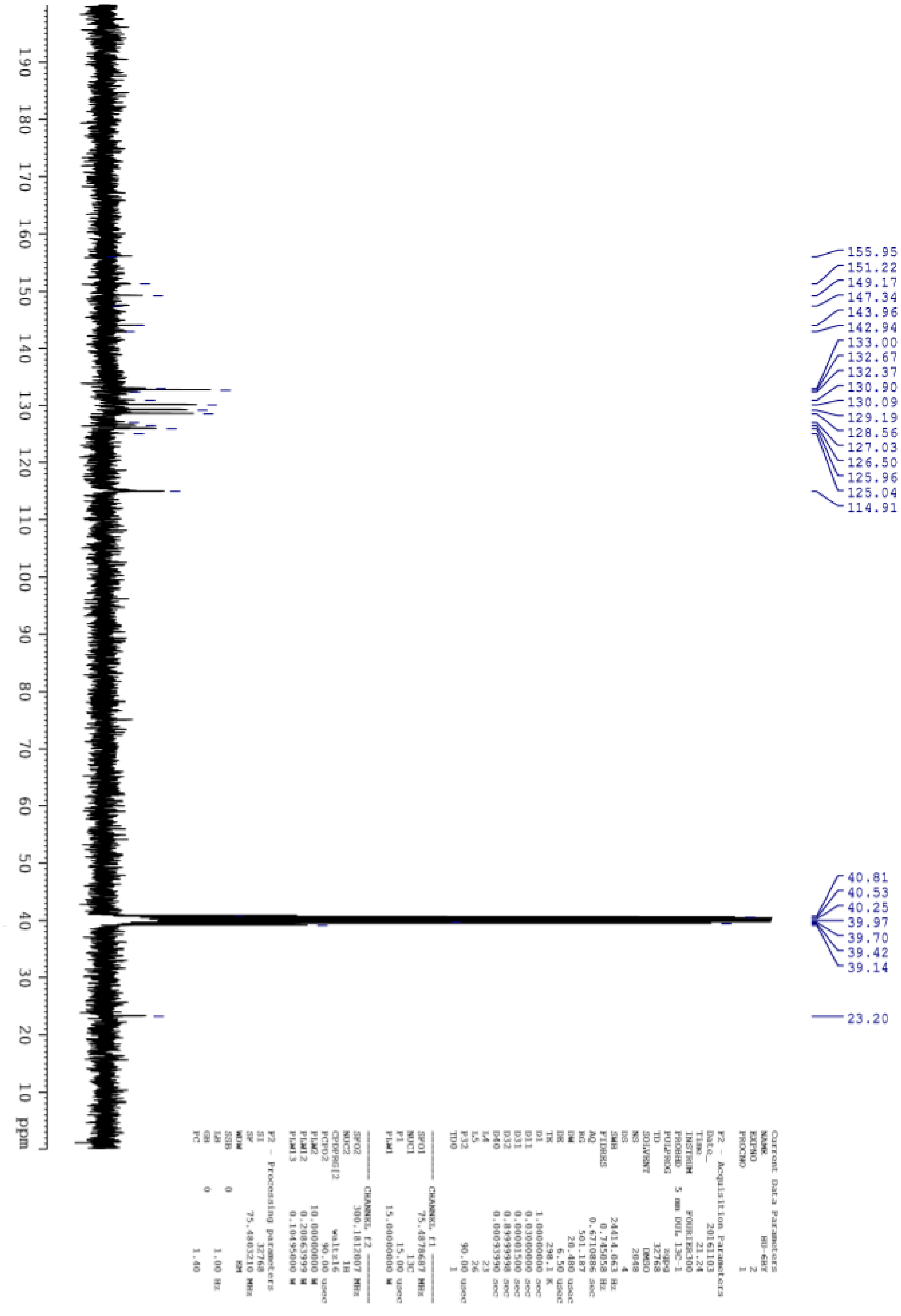

Figure S17. ^{13}C NMR spectrum of compound **5f**

Elmt	Val.	Min	Max	Elmt	Val.	Min	Max	Elmt	Val.	Min	Max	Elmt	Val.	Min	Max	Use Adduct
H	1	15	15	O	2	0	3	Cl	1	0	2	I	3	0	0	H
C	4	23	23	F	1	0	3	Br	1	0	1					
N	3	0	6	S	2	0	2	Ru	2	0	0					

Error Margin (ppm): 5
HC Ratio: unlimited
Max Isotopes: 3
MSn Iso RI (%): 10.00

DBE Range: 10.0 - 30.0
Apply N Rule: yes
Isotope RI (%): 1.00
MSn Logic Mode: AND

Electron Ions: both
Use MSn Info: no
Isotope Res: 10000
Max Results: 500

Event#: 1 MS(E+) Ret. Time : 5.187 -> 5.413 Scan# : 779 -> 813

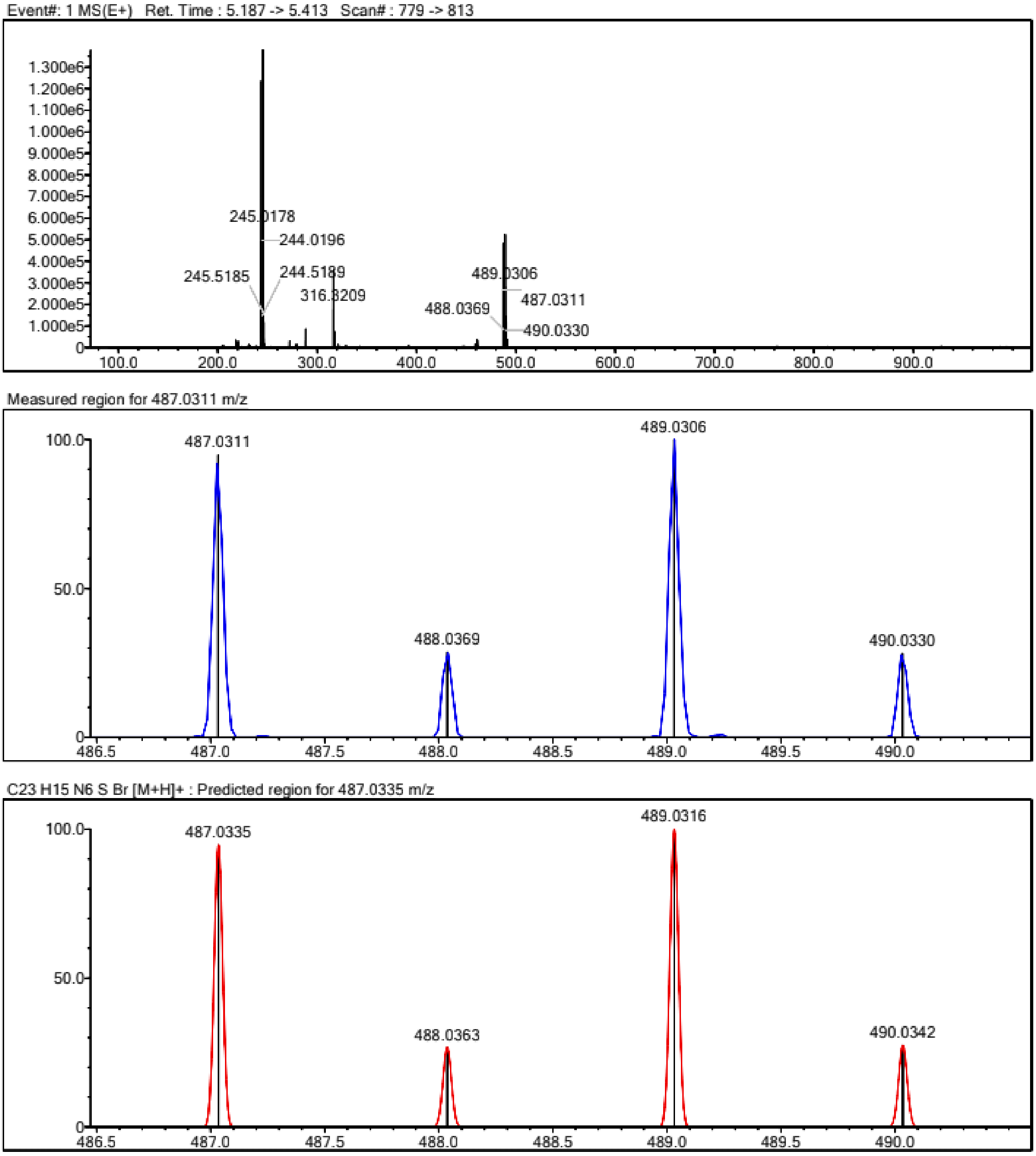

Figure S18. Mass spectrum of compound **5f**

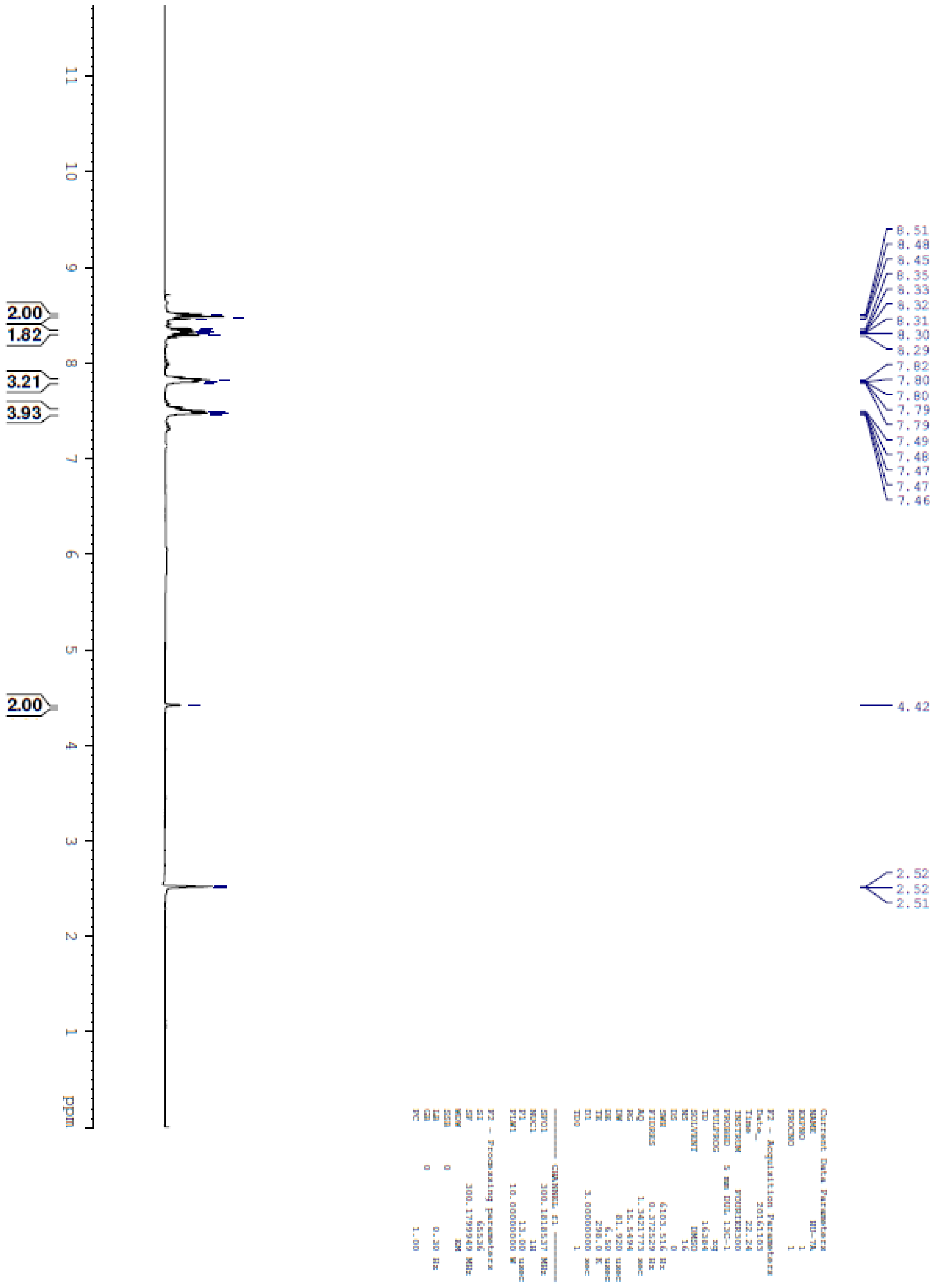

Figure S19. ^{1}H NMR spectrum of compound **5g**

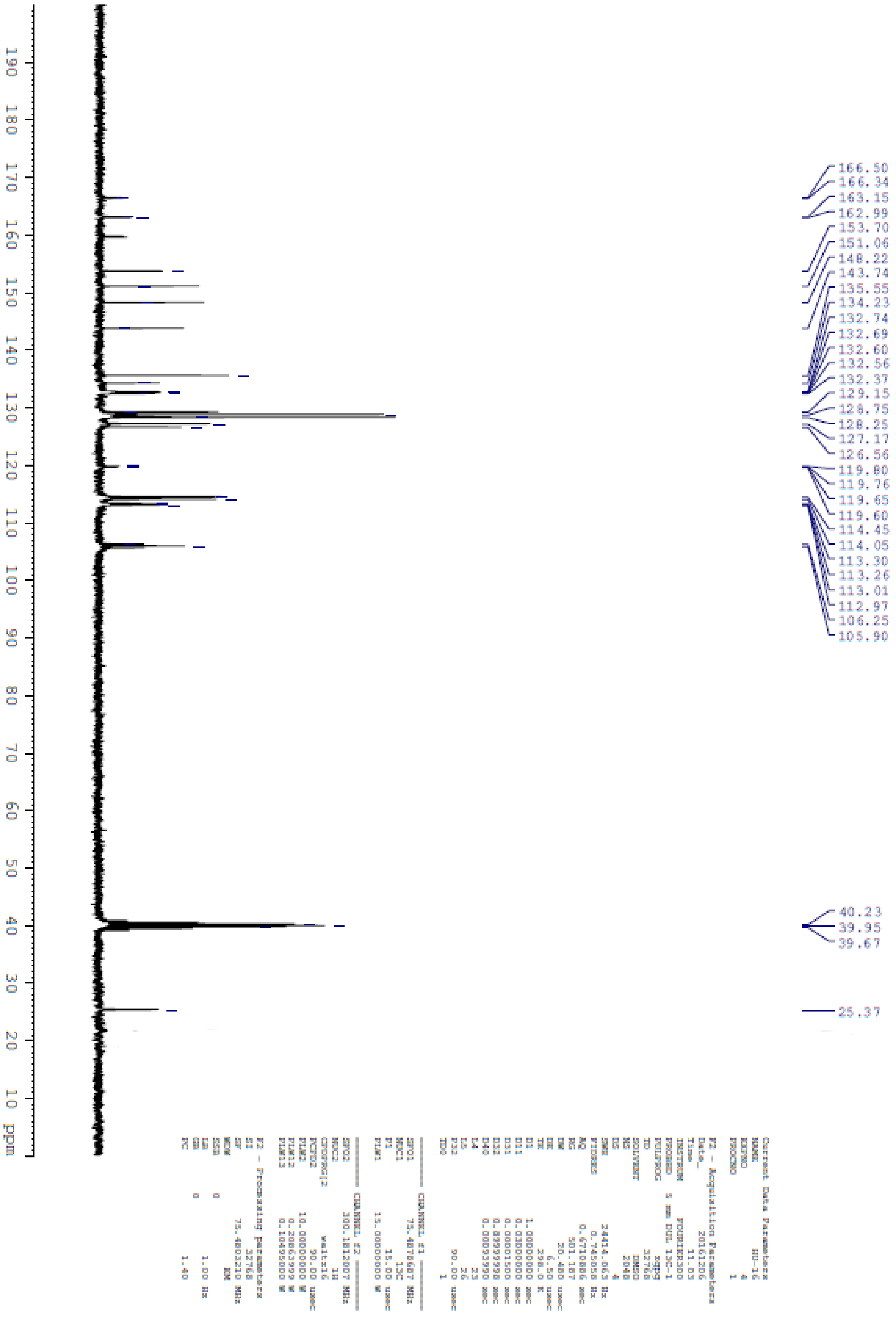

Figure S20. ^{13}C NMR spectrum of compound **5g**

Elmt	Val.	Min	Max	Elmt	Val.	Min	Max	Elmt	Val.	Min	Max	Elmt	Val.	Min	Max	Use Adduct
H	1	6	40	O	2	0	0	S	2	1	1	Ru	2	0	0	H
C	4	7	33	F	1	0	2	Cl	1	0	2	Pd	2	0	0	
N	3	0	7	P	3	0	0	Br	1	0	0	I	3	0	0	

Error Margin (ppm):	5	DBE Range:	17.0 - 23.0	Electron Ions:	both
HC Ratio:	unlimited	Apply N Rule:	yes	Use MSn Info:	yes
Max Isotopes:	3	Isotope RI (%):	1.00	Isotope Res:	9000
MSn Iso RI (%):	10.00	MSn Logic Mode:	AND	Max Results:	100

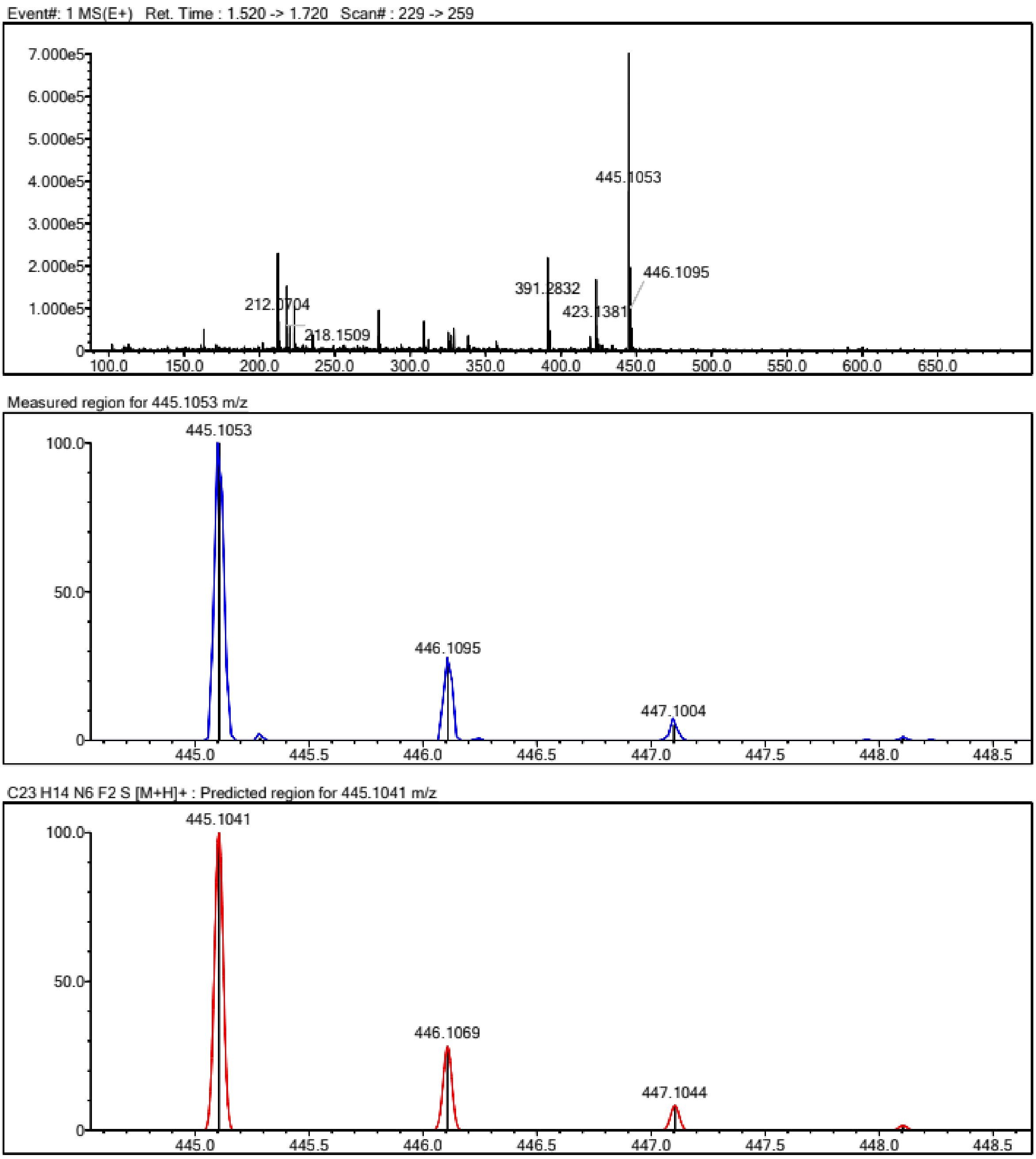

Figure S21. Mass spectrum of compound **5g**

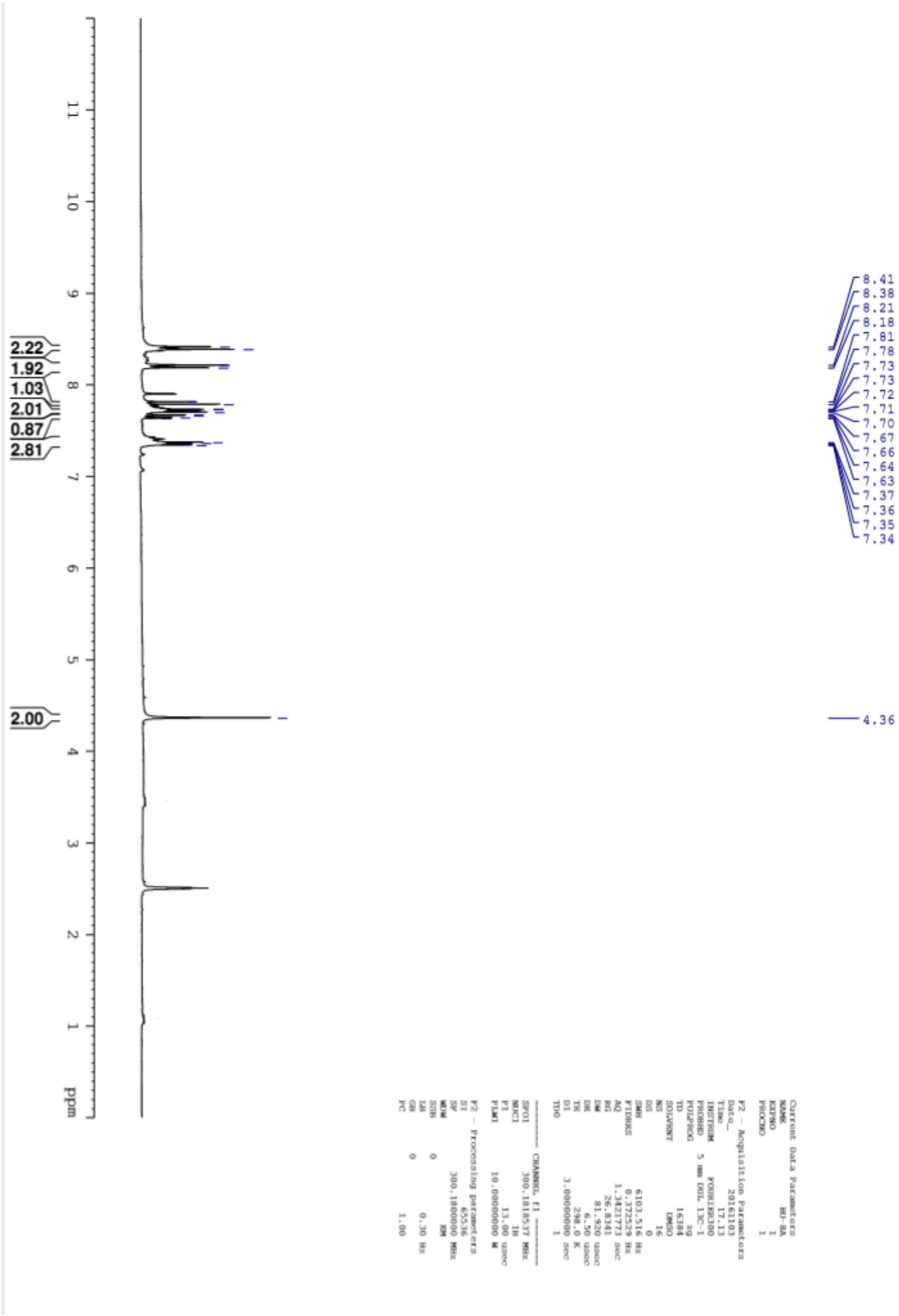

Figure S22. ^{1}H NMR spectrum of compound **5h**

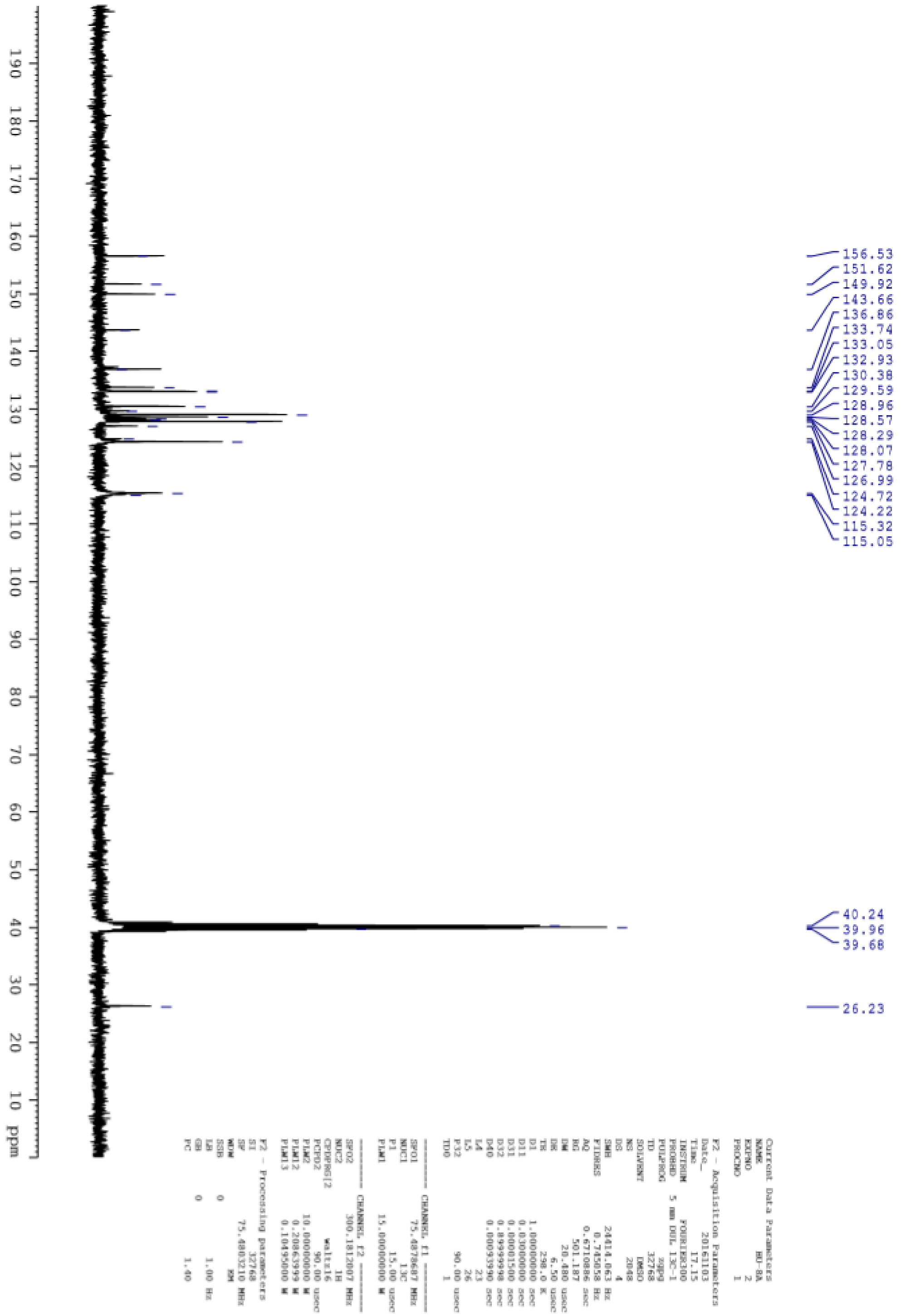

Figure S23. ^{13}C NMR spectrum of compound **5h**

Elmt	Val.	Min	Max	Elmt	Val.	Min	Max	Elmt	Val.	Min	Max	Elmt	Val.	Min	Max	Use Adduct
H	1	6	40	O	2	0	0	S	2	1	1	Ru	2	0	0	H
C	4	7	33	F	1	0	0	Cl	1	0	2	Pd	2	0	0	
N	3	0	7	P	3	0	0	Br	1	0	0	I	3	0	0	

Error Margin (ppm): 15
HC Ratio: unlimited
Max Isotopes: 3
MSn Iso RI (%): 10.00

DBE Range: 17.0 - 23.0
Apply N Rule: yes
Isotope RI (%): 1.00
MSn Logic Mode: AND

Electron Ions: both
Use MSn Info: yes
Isotope Res: 9000
Max Results: 100

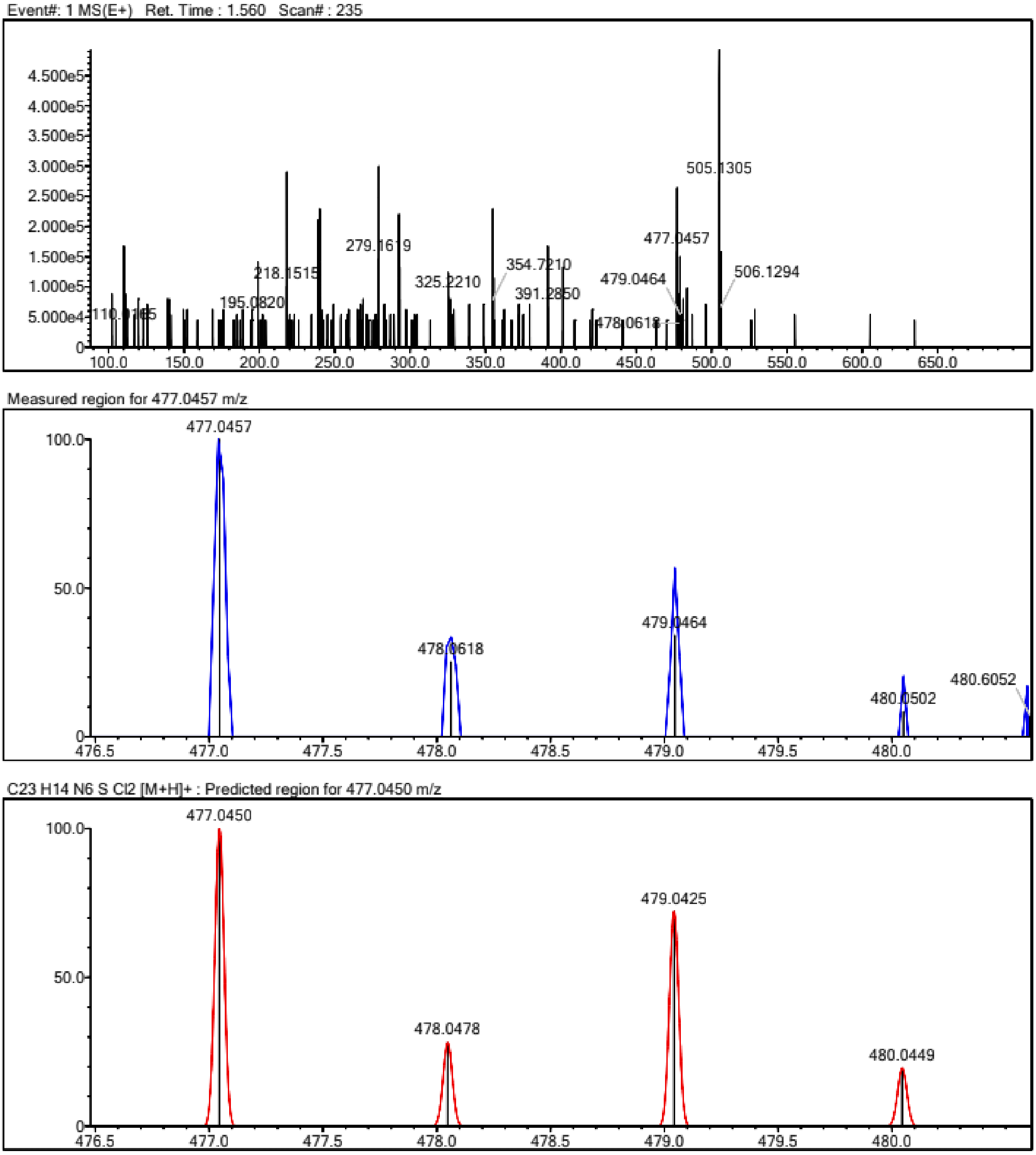

Figure S24. Mass spectrum of compound **5h**

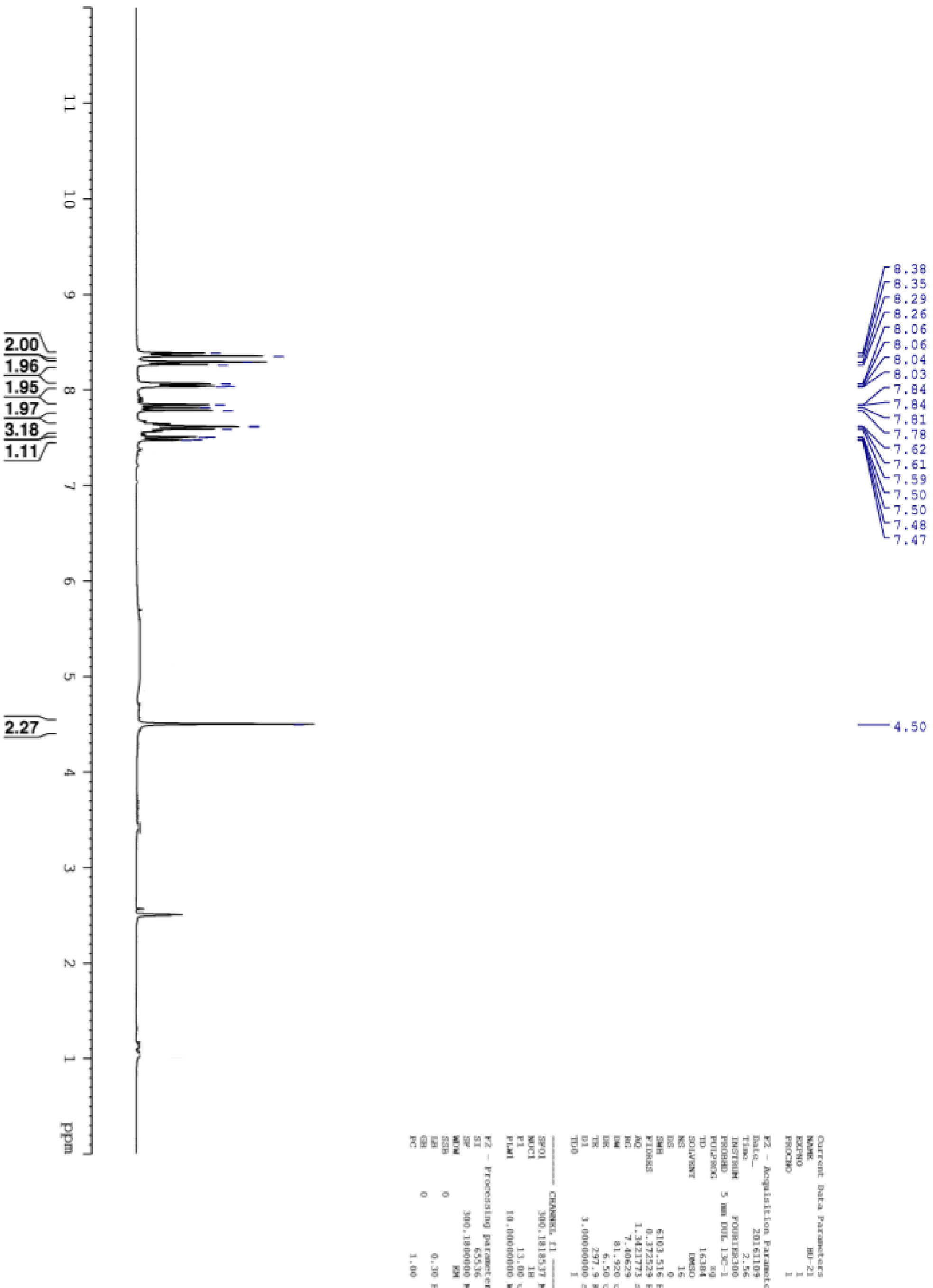

Figure S25. ^{1}H NMR spectrum of compound **51**

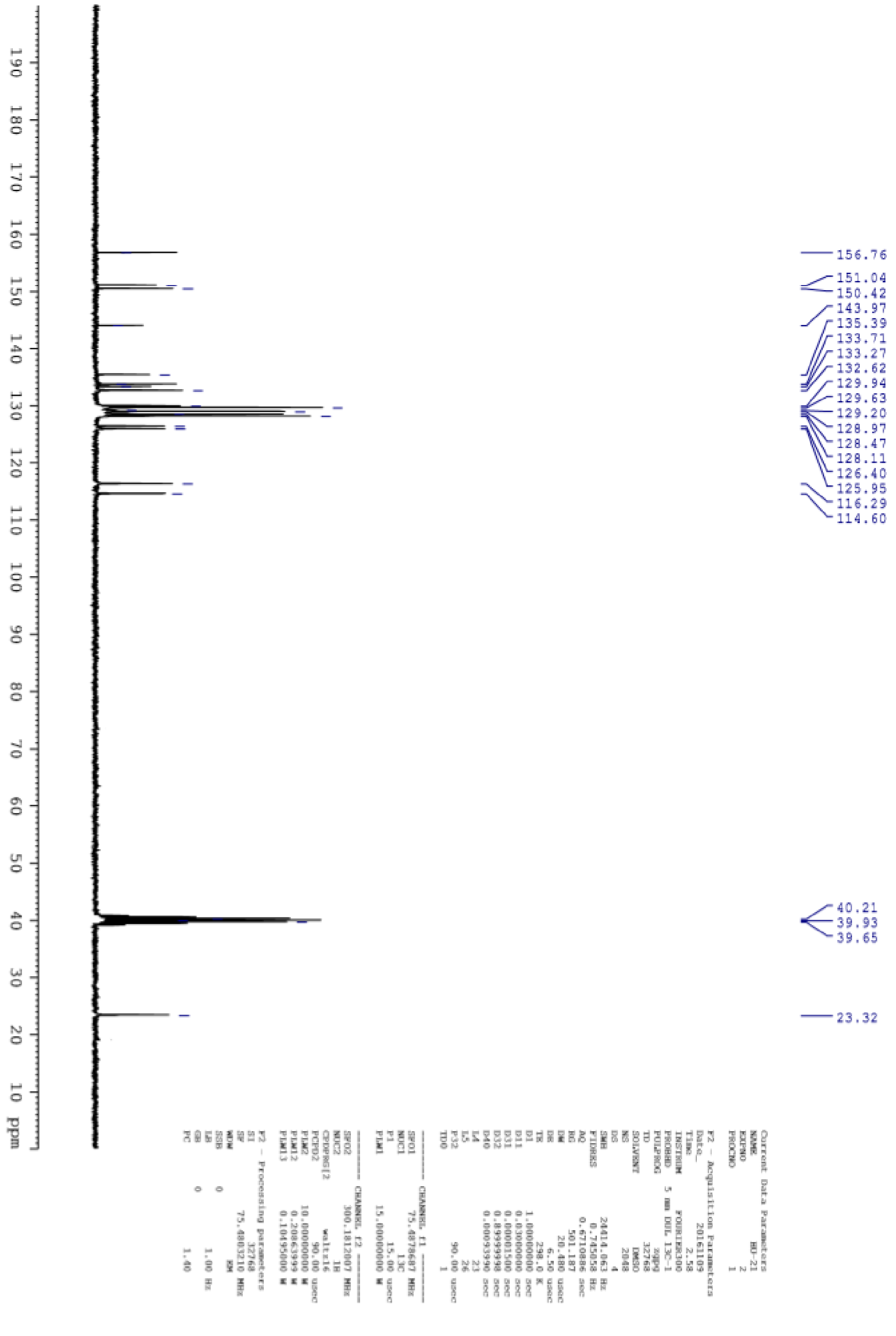

Figure S26. ^{13}C NMR spectrum of compound **51**

Elmt	Val.	Min	Max	Elmt	Val.	Min	Max	Elmt	Val.	Min	Max	Elmt	Val.	Min	Max	Use Adduct
H	1	15	15	O	2	0	3	Cl	1	0	2	I	3	0	0	H
C	4	23	24	F	1	0	2	Br	1	0	1					
N	3	0	7	S	2	0	2	Ru	2	0	0					

Error Margin (ppm):	5	DBE Range:	10.0 - 30.0	Electron Ions:	both
HC Ratio:	unlimited	Apply N Rule:	yes	Use MSn Info:	no
Max Isotopes:	3	Isotope RI (%):	1.00	Isotope Res:	10000
MSn Iso RI (%):	10.00	MSn Logic Mode:	AND	Max Results:	500

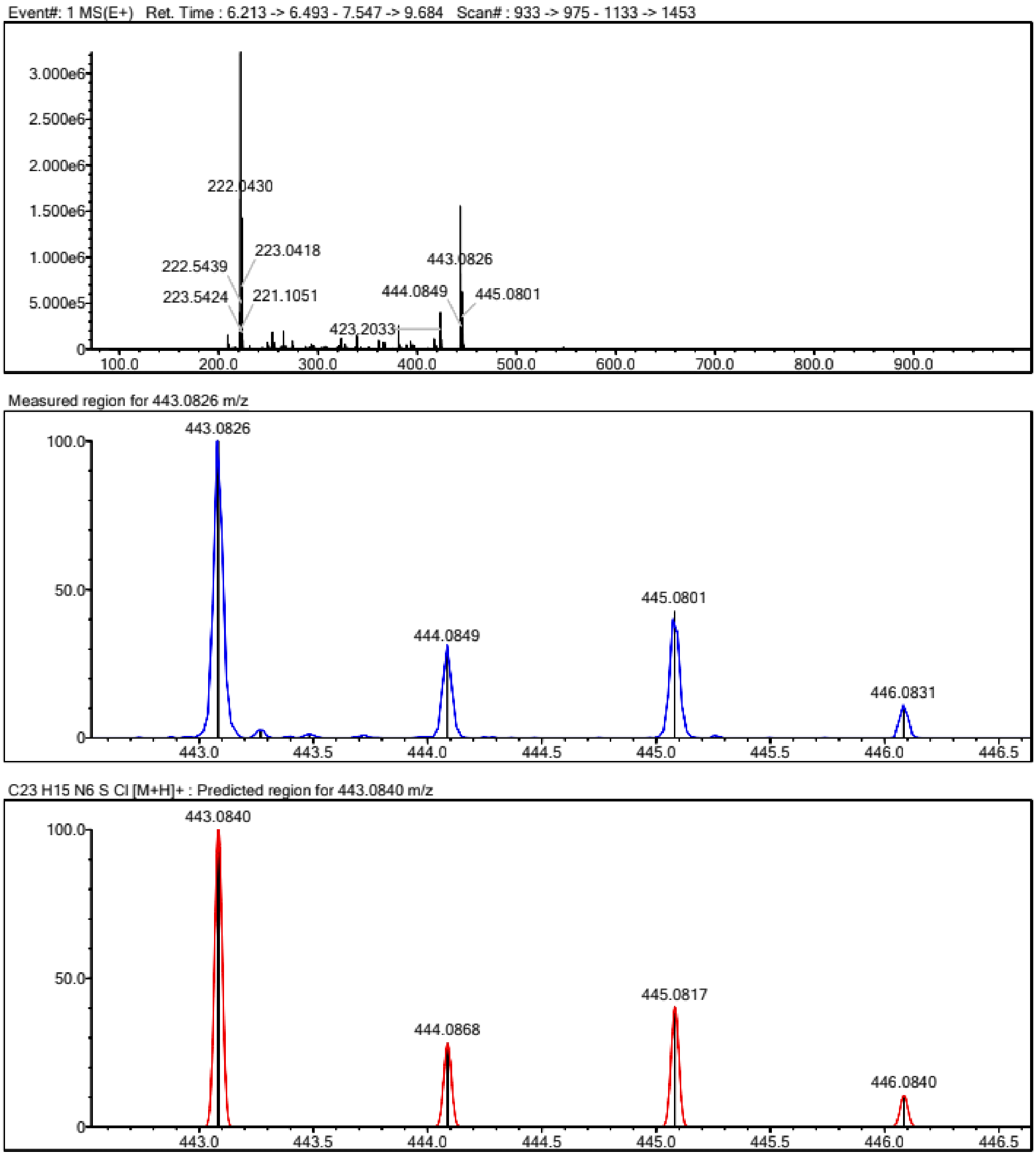

Figure S27. Mass spectrum of compound **51**

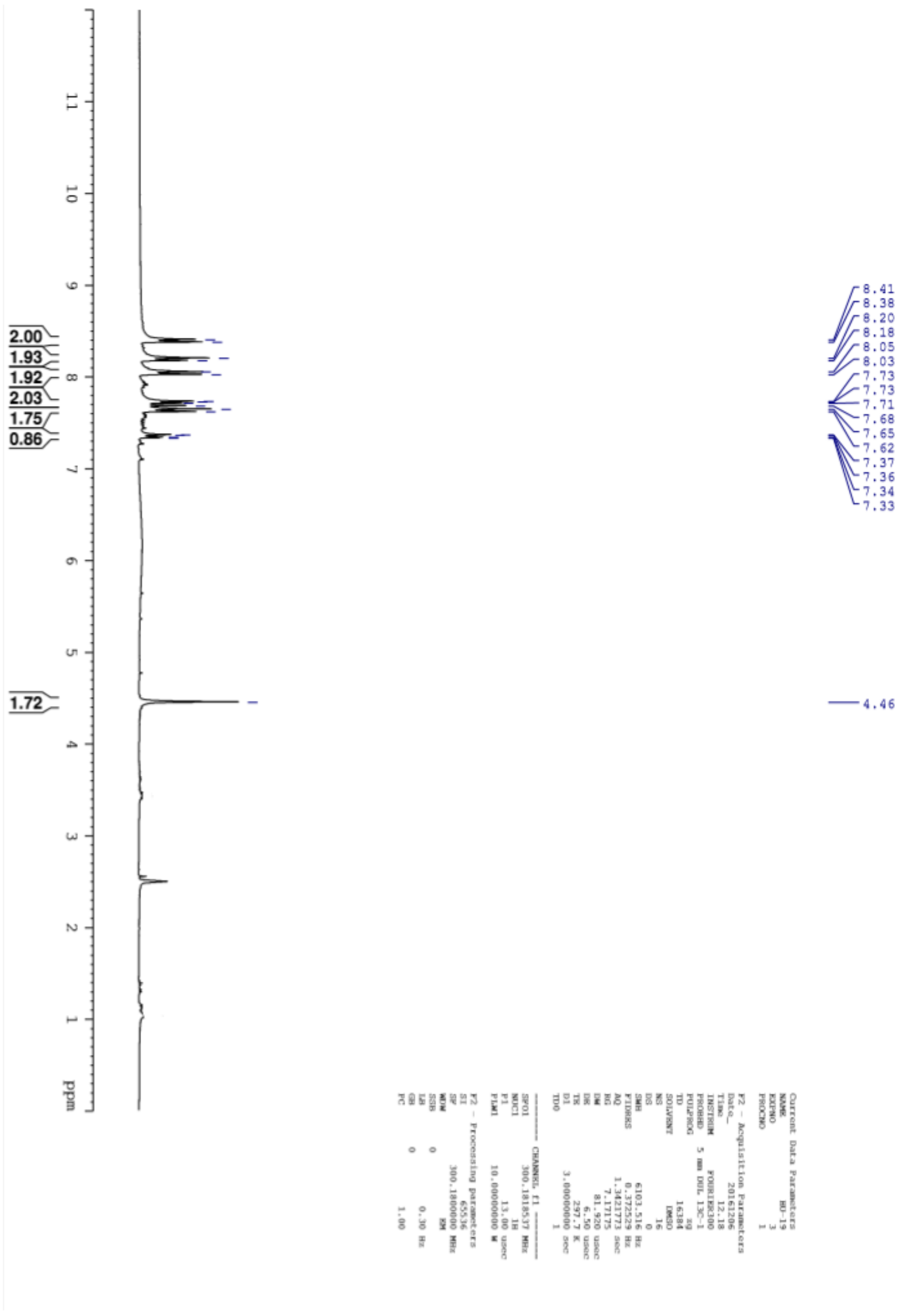

Figure S28. ^{1}H NMR spectrum of compound **5j**

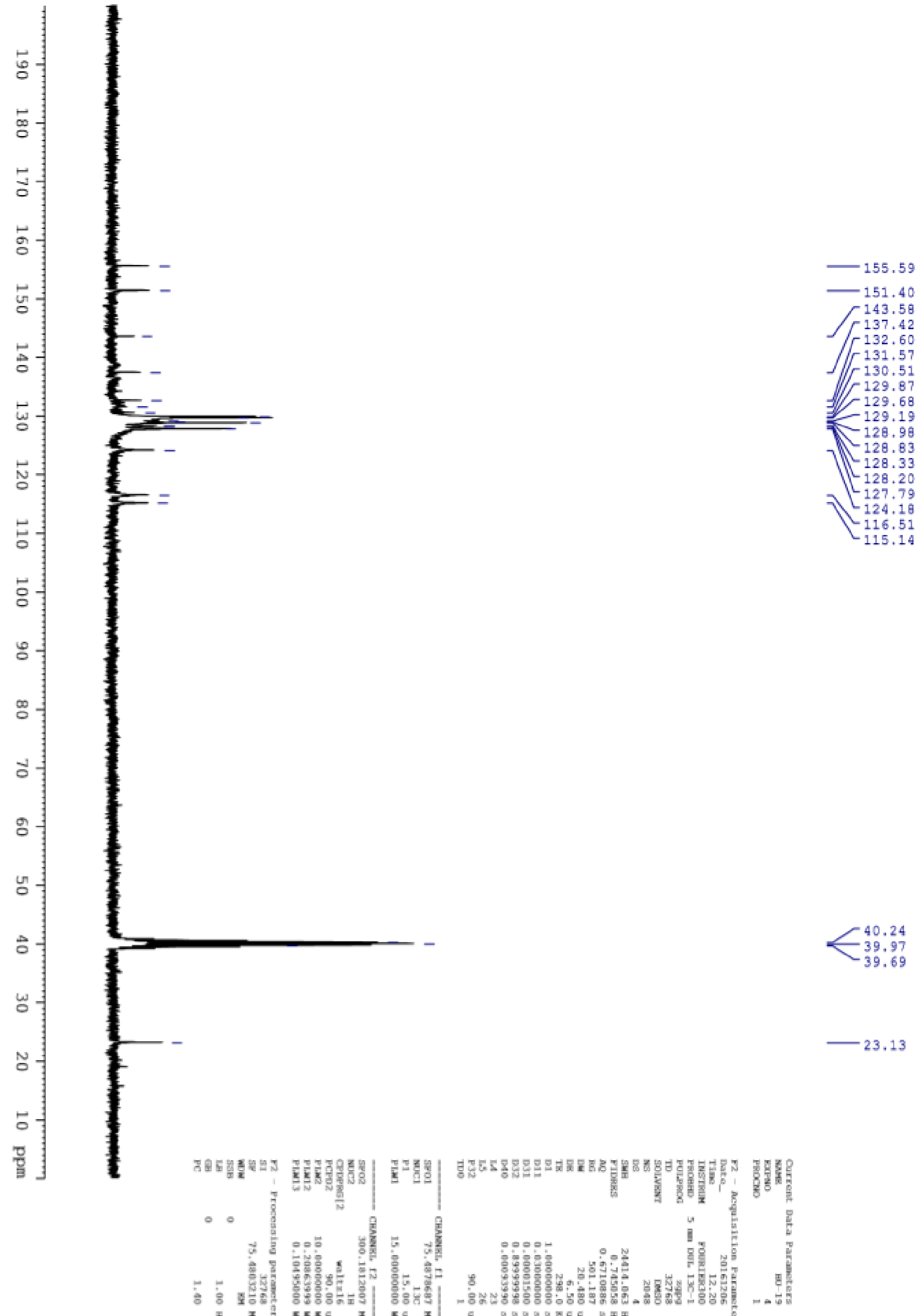

Figure S29. ^{13}C NMR spectrum of compound **5j**

Elmt	Val.	Min	Max	Elmt	Val.	Min	Max	Elmt	Val.	Min	Max	Elmt	Val.	Min	Max	Use Adduct
H	1	13	16	O	2	0	0	Cl	1	0	3	I	3	0	0	H
C	4	23	24	F	1	0	2	Br	1	0	1					
N	3	6	7	S	2	0	1	Ru	2	0	0					

Error Margin (ppm): 5
HC Ratio: unlimited
Max Isotopes: 3
MSn Iso RI (%): 10.00

DBE Range: 10.0 - 30.0
Apply N Rule: yes
Isotope RI (%): 1.00
MSn Logic Mode: AND

Electron Ions: both
Use MSn Info: no
Isotope Res: 10000
Max Results: 500

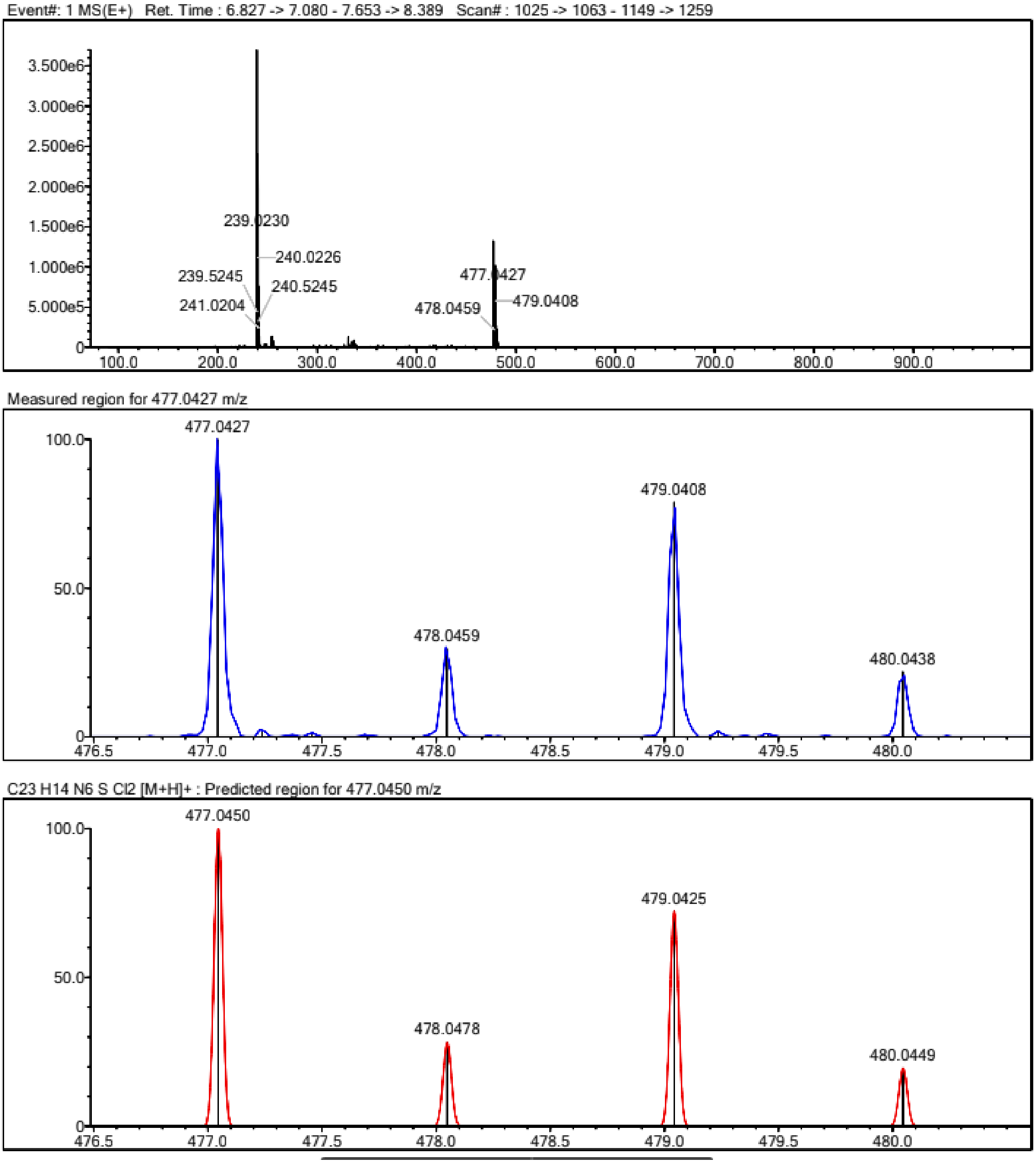

Figure S30. Mass spectrum of compound **5j**

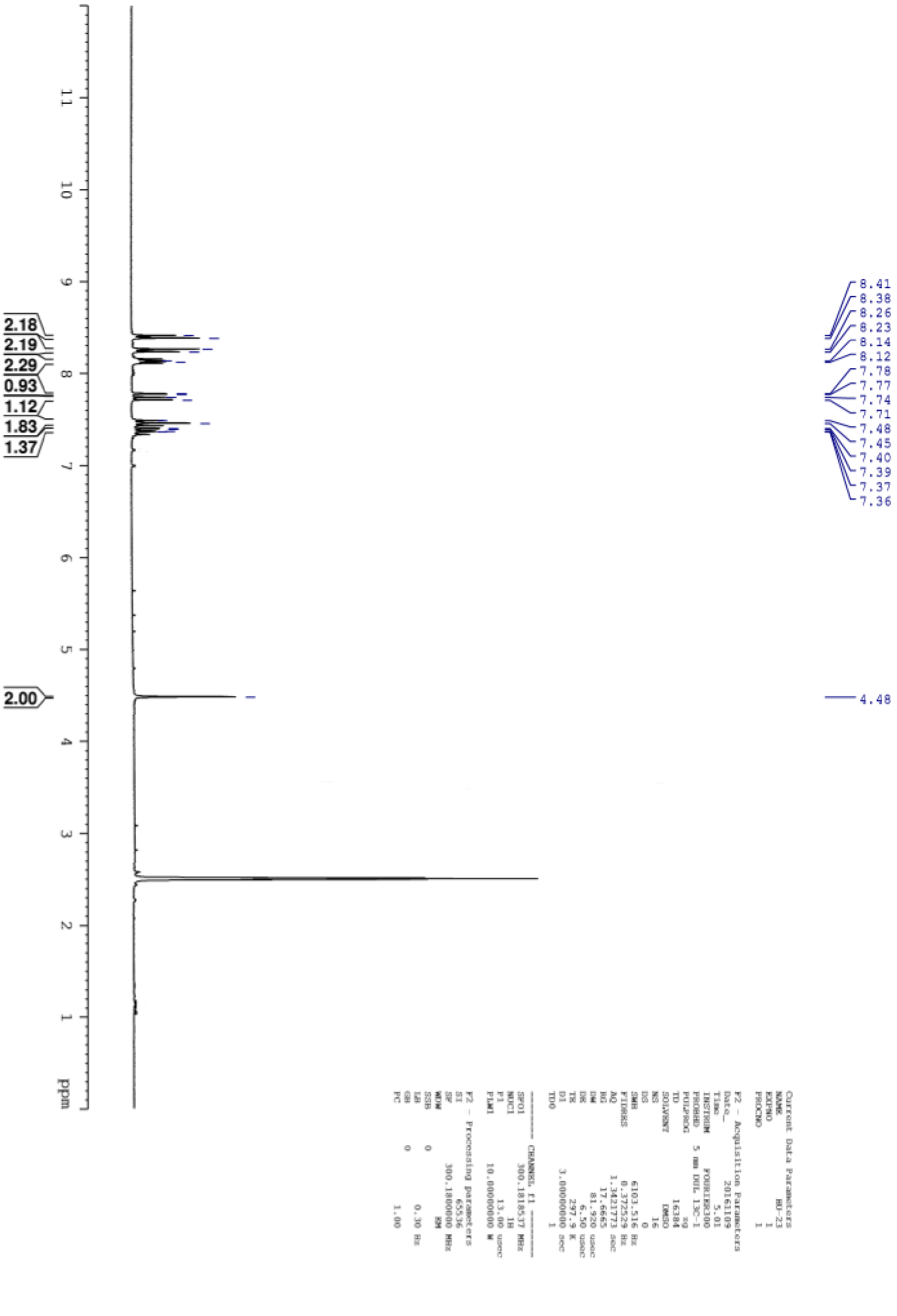

Figure S31. ^{1}H NMR spectrum of compound **5k**

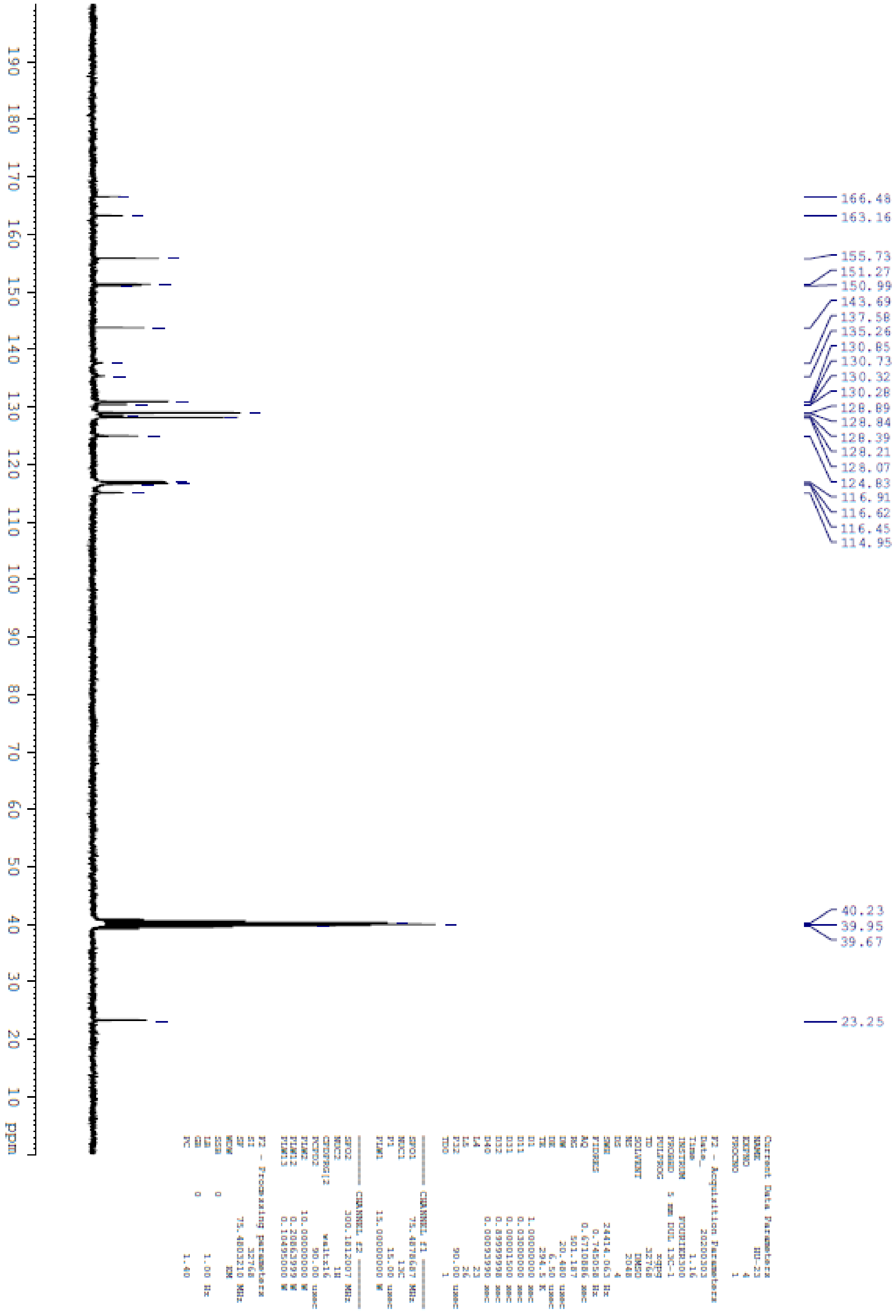

Figure S32. ^{13}C NMR spectrum of compound **5k**

Elmt	Val.	Min	Max	Elmt	Val.	Min	Max	Elmt	Val.	Min	Max	Elmt	Val.	Min	Max	Use Adduct
H	1	14	15	O	2	0	3	Cl	1	0	2	I	3	0	0	H
C	4	23	24	F	1	0	2	Br	1	0	1					
N	3	0	7	S	2	0	2	Ru	2	0	0					

Error Margin (ppm): 5
HC Ratio: unlimited
Max Isotopes: 3
MSn Iso RI (%): 10.00

DBE Range: 10.0 - 30.0
Apply N Rule: yes
Isotope RI (%): 1.00
MSn Logic Mode: AND

Electron Ions: both
Use MSn Info: no
Isotope Res: 10000
Max Results: 500

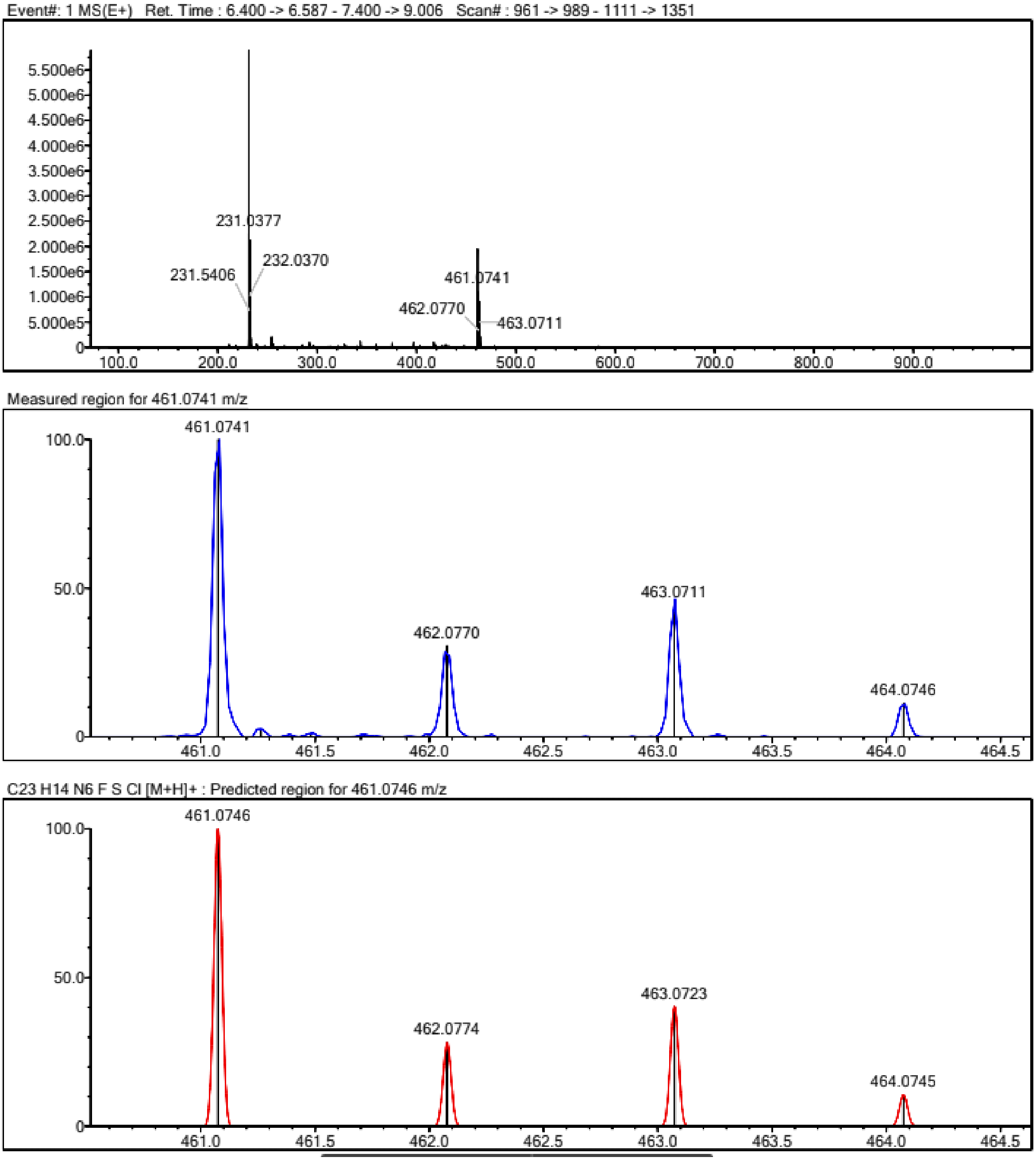

Figure S33. Mass spectrum of compound **5k**

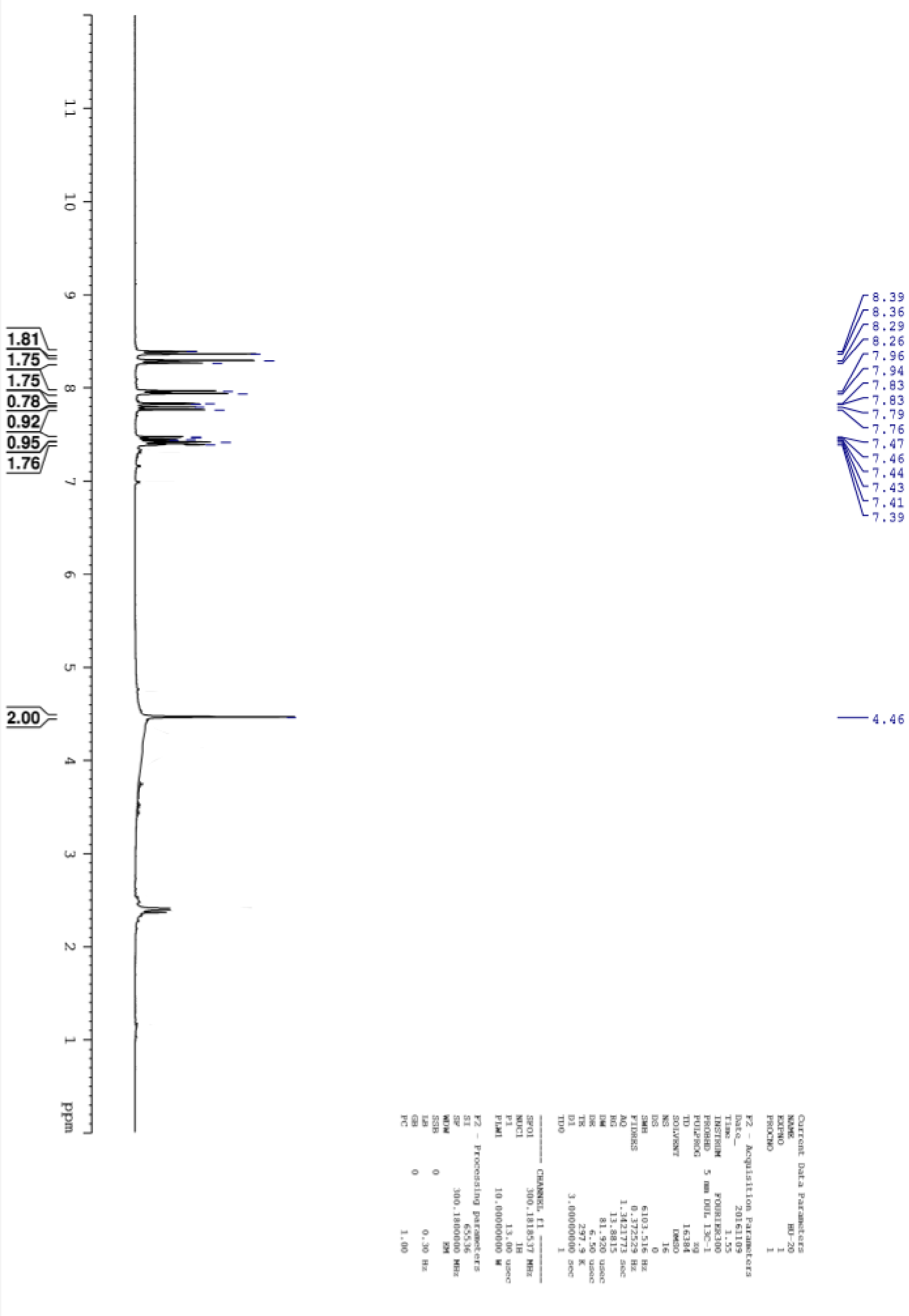

Figure S34. ^{1}H NMR spectrum of compound **5l**

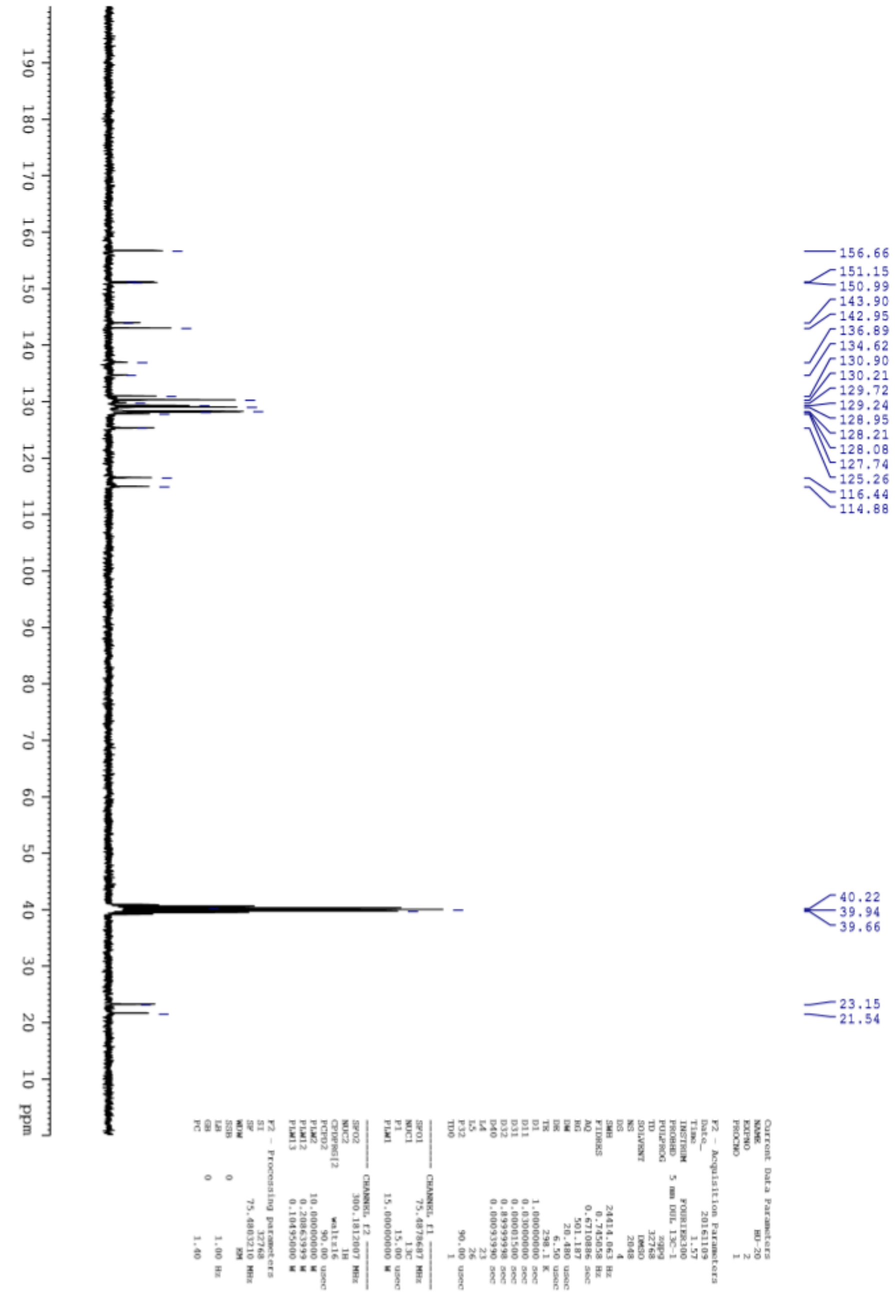

Figure S35. ^{13}C NMR spectrum of compound **5l**

Elmt	Val.	Min	Max	Elmt	Val.	Min	Max	Elmt	Val.	Min	Max	Elmt	Val.	Min	Max	Use Adduct
H	1	17	20	O	2	0	3	Cl	1	0	2	I	3	0	0	H
C	4	24	24	F	1	0	2	Br	1	0	1					
N	3	0	7	S	2	0	2	Ru	2	0	0					

Error Margin (ppm):	5	DBE Range:	10.0 - 30.0	Electron Ions:	both
HC Ratio:	unlimited	Apply N Rule:	yes	Use MSn Info:	no
Max Isotopes:	3	Isotope RI (%):	1.00	Isotope Res:	10000
MSn Iso RI (%):	10.00	MSn Logic Mode:	AND	Max Results:	500

Event#: 1 MS(E+) Ret. Time : 6.587 -> 6.693 - 8.253 -> 9.766 Scan# : 989 -> 1005 - 1239 -> 1465

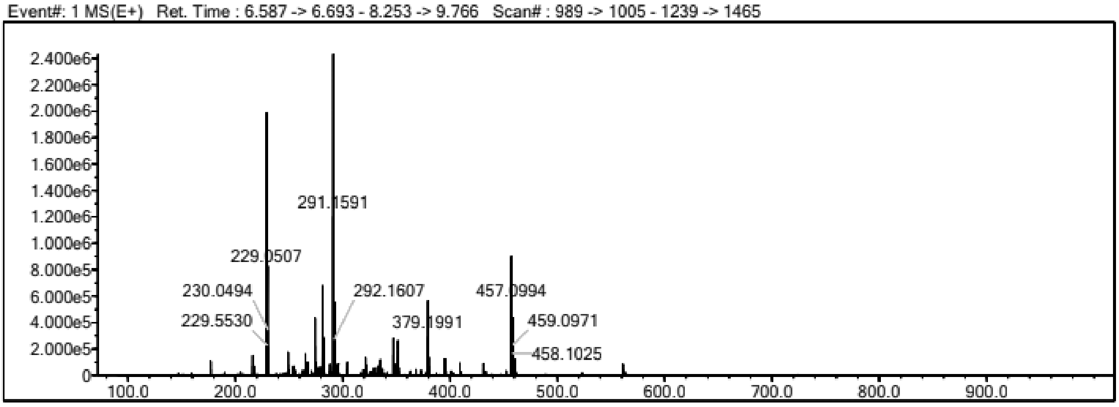

Measured region for 457.0994 m/z

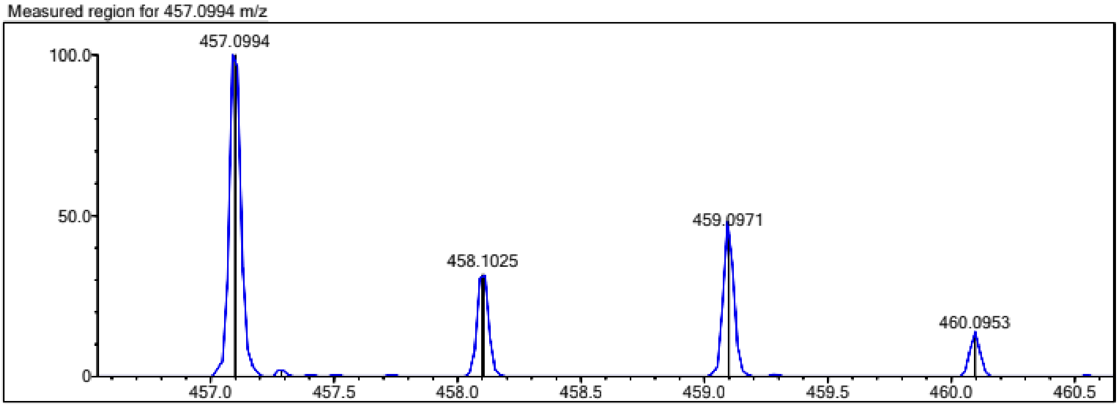

C24 H17 N6 S Cl [M+H]+ : Predicted region for 457.0997 m/z

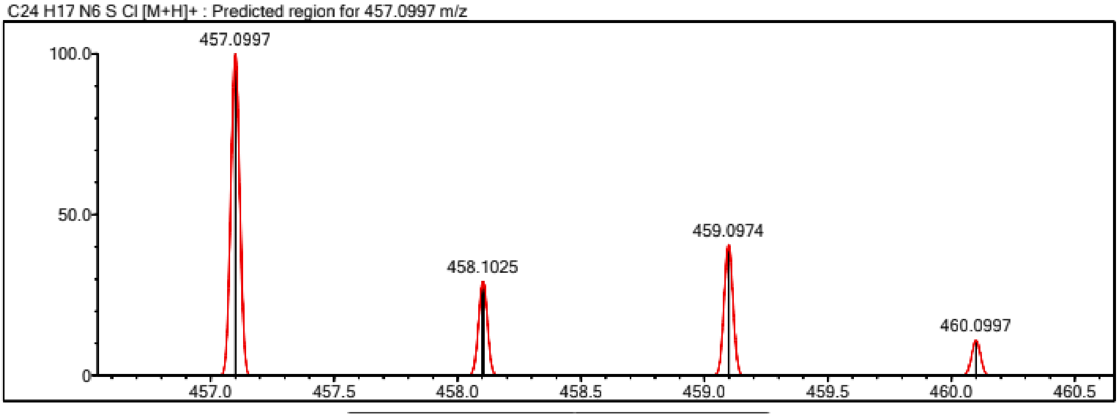

Figure S36. Mass spectrum of compound **5l**

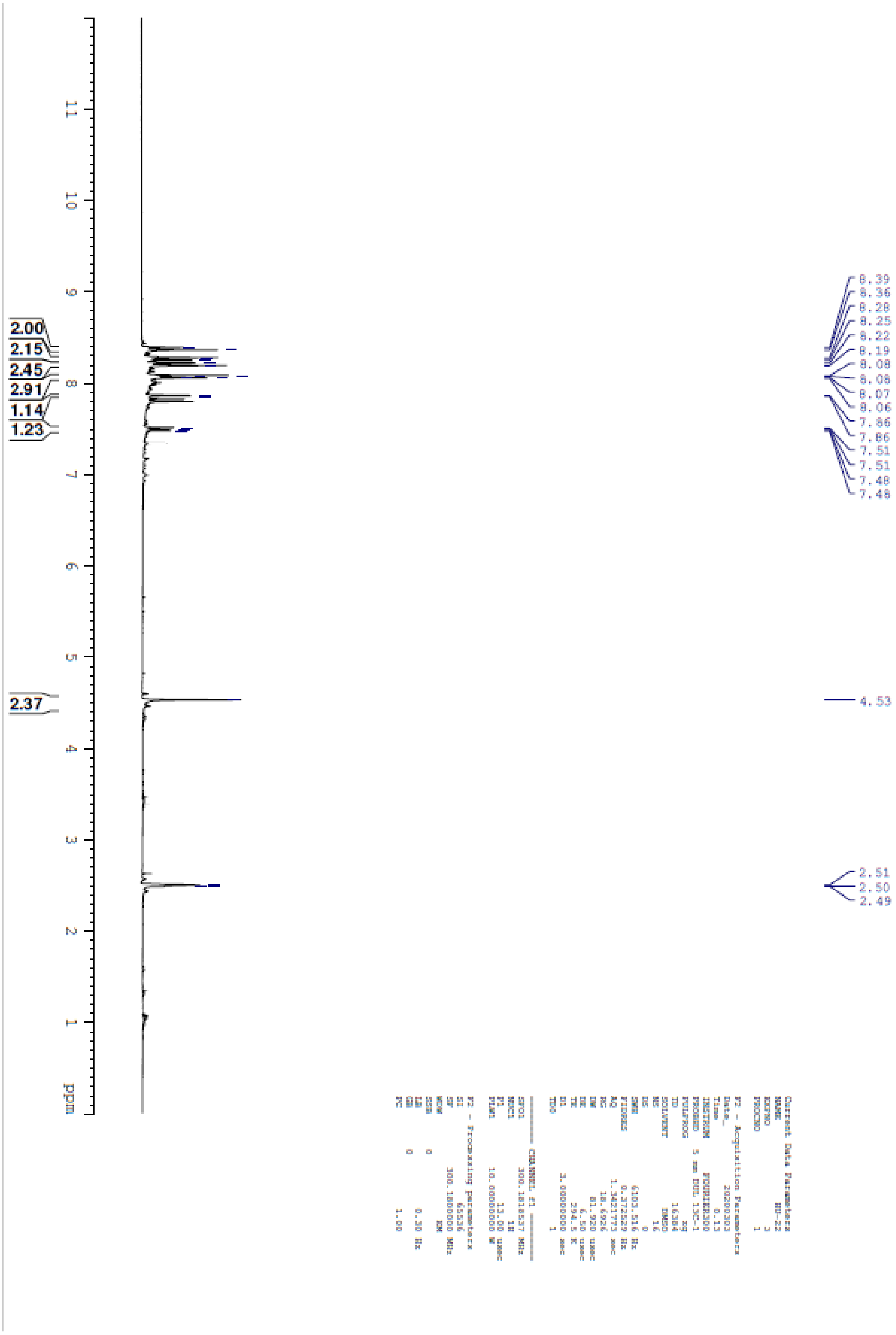

Figure S37. ^{1}H NMR spectrum of compound **5m**

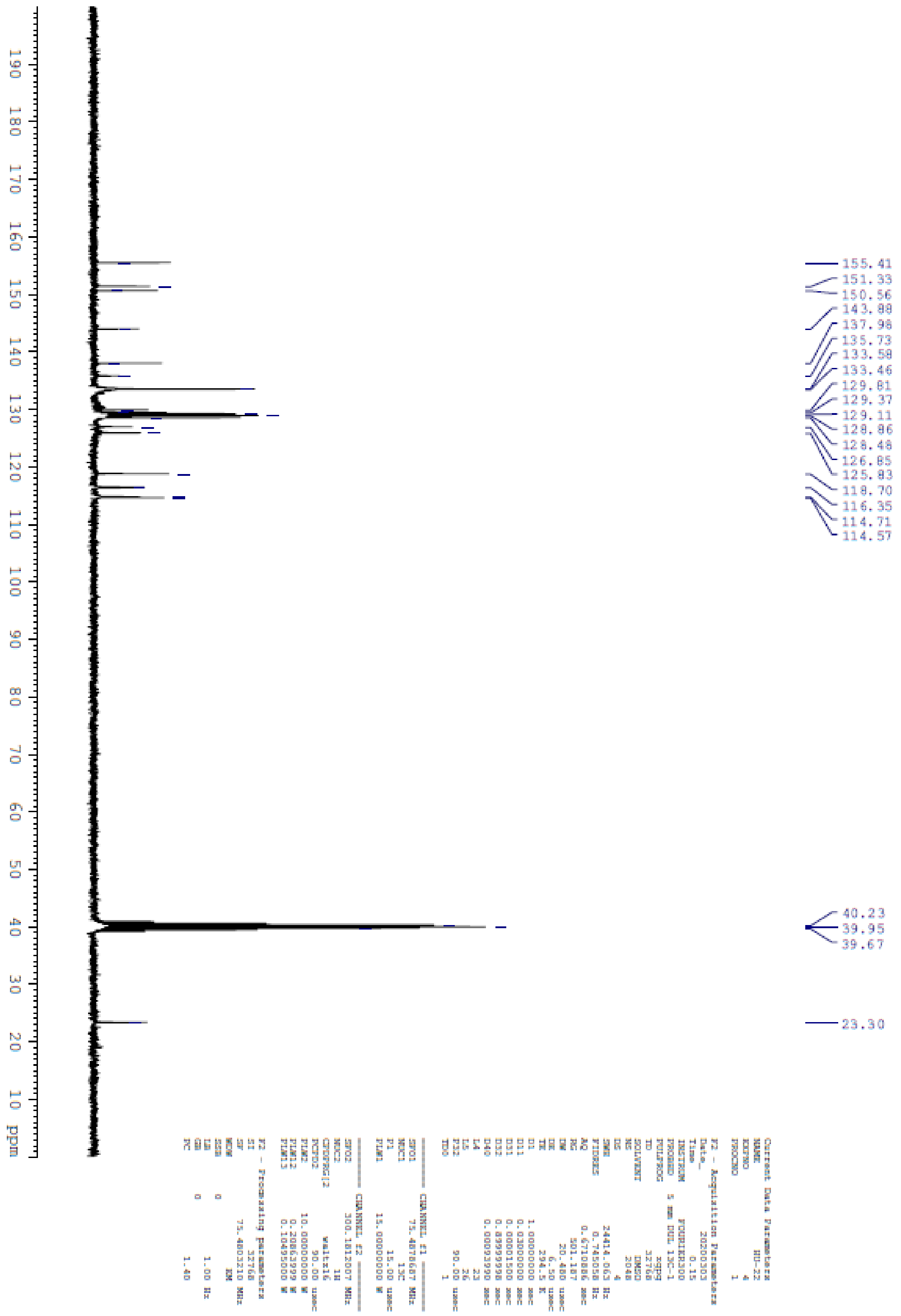

Figure S38. ^{13}C NMR spectrum of compound **5m**

Elmt	Val.	Min	Max	Elmt	Val.	Min	Max	Elmt	Val.	Min	Max	Elmt	Val.	Min	Max	Use Adduct
H	1	14	15	O	2	0	3	Cl	1	0	2	I	3	0	0	H
C	4	23	24	F	1	0	2	Br	1	0	1					
N	3	0	7	S	2	0	2	Ru	2	0	0					

Error Margin (ppm): 5
HC Ratio: unlimited
Max Isotopes: 3
MSn Iso RI (%): 10.00

DBE Range: 10.0 - 30.0
Apply N Rule: yes
Isotope RI (%): 1.00
MSn Logic Mode: AND

Electron Ions: both
Use MSn Info: no
Isotope Res: 10000
Max Results: 500

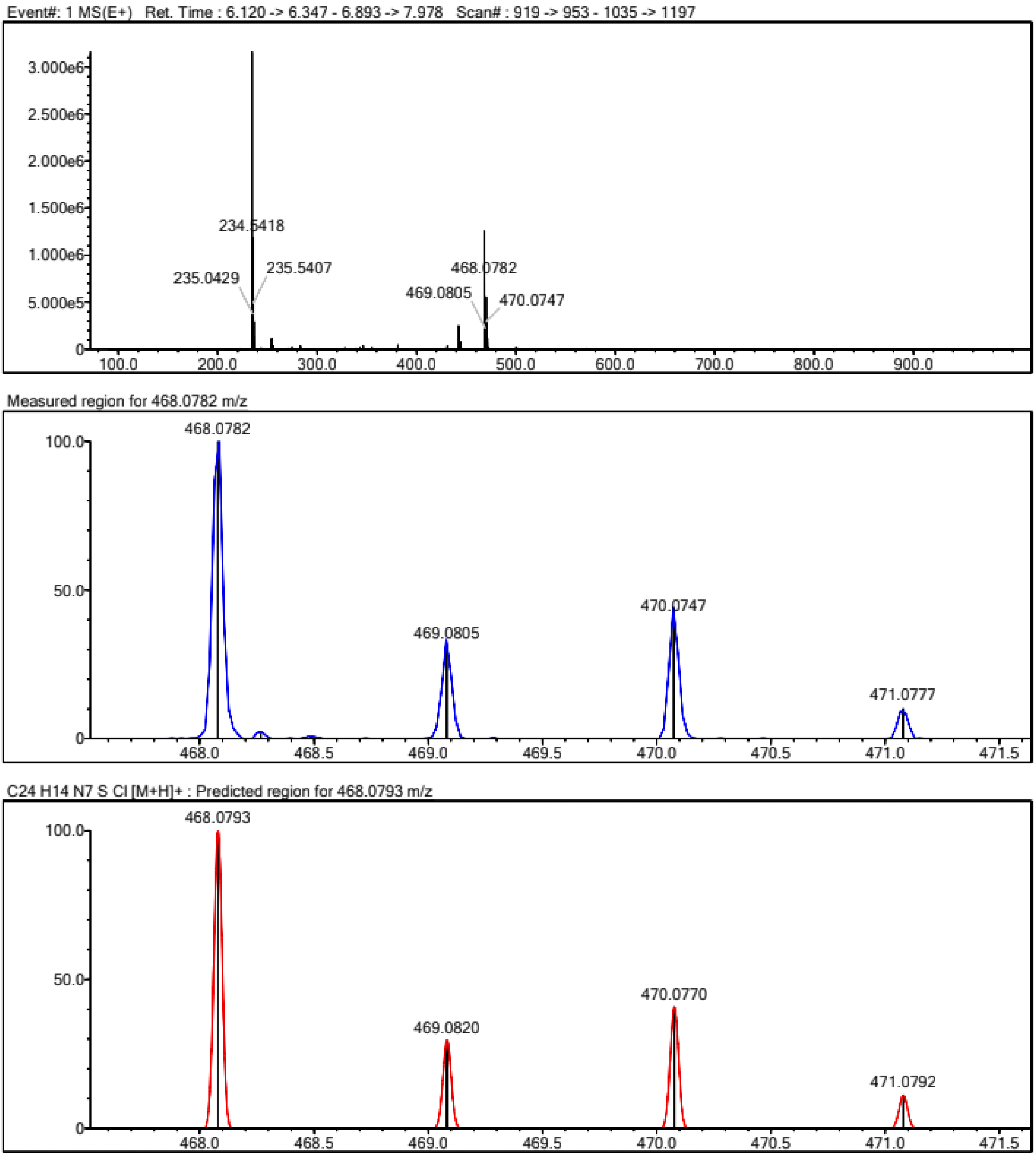

Figure S39. Mass spectrum of compound **5m**

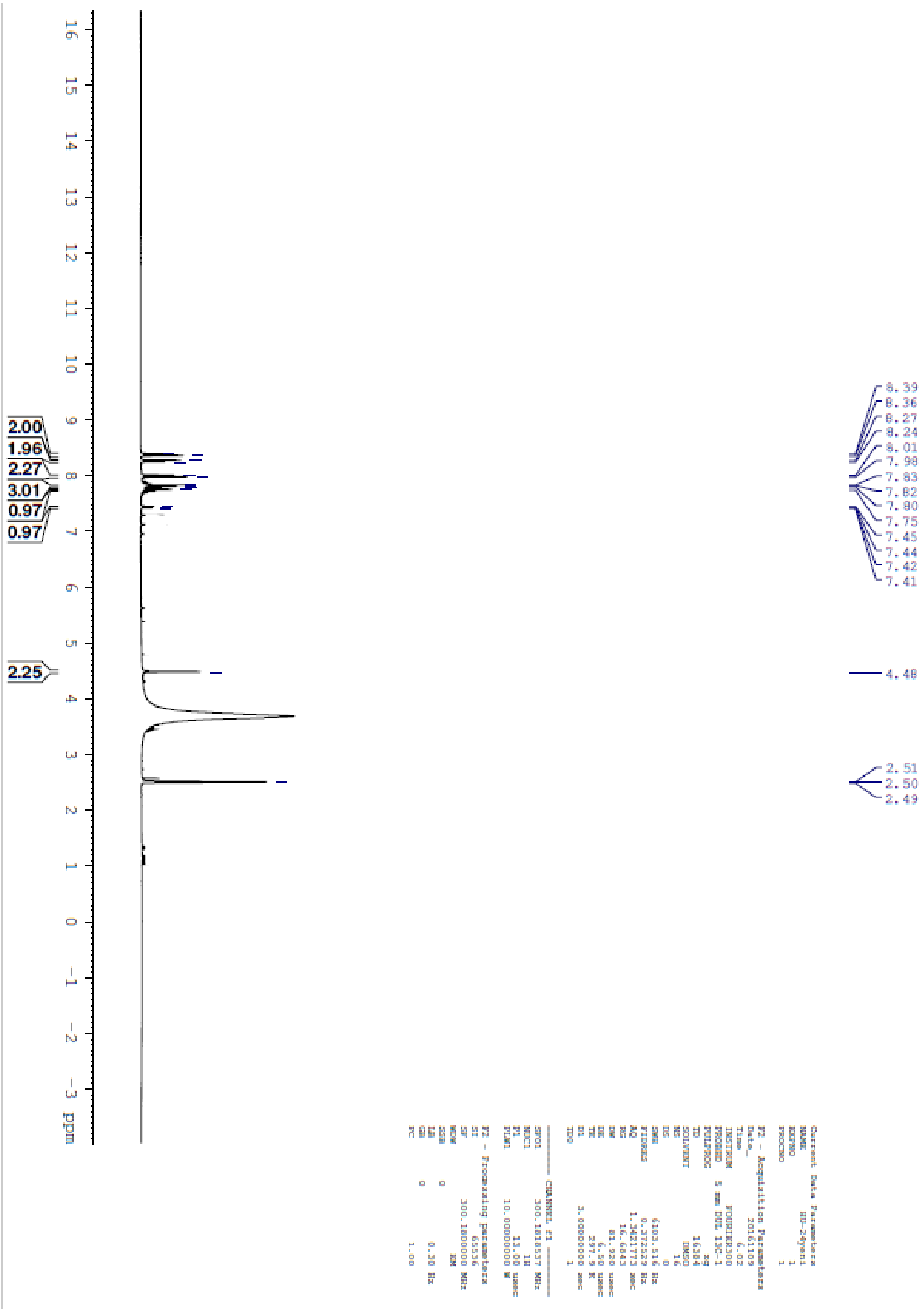

Figure S40. ^{1}H NMR spectrum of compound **5n**

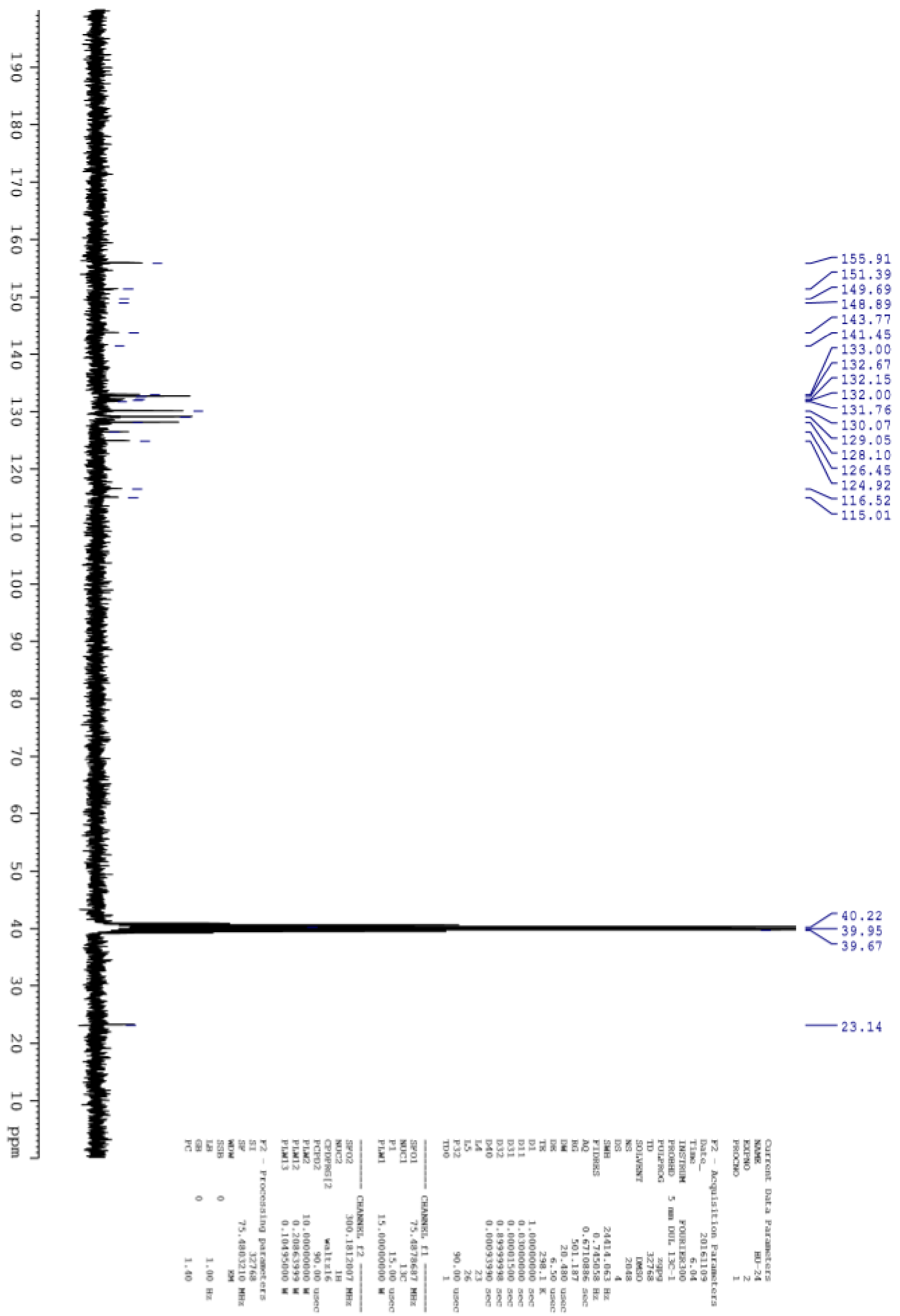

Figure S41. ^{13}C NMR spectrum of compound **5n**

Elmt	Val.	Min	Max	Elmt	Val.	Min	Max	Elmt	Val.	Min	Max	Elmt	Val.	Min	Max	Use Adduct
H	1	14	15	O	2	0	0	Cl	1	0	2	I	3	0	0	H
C	4	23	24	F	1	0	2	Br	1	0	1					
N	3	6	7	S	2	0	1	Ru	2	0	0					

Error Margin (ppm):	5	DBE Range:	10.0 - 30.0	Electron Ions:	both
HC Ratio:	unlimited	Apply N Rule:	yes	Use MSn Info:	no
Max Isotopes:	3	Isotope RI (%):	1.00	Isotope Res:	10000
MSn Iso RI (%):	10.00	MSn Logic Mode:	AND	Max Results:	500

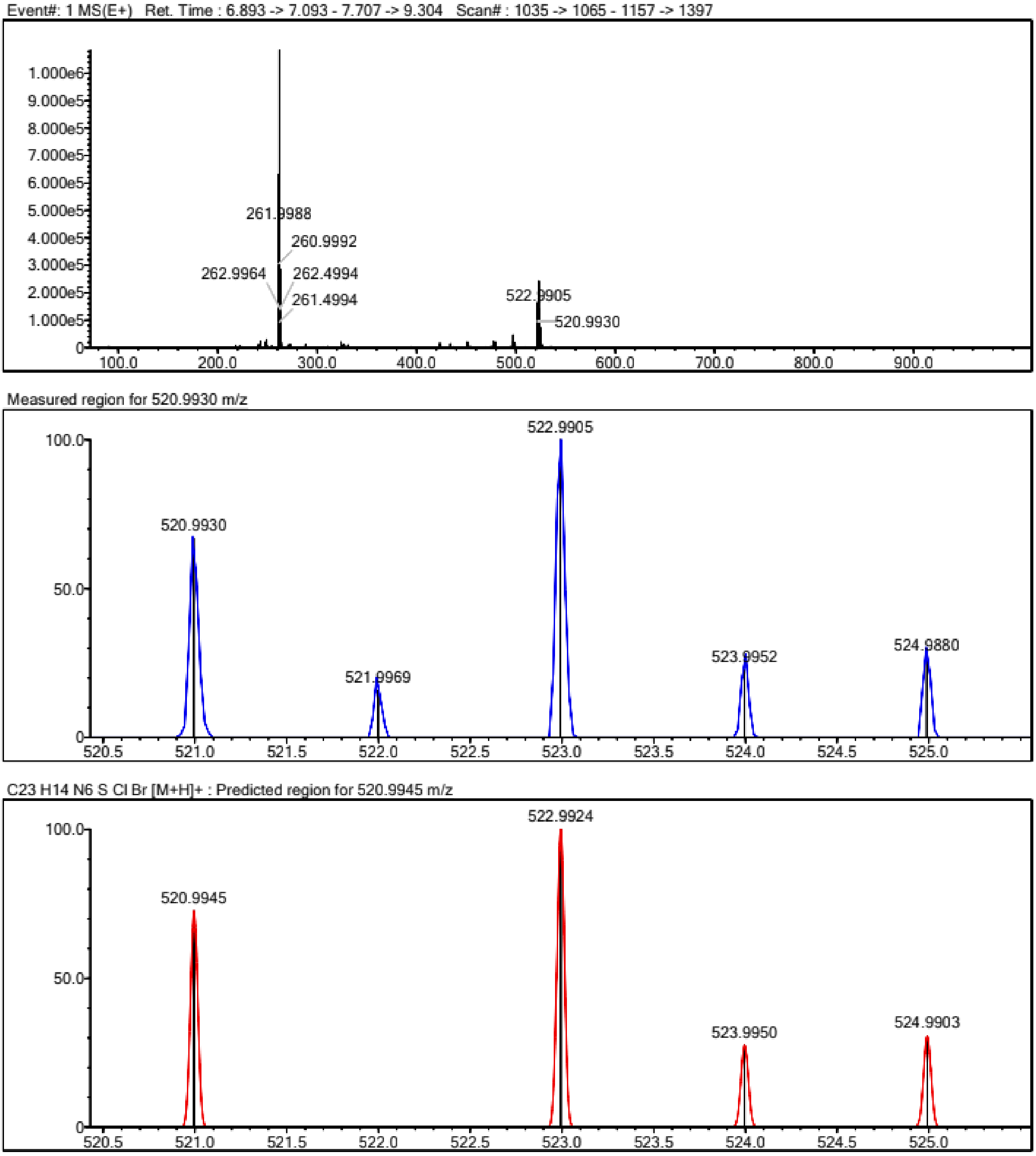

Figure S42. Mass spectrum of compound **5n**

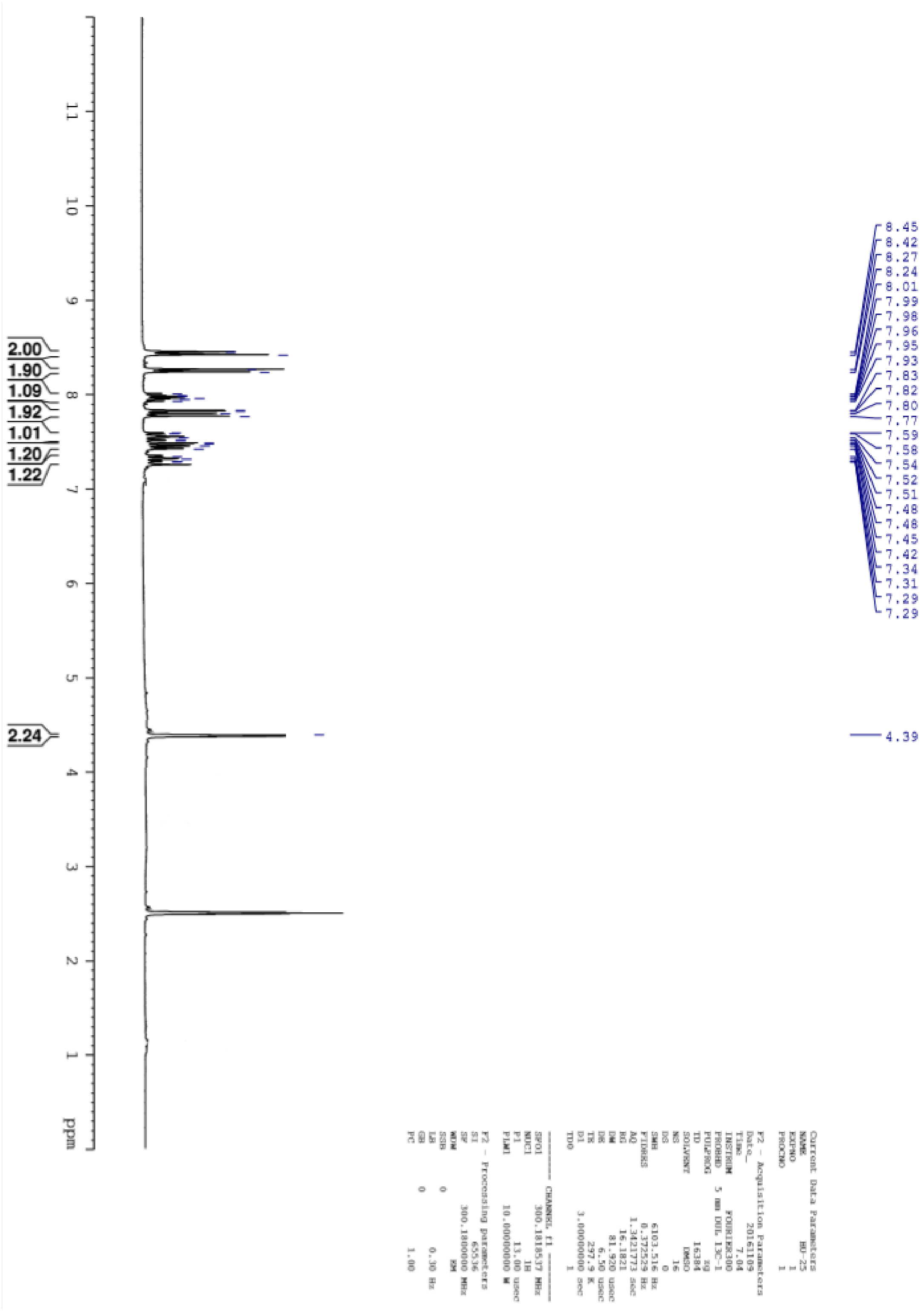

Figure S43. ¹H NMR spectrum of compound **5o**

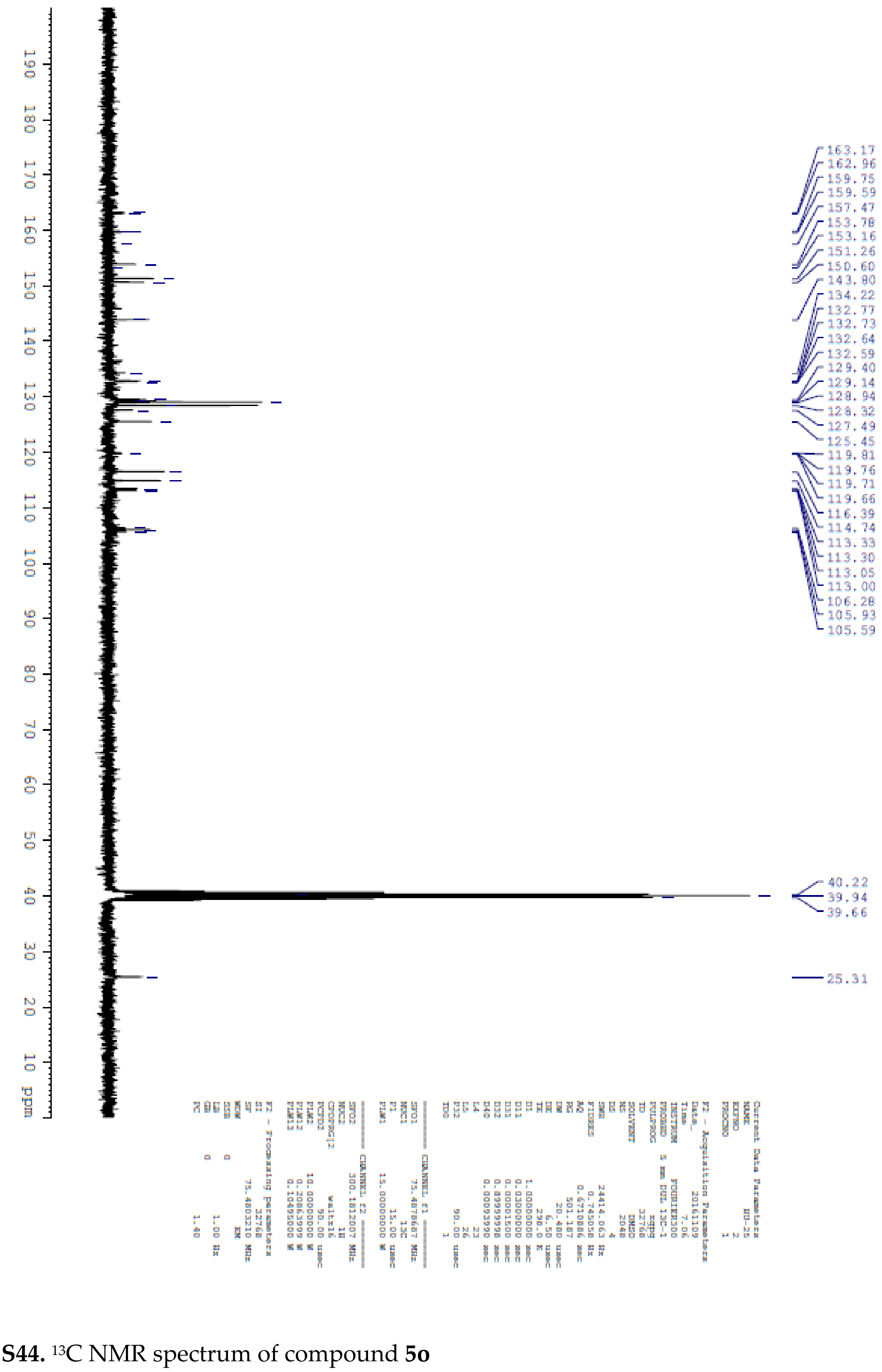

Figure S44. ^{13}C NMR spectrum of compound **5o**

Elmt	Val.	Min	Max	Elmt	Val.	Min	Max	Elmt	Val.	Min	Max	Elmt	Val.	Min	Max	Use Adduct
H	1	13	15	O	2	0	0	Cl	1	0	2	I	3	0	0	H
C	4	23	24	F	1	0	2	Br	1	0	1					
N	3	6	7	S	2	0	1	Ru	2	0	0					

Error Margin (ppm): 5
HC Ratio: unlimited
Max Isotopes: 3
MSn Iso RI (%): 10.00

DBE Range: 10.0 - 30.0
Apply N Rule: yes
Isotope RI (%): 1.00
MSn Logic Mode: AND

Electron Ions: both
Use MSn Info: no
Isotope Res: 10000
Max Results: 500

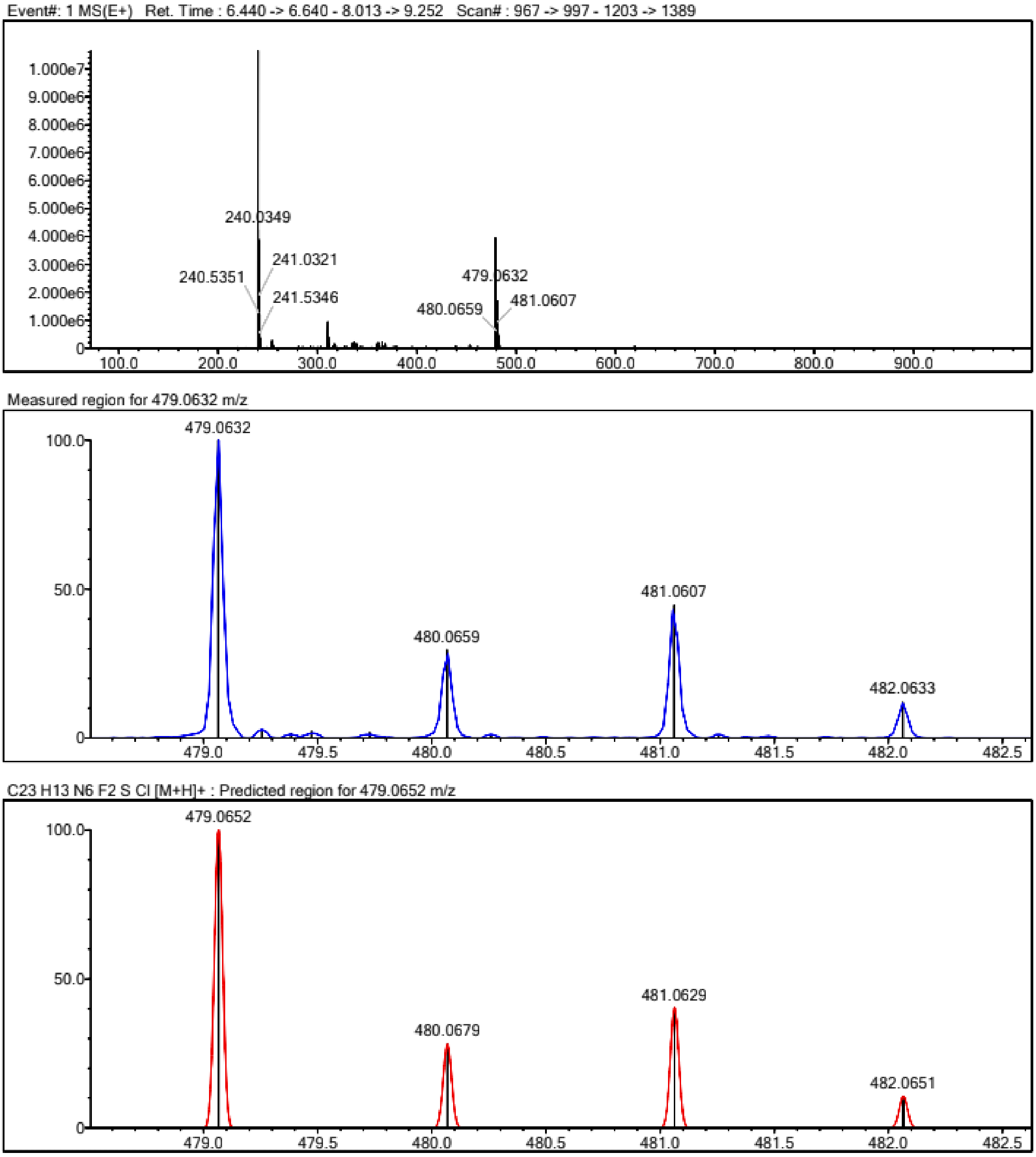

Figure S45. Mass spectrum of compound **5o**

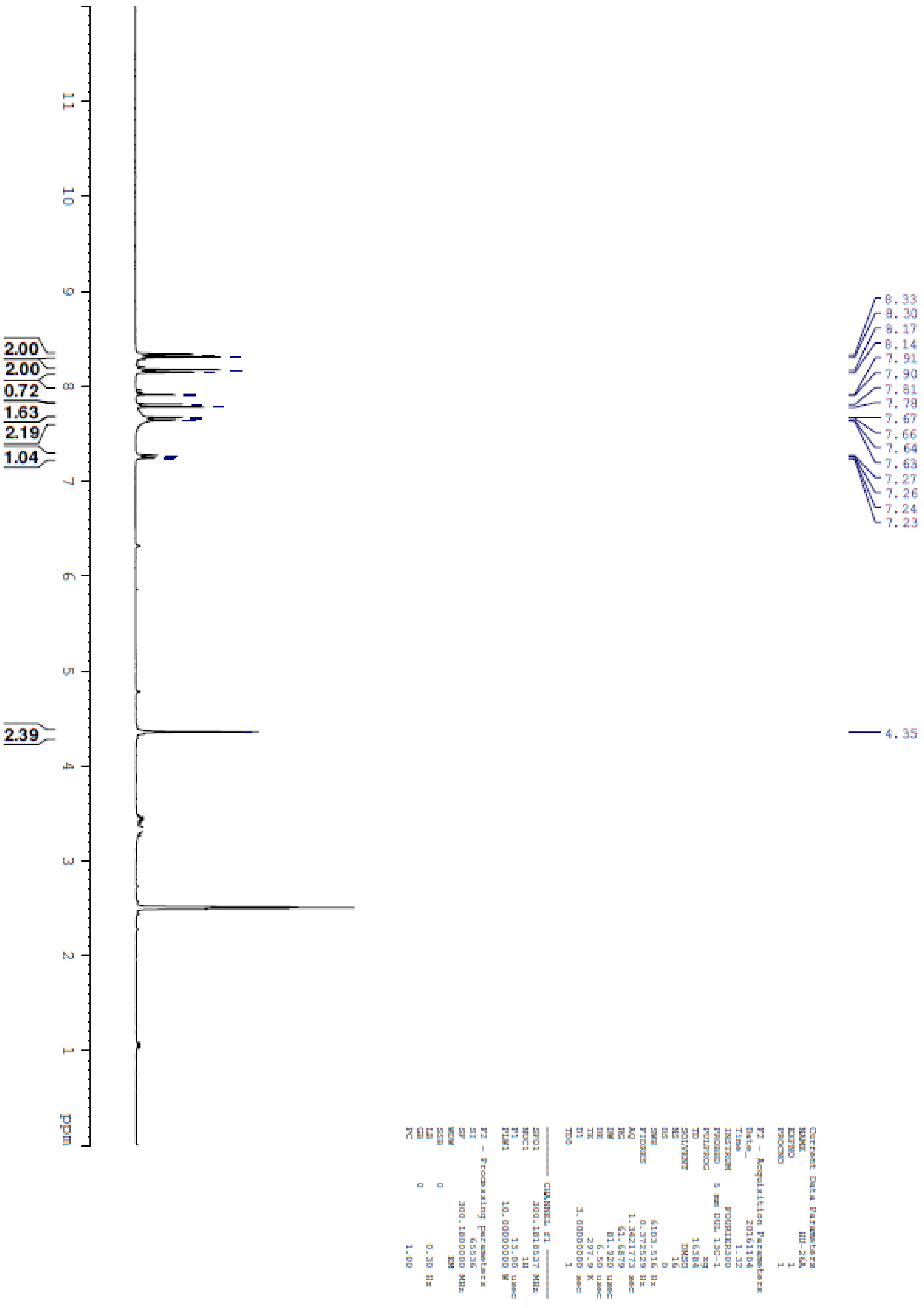

Figure S46. ^{1}H NMR spectrum of compound **5p**

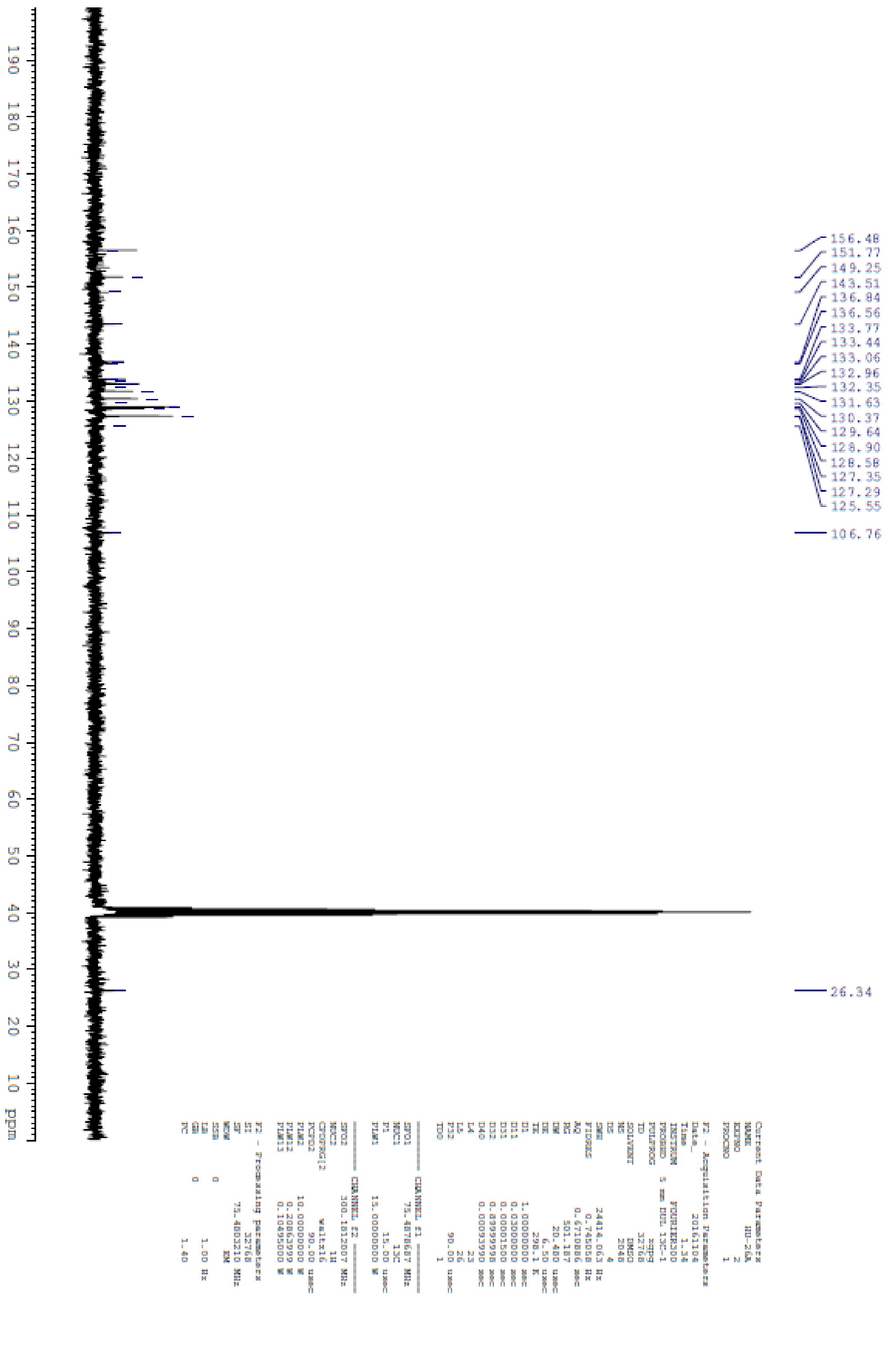

Figure S47. ^{13}C NMR spectrum of compound **5p**

Elmt	Val.	Min	Max	Elmt	Val.	Min	Max	Elmt	Val.	Min	Max	Elmt	Val.	Min	Max	Use Adduct
H	1	13	15	O	2	0	0	Cl	1	0	3	I	3	0	0	H
C	4	23	24	F	1	0	2	Br	1	0	1					
N	3	6	7	S	2	0	1	Ru	2	0	0					

Error Margin (ppm):	5	DBE Range:	10.0 - 30.0	Electron Ions:	both
HC Ratio:	unlimited	Apply N Rule:	yes	Use MSn Info:	no
Max Isotopes:	3	Isotope RI (%):	1.00	Isotope Res:	10000
MSn Iso RI (%):	10.00	MSn Logic Mode:	AND	Max Results:	500

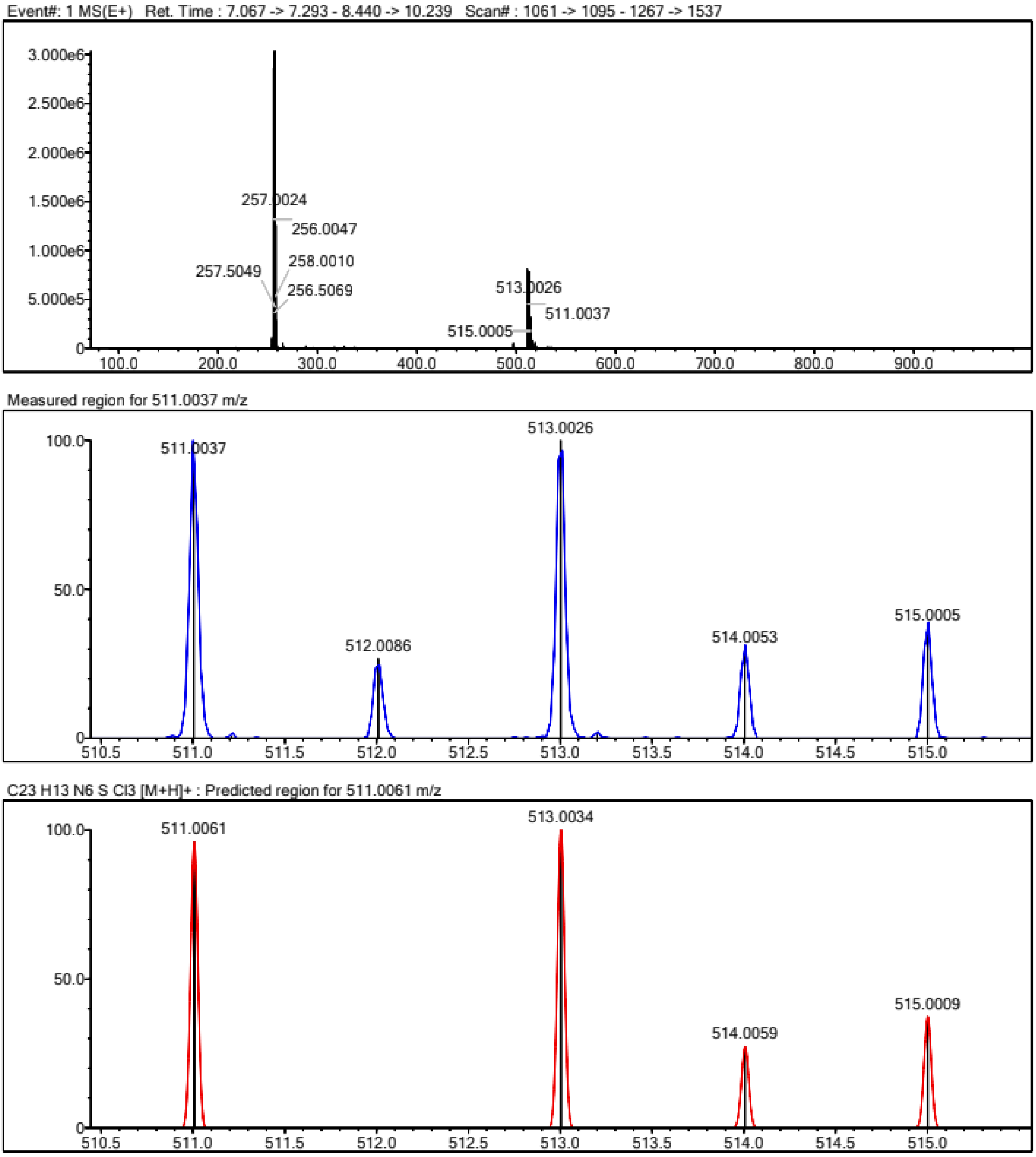

Figure S48. Mass spectrum of compound **5p**
